# Visible‐Light Promoted C–O Bond Formation with an Integrated Carbon Nitride–Nickel Heterogeneous Photocatalyst

**DOI:** 10.1002/ange.202016511

**Published:** 2021-03-03

**Authors:** Arjun Vijeta, Carla Casadevall, Souvik Roy, Erwin Reisner

**Affiliations:** ^1^ Department of Chemistry University of Cambridge Lensfield Road Cambridge CB2 1EW UK; ^2^ Current address: School of Chemistry University of Lincoln Joseph Banks Laboratories Lincoln LN6 7DL UK

**Keywords:** carbon nitride, cross-coupling reaction, dual catalysis, heterogeneous catalysis, photocatalysis

## Abstract

Ni‐deposited mesoporous graphitic carbon nitride (Ni‐mpg‐CN_*x*_) is introduced as an inexpensive, robust, easily synthesizable and recyclable material that functions as an integrated dual photocatalytic system. This material overcomes the need of expensive photosensitizers, organic ligands and additives as well as limitations of catalyst deactivation in the existing photo/Ni dual catalytic cross‐coupling reactions. The dual catalytic Ni‐mpg‐CN_*x*_ is demonstrated for C–O coupling between aryl halides and aliphatic alcohols under mild condition. The reaction affords the ether product in good‐to‐excellent yields (60–92 %) with broad substrate scope, including heteroaryl and aryl halides bearing electron‐withdrawing, ‐donating and neutral groups. The heterogeneous Ni‐mpg‐CN_x_ can be easily recovered from the reaction mixture and reused over multiple cycles without loss of activity. The findings highlight exciting opportunities for dual catalysis promoted by a fully heterogeneous system.

## Graphical abstract

A Ni‐deposited carbon nitride material was developed as a fully heterogeneous dual photocatalyst. Visible light‐driven C–O cross‐coupling is demonstrated free of organic ligands and additives. This dual catalytic system operates with very low nickel loadings and the heterogeneous photocatalyst can be easily recycled.

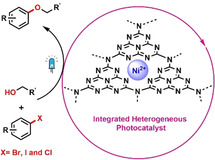

The development of sustainable methodologies for organic synthesis is essential to establish a green chemical industry in a future circular economy. Transition‐metal‐catalyzed cross‐coupling reactions are essential tools in fine‐chemical synthesis, predominately employing homogeneous palladium catalysts.[^1^, ^2^] Abundant nickel has emerged as a more sustainable alternative,^[3]^ but its lower electronegativity makes the reductive elimination (RE) step challenging,^[4]^ particularly in the coupling reactions of carbon and an electronegative heteroatom to form C−O and C−N bonds.

Recent studies have demonstrated that the RE step can be enhanced by combining a photosensitizer with nickel catalysis via a dual photocatalysis approach (Scheme 1 a).[^5^, ^6^] The strategy involves: i) photoinduced electron transfer from the light absorber to the Ni catalyst yielding a reduced Ni^I^ species that can undergo oxidative addition to generate a high energy Ni^III^ species, or ii) oxidative addition to Ni^0^ generating a Ni^II^ intermediate that produces an excited‐state Ni*^II^ species upon light‐induced energy transfer from the photosensitizer. These Ni^III^ or Ni*^II^ species can then thermodynamically drive the RE step efficiently.[^7^, ^8^, ^9^] This dual catalysis approach has not only facilitated traditional cross‐coupling reactions, but also enabled direct coupling of radical species generated under mild reaction conditions.[^10^, ^11^] However, this approach has been mostly limited to precious Ru‐ and Ir‐based homogeneous photocatalysts.^[12]^

**Scheme 1:**
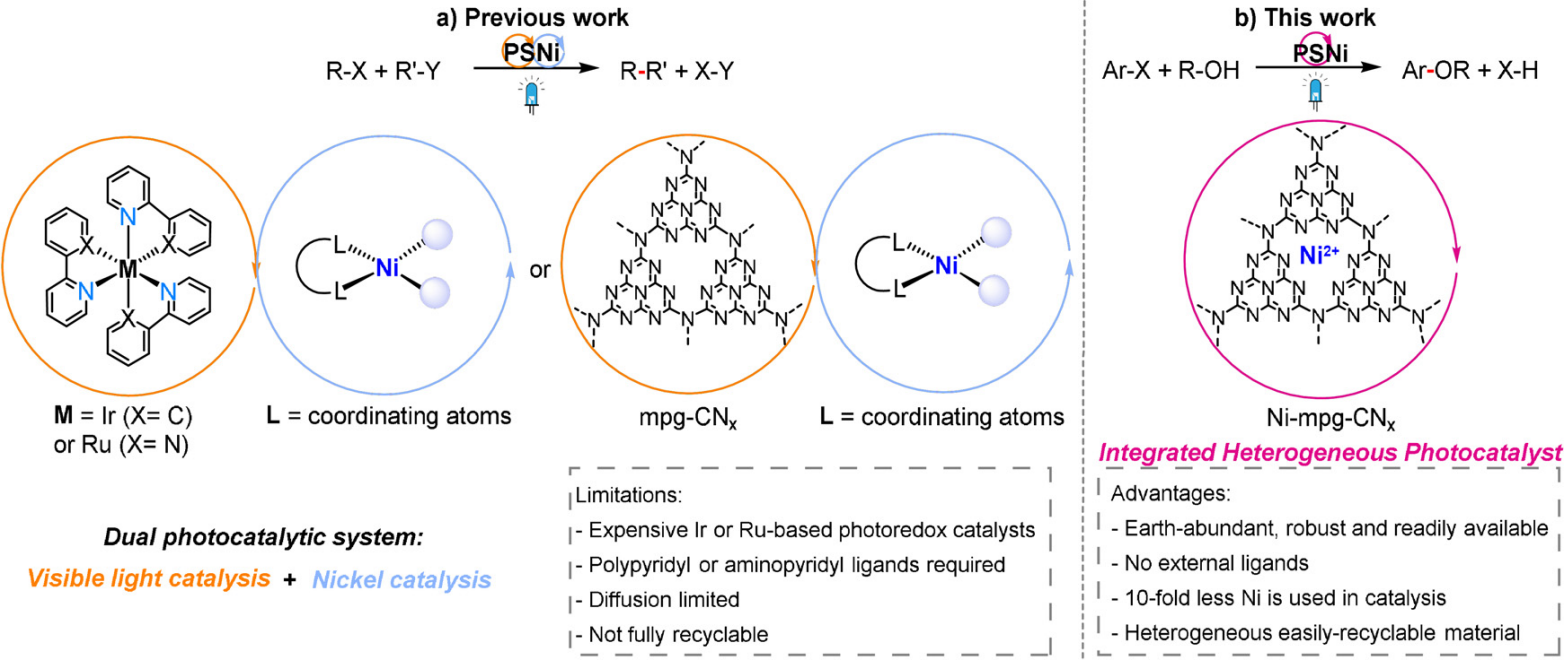
Comparison between a) state‐of‐the‐art homogeneous Ni dual systems for photoredox catalysis and b) the integrated Ni‐mpg‐CN_*x*_ heterogeneous photocatalyst for C–O coupling reported in this study.

Carbon nitride (CN_*x*_) has emerged as a promising light‐harvesting material for applications in dual nickel/photocatalysis owing to its low‐cost, non‐toxicity and facile synthesis (Scheme 1 a).[^13^, ^14^] The tunable redox potential, excellent photostability and light harvesting ability of CN_*x*_ make it an appealing candidate for photocatalysis,^[15]^ which has already been proven useful for challenging organic transformations,[^16^, ^17^, ^18^] solar water splitting[^19^, ^20^] and CO_2_ reduction reactions.[^21^, ^22^, ^23^] However, nickel‐based catalysis with CN_x_ relies on a homogenous coordination complex such as pyridine‐based Ni catalysts, which introduces fragility, diffusion‐limited charge transfer, gradual formation of inactive nickel‐black and challenges in product isolation.^[24]^

The heptazine‐units and amine functional groups within CN_x_ contain intrinsic coordination sites, which provide a robust scaffold for binding Ni^2+^ and enable direct electronic communication between the light‐harvesting units and Ni‐active sites.[^25^, ^26^] Previous studies have shown that metal doping in CN_*x*_ improves the photocatalytic activity of CN_*x*_ for solar fuels production by facilitating charge separation.[^27^, ^28^] Despite its potential to enhance activity, robustness, and recyclability, the synergic effect of Ni‐deposition on CN_*x*_ is still underexplored in organic synthesis. Only a very recent report demonstrated an assembly of carbon nitride with Ni as a dual catalytic system for C–O coupling, but it still required imidazole as an auxiliary ligand for the activating Ni catalysis, and quinuclidine as a sacrificial electron donor.^[29]^

Herein, we report nickel‐deposited mesoporous carbon nitride (Ni‐mpg‐CN_*x*_) as an integrated single‐entity photocatalyst to perform organic C–O coupling reactions between simple alcohols and various aryl halides under visible‐light irradiation (Scheme 1 b). Kinetic studies provide mechanistic insights into the dual catalytic role of Ni‐mpg‐CN_*x*_ and the robustness and applicability of Ni‐mpg‐CN_*x*_ is demonstrated by continuous recycling experiments.

Mpg‐CN_*x*_ has been synthesized following a slightly modified reported procedure by heating cyanamide with silica as a hard template in air at 550 °C, followed by etching with aqueous ammonium difluoride.^[23]^ Ni‐mpg‐CN_*x*_ was prepared by heating a mixture of NiCl_2_ and mpg‐CN_*x*_ in acetonitrile at 80 °C under microwave treatment in the presence of triethylamine to facilitate the deposition of Ni. For comparative studies, Ni^2+^ was also deposited on two other types of carbon nitride (non‐mesoporous CN_*x*_ and NCN‐functionalized CN_*x*_) under the same conditions (see Experimental Details in the Supporting Information).

The deposition of Ni on mpg‐CN_*x*_ was confirmed by inductively coupled plasma optical emission spectrometry (ICP‐OES), X‐ray photoelectron spectroscopy (XPS), scanning electron microscopy (SEM), and transmission electron microscopy (TEM) (Figure 1 and Figures S1–S7). ICP‐OES shows that 4.14±0.99 wt % Ni was deposited on mpg‐CN_x_ (Table S1). The XPS spectrum of Ni‐mpg‐CN_*x*_ consists of two peaks in the Ni 2p region at 855 (Ni 2p_3/2_) and 872 eV (Ni 2p_1/2_), which confirm the presence of Ni^2+^ sites on mpg‐CN_*x*_ (Figure 1 A). The absence of a peak at ≈852 eV indicates the absence of a metallic Ni^0^ species.^[30]^ The N 1 s peak of unmodified mpg‐CN_*x*_ at ≈398 eV can be deconvoluted into three peaks centered at 397.0, 398.6 and 399.6 eV, assigned to C=N‐C (pyridinic N), N(C)_3_ (quaternary N) and C‐N‐H (uncondensed amine) (Figure S6).^[31]^ Upon nickel modification, a new peak appears in the N 1s region at 397.9 eV, which can be tentatively attributed to Ni‐coordinated pyridinic N.[^32^, ^33^] The C 1s XPS spectra of mpg‐CN_*x*_ and Ni‐mpg‐CN_*x*_ could be deconvoluted into near‐identical components without any significant shifts (Figure S7). Analysis of the composition of the near‐surface region by XPS shows a lower Ni content of 2.2 wt % at the catalyst surface. Energy‐dispersive X‐ray (EDX) analysis of TEM images indicates uniform distribution of Ni throughout the material (Figure 1 B,C). Powder X‐ray diffraction (pXRD), attenuated total reflectance infrared (ATR‐IR) and UV/Vis spectra of Ni‐mpg‐CN_*x*_ are similar to bare mpg‐CN_*x*_, suggesting that Ni deposition did not affect the optical properties and composition of the material (Figure S8–S10). Overall, we speculate that Ni‐mpg‐CN_*x*_ features active Ni^2+^ sites coordinated to the pyridinic groups in CN_*x*_ and the material retains the photocatalytic activity of the parent mpg‐CN_*x*_.

**Figure 1:**
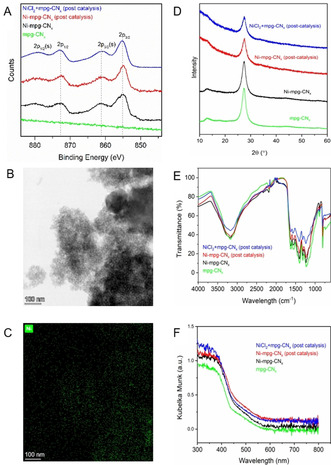
A) Normalized XPS Ni_2p_ region; (s) denotes satellite peak. B) TEM image with C) EDS mapping of Ni in Ni‐mpg‐CN_*x*_. D) pXRD, E) ATR‐IR and F) UV/Vis DRS of mpg‐CN_x_, Ni‐mpg‐CN_*x*_ (as synthesized), Ni‐mpg‐CN_*x*_ post‐catalysis, and Ni‐mpg‐CN_*x*_ obtained after performing catalysis with NiCl_2_ (salt)+mpg‐CN_*x*_.

Our investigation into C–O coupling using Ni‐mpg‐CN_*x*_ powder as an integrated solid‐state photocatalyst started with 4‐bromobenzonitrile in ethanol, which also serves as a coupling partner, with sodium hydroxide as a base (Table 1). The desired coupling product **1** was obtained in 75 % yield upon irradiation of the reaction mixture in a blue LED photoreactor (*λ*=447±20 nm, 1.03 W @ 700 mA per LED)^[34]^ for 18 hours under an inert atmosphere (entry 1). Other Ni‐deposited CN_*x*_ materials with similar optical and redox properties provided a significantly lower yield of **1** (28 % for pristine CN_*x*_ and 8 % for ^NCN^CN_*x*_, entries 2 and 3, Table 1).^[35]^ The superior activity of mpg‐CN_*x*_ can be attributed to its higher surface area, greater number of defects and available pyridyl as well as amine groups for Ni coordination.^[36]^

**Table 1:** Screening of carbon nitride, co‐catalyst and control experiments.

Entry	Deviation	Product^[a]^
1	none	75 % (73 %)^[b]^
2	Ni‐CN_*x*_	28 %
3	Ni‐^NCN^CN_*x*_	8 %
4	NiCl_2_ (1 wt %) + mpg‐CN_*x*_	61 %
5	NiCl_2_ (2 wt %) + mpg‐CN_*x*_	70 %
6	NiCl_2_ (5 wt %) + mpg‐CN_*x*_	70 %
7	FeCl_2_ (5 wt %) + mpg‐CN_*x*_	n.d.
8	CoCl_2_ (5 wt %) + mpg‐CN_*x*_	n.d.
9	no light	<1 %
10	no base	<1 %
11	no photocatalyst	n.d.
12	mpg‐CN_*x*_	2 %
13	NiCl_2_ (5 mol %)	n.d.

Standard conditions: 4‐bromobenzonitrile (100 mM), Ni‐mpg‐CN_x_ (5 mg), [NaOH] (200 mM in EtOH, 2 mL), 420 min irradiation at *λ*=447±20 nm and 40 °C under N_2_. NiCl_2_ wt % refers to weight percentage of mpg‐CN_x_, n.d.=not detected. [a] ^1^H‐NMR yield using 1,3,5‐trimethoxybenzene (50 μmol) as an internal standard. [b] Isolated yield.

Performing the reaction upon separate addition of mpg‐CN_*x*_ and different wt % of NiCl_2_ resulted in a similar yield of **1** (entries 4–6, Table 1). This result shows that adding NiCl_2_ in situ leads to its deposition on mpg‐CN_x_ as confirmed by ICP‐OES (Table S1) and post‐catalysis characterization by XPS, TEM, pXRD, ATR‐IR and UV/Vis spectra confirmed the formation of Ni‐mpg‐CN_*x*_ (Figure 1 and Figures S2 and S5).^[13]^ The amount of NiCl_2_ (2 wt %, 0.085 mol %) used here is >100 times less than the generally used amount for homogeneous Ni catalyst (10 mol %) in dual catalysis. This approach provides us with an alternative and straightforward route to the synthesis of an integrated Ni‐mpg‐CN_*x*_ photocatalyst. Replacing NiCl_2_ with Co and Fe salts showed no reactivity (entries 7 and 8, Table 1). The use of other commonly used solvents resulted either in a lower yield or various side products (Table S2). Various other organic and inorganic bases resulted in a reduced yield (Table S3), whereas lowering the loading of NaOH from 2 to 1.2 equivalent slightly improved the yield of **1** by 7 % (Table S4). Control experiments showed that no significant cross‐coupling product was observed in the absence of light, base, mpg‐CN_*x*_ or Ni (entries 9–13, Table 1).

We subsequently investigated the scope of the C–O coupling reaction using different aryl and heteroaryl halides (Scheme 2). The para‐substituted electron‐deficient bromoarenes containing nitrile, ketone, aldehyde, ester, amide, and sulfone substituents provide the coupling products **2**–**8** in 78–92 % yield. The high yield may be the result of the enhanced reaction rate that reduces the formation of byproducts. Small amounts of dehalogenated product, the corresponding phenol and occasional hydrolysis of the functional group on the aryl halides are commonly observed byproducts during this coupling reaction. The *ortho‐* and *meta*‐acetyl substituted bromobenzene (**9** and **10**) were also tolerable, but reacted slower than the *para*‐analogue (**3**) and the presence of acetyl group at *ortho*‐position did not allow the reaction to go to full conversion.^[13]^ An additional bromo‐ and chloro‐substituted aryl bromide gave the monosubstituted ether products **11** and **12**, respectively, without any 1,4‐diether product as the resulting etherification product deactivates the aryl halide for the second etherification. The reaction also proceeds with electron‐rich aryl bromides but requires a significantly longer reaction time to form **13** and **14**. Heteroaryl bromide including pyridine, quinoline and benzofuranone have effectively participated in the reaction to give a good yield of the corresponding coupling products **15**–**17**. With respect to the alcohol substrate, we observed reactivity for primary alcohols including ethanol and n‐propanol to yield **1** and **18**. The deuterated aryl ether **19** can also be prepared using the Ni‐mpg‐CN_*x*_ photocatalyst. Overall, the observed reactivity trend correlates with other transition metal catalyzed coupling reactions.^[37]^

**Scheme 2:**
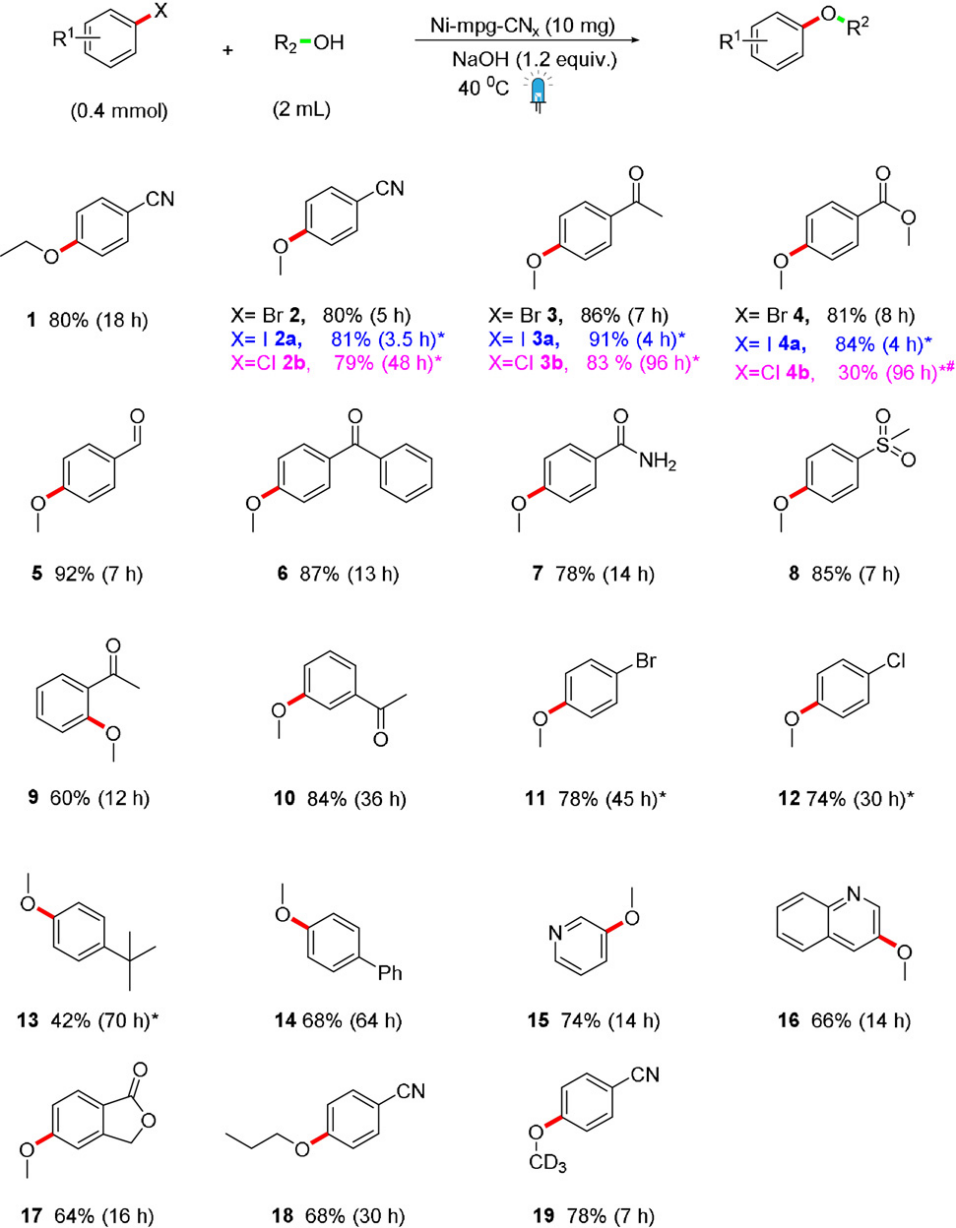
Substrate scope for C–O coupling. Isolated yields are given, except where * indicates NMR yields. ^#^Indicates where hydrolysis of the ester bond is the major byproduct. Standard catalytic conditions: Aryl halide (200 mM), Ni‐mpg‐CN_*x*_ (10 mg), [NaOH] (240 mM in alcohol, 2 mL) irradiation at *λ*=447±20 nm and 40 °C under N_2_.

We further studied the coupling protocol between methanol and various aryl iodide and chlorobenzene substrates. Consistent with homogeneous transition metal catalysis reactions, a general inverse correlation between reaction rate and strength of C−X bond (I< Br < Cl) was observed for a given functional group of aryl halides towards the coupling reaction. Consequently, the aryl iodides were the fastest to react to give the coupling products **2 a**–**4 a** followed by aryl bromide coupling products **2**–**4** and then the aryl chlorides **2 b**–**4 b**. Chlorobenzenes required 60 °C to achieve full conversion (Scheme 2).

The use of an integrated Ni‐mpg‐CN_*x*_ photocatalyst powder allowed for easy and quantitative recovery (up to 94 % recovered material per run) of the Ni‐mpg‐CN_*x*_ after catalysis from the reaction crude upon simple centrifugation–washing cycles (see Supporting Information for details). The recovered heterogeneous material could be reused for further cycles to give coupling product **3** with no need of re‐addition of any of the catalytic components (Ni or mpg‐CN_*x*_) and without loss of activity up to three cycles after which a slower rate of coupling was observed (Figure 2). The decrease in rate/activity could be due to an overall leaching of 20 % Ni from Ni‐mpg‐CN_*x*_ after the fourth recycling or migration of surface‐activated Ni species to the bulk (Table S1).^[38]^ The recovered material displayed unchanged spectroscopic features and pXRD diffraction pattern (Figure 1).

**Figure 2:**
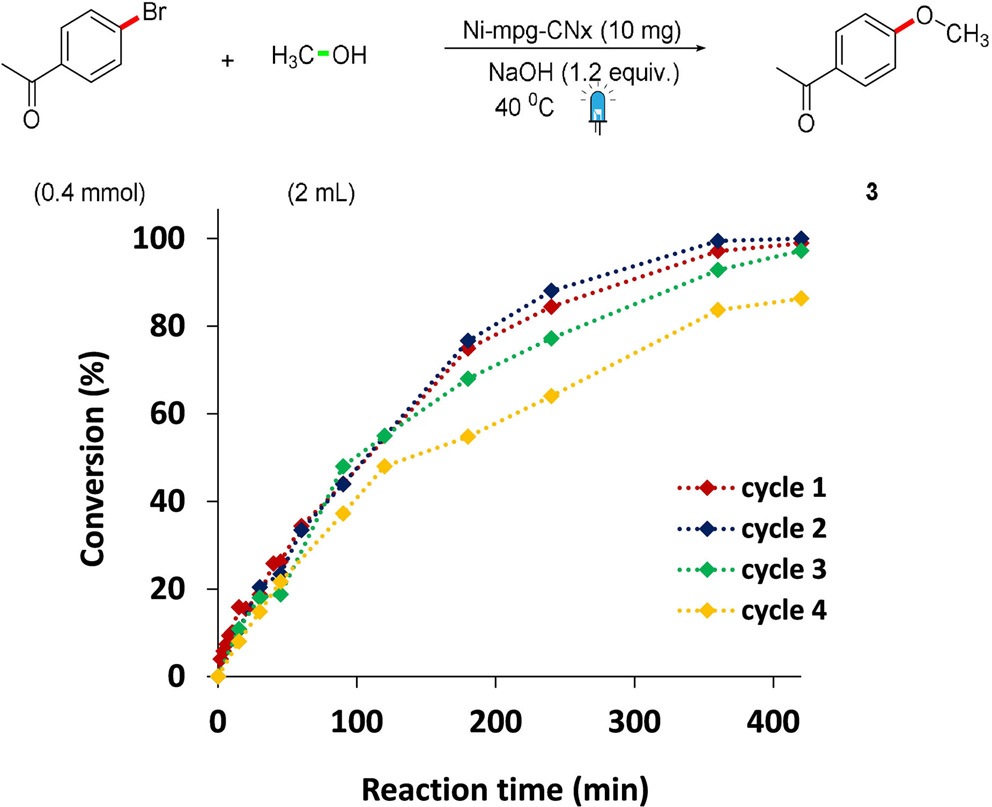
Recycling studies of the C–O coupling reaction. Catalytic conditions: 4‐bromoacetophenone (200 mM), Ni‐mpg‐CN_*x*_ (10 mg), [NaOH] (240 mM in MeOH, 2 mL), 420 min irradiation at *λ*=447±20 nm and 40 °C under N_2_.

Mechanistic studies were performed using 3‐bromoacetophenone as a model substrate (Figures S11‐13 and section 3 in the Supporting Information). First, kinetic studies varying the concentration of the substrate while keeping other parameters constant showed a slope 0.77±0.04 on plotting ln *k* versus ln[substrate]. Although a more complex mechanism cannot be excluded, this suggests an approximately first order rate at initial reaction times (first 10 min, Figure S11). This is in agreement with the first step being the oxidative addition of the substrate to the reduced Ni‐mpg‐CN_*x*_ (Figure 3). Kinetic analysis based on the Eyring equation shows a kinetic barrier (2.81±0.31 kcal mol^−1^, Table S6) that agrees with the observed fast reaction times at 40 °C (Figure 2). Moreover, the negative value obtained for Δ*S* ^≠^ (−70.0±1 cal mol^−1^ K^−1^) supports an associative mechanism.[^11^, ^39^]

**Figure 3:**
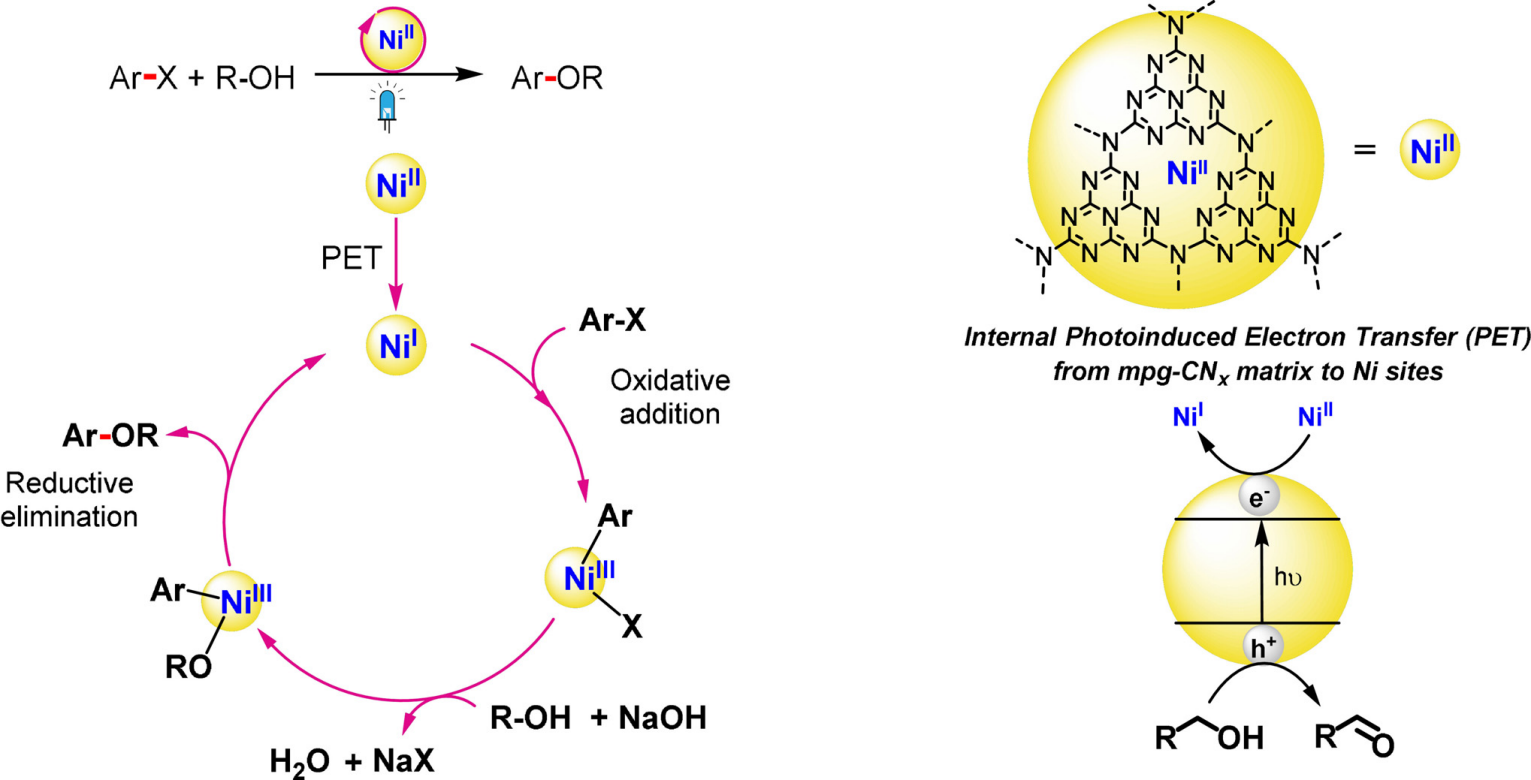
Proposed catalytic cycle for the C–O coupling reactions catalyzed by Ni‐mpg‐CN_*x*_.

Computational studies at the B3LYP/6‐31G* level of theory have also been performed (see section 4 in the Supporting Information for details) and we propose a catalytic cycle for this transformation based on our experimental evidence, computational calculations and previously reported results with molecular systems (Figure 3). Briefly, upon visible light excitation mpg‐CN_*x*_ generates a photoexcited electron‐hole pair. The photogenerated holes are quenched by the alcohol substrate (potential of mpg‐CN_*x*_ valence band=+1.7 V vs. SHE; conduction band=−1.0 V vs. SHE, and MeOH *E* ^ox^ = +1.54 V vs. SHE).[^17^, ^23^] Simultaneously, the photoexcited electron from the mpg‐CN_*x*_ matrix is transferred to Ni^II^ center to yield Ni^I^ as supported by DFT calculations (*E* ^red^_theor_=0.33 V vs. SHE), since further reduction to Ni^0^ is not accessible according to the DFT calculations (*E* ^red^_theor_=−1.51 V vs. SHE). This is followed by oxidative addition and ligand exchange, where Ni^III^ intermediates are invoked. Finally, reductive elimination completes the cycle (Figure 3 and Schemes S2,S3).[^11^, ^29^]

The external quantum efficiency (EQE) for the coupling reaction with Ni‐mpg‐CN_*x*_ was calculated by assuming that one photon is consumed for a molecule of product (although we note that the number of photons required is not unequivocally known, see below).^[40]^ We obtained an EQE of 2.26±0.1 % at *λ*=447 (see Supporting Information for more detail). Light‐dark experiments showed no product formation in the dark following an irradiation period (Figure S14), which suggests that even though the Ni catalytic cycle is ideally self‐sustainable, continuous irradiation is necessary to avoid the deactivation of Ni^I^/Ni^III^ species by carbon nitride matrix. Further mechanistic studies are ongoing to elucidate the complete photocatalytic cycle in more detail.

In summary, we have developed an inexpensive, robust, and easily synthesizable Ni‐mpg‐CN_*x*_ heterogeneous material to play a dual catalytic role. The Ni deposition was confirmed by ICP, TEM and XPS, also indicates that the nickel is present as a Ni^2+^ active site. UV/Vis, ATR‐IR and SEM images confirms that the morphology, composition, and optical property of mpg‐CN_*x*_ is unaffected by nickel modification. The dual catalysis of Ni‐mpg‐CN_*x*_ is demonstrated for C–O coupling between aryl halides and alcohols. The coupling has demonstrated broad substrate scope with significant functional group tolerance and adaptability to different halides. Moreover, the observed reactivity trend is similar to a homogeneous transition metal catalyzed cross‐coupling reaction. The heterogeneous Ni‐mpg‐CN_*x*_ materials are easily recovered from the reaction mixture and have been reused several times, suggesting a potential application of this material for a dual catalytic approach in larger‐scale industrial chemical synthesis. Finally, kinetic studies have shed some light on the mechanism and suggest an oxidative addition of the aryl halide to the reduced Ni‐mpg‐CN_*x*_ as the rate‐determining step of the reaction. We anticipate that this powdered material will enable a more simple, sustainable, and versatile dual photocatalysis approach in organic synthesis in the future.

## Conflict of interest

The authors declare no conflict of interest.

## Supporting information

As a service to our authors and readers, this journal provides supporting information supplied by the authors. Such materials are peer reviewed and may be re‐organized for online delivery, but are not copy‐edited or typeset. Technical support issues arising from supporting information (other than missing files) should be addressed to the authors.

Supplementary
