# Supplementary material for: Visible‐Light Promoted C–O Bond Formation with an Integrated Carbon Nitride–Nickel Heterogeneous Photocatalyst
Source: Angew Chem Weinheim Bergstr Ger. 2021 Mar 3;133(15):8575–80. doi: 10.1002/ange.202016511 (PMC10947600; doi:10.1002/ange.202016511)
Supplement: Supplementary file 1 — Supplementary [file ANGE-133-8575-s001.pdf]

Angewandte Chemie
Eine Zeitschrift der Gesellschaft Deutscher Chemiker

Supporting Information

Visible-Light Promoted C–O Bond Formation with an Integrated Carbon Nitride–Nickel Heterogeneous Photocatalyst

*Arjun Vijeta, Carla Casadevall, Souvik Roy, and Erwin Reisner**

ange_202016511_sm_miscellaneous_information.pdf

Supporting information

Table of Contents

1. GENERAL INFORMATION........2
2. COUPLING PRODUCTS CHARACTERIZATION........6
3. KINETIC STUDIES........11
4. COMPUTATIONAL STUDIES........13
5. TABLES........17
6. FIGURES........21
7. NMR SPECTRA OF PURIFIED PRODUCTS........30
8. REFERENCES........57

1. General information

Reagents used throughout this study were of the highest available purity acquired from commercial suppliers and used directly without any further purification, unless mentioned otherwise. Mesoporous, Pristine and cyanamide functionalized carbon nitride were prepared according to a previously reported procedure. The photocatalytic experiments were performed using a blue LEDs based photoreactor (λ = 447 ± 20 nm, 1.03 W @ 700mA per LED) as a light source.[1] Scanning electron microscopy (SEM) images were collected on MIRA3 FEG-SEM (TESCAN) at an accelerating voltage of 5 kV. Xray photoelectron spectroscopy (XPS) was performed on a Thermo Fisher Scientific K-alpha+ spectrometer. Inductively coupled plasma-optical emission spectroscopy (ICP-OES) was carried-out at the Department of Geography, University of Cambridge with a Perkin-Elmer ICP-OES chemical analyzer. Products were purified by flash column chromatography on silica gel 60 (0.040-0.063 mm mesh) from Material Harvest. Thin layer chromatography (TLC) was carried out on aluminum Merck Kieselgel 60 F254 sheets, visualized by ultraviolet irradiation (254 and 365). ^{1}H and ^{13}C NMR spectroscopy were recorded on Bruker DPX 400 spectrometer at room temperature. Chemical shifts (δ) of ^{1}H and ^{13}C NMR spectra are given in ppm and the peaks were internally referenced against the residual solvent peak (note: $CDCl_3$ referenced δ 7.26 ppm for ^{1}H and 77.16 ppm for ^{13}C). NMR data were reported in the following form: Chemical shift, multiplicity, coupling constant and integration. Attenuated total reflection Fourier transform infrared (ATR-IR) spectra were recorded on a Nicolet iS50 spectrometer and reported in terms of frequency of absorption (cm^{-1}). Mass spectra were recorded on a Waters LCT premier Time of Flight mass spectrometer or Micromass Quadrupole-Time of Flight mass spectrometer. Reported mass values are within the error limits of 5 ppm.

ICP-OES sample preparation

Nickel loading in the materials were determined by ICP-OES after digestion of the material (<1 mg) in conc. HNO_3 (70%) (~1 mL) overnight and dilution to 10 mL with Milli-Q® water. The ICP data is shown in Table S1:

XPS sample preparation

XPS samples were prepared by drop casting 5 mg mL^{-1} ethanol suspension of the materials on clean FTO slides followed by drying at 60 °C. FTO slides were cleaned by (i) soaking in a solution containing 30% hydrogen peroxide, 35% ammonia solution and DI water in a vol:vol ratio of 1:1:5 respectively at 70 °C for 30 min, (ii) rinsing with milli-Q® water, and (iii) ultra-sonication in ethanol and acetone followed by drying under N_2 flow.

Preparation of mesoporous carbon nitride (mpg-CN_x)

Cyanamide (3 g) was heated at 50 ^oC until it melts and 40 wt.% dispersion of SiO_2 (7.5 g, Ludox SM) was added to it to form a homogeneous solution. The resultant transparent mixture was heated at 100 ^oC to form a white solid. The white solid was ground and transferred to alumina crucible and heated at a rate of 2.3 ^oC min^{-1} over 4 h to reach a temperature of 550 ^oC, and then kept at this temperature for a further 4 h. The resulting brown-yellow powder was treated with 4 M NH_4HF_2 for 24 h in order to remove the silica template. The powders were then washed three times with hot distilled water and twice with ethanol over suction filtration.

Preparation Ni deposited mpg- CN_x (Ni-mpg- CN_x)

mpg-CN_x (0.3 g) was suspended in 12 mL dry acetonitrile (12 mL), purged with N_2 and ultrasonicated for 10 min. Anhydrous $NiCl_2$ (50 mg) and anhydrous triethylamine (0.15 mL) was added to the suspension and the mixture was purged again with N_2 for 5 min. The suspension was stirred at room temperature for 30 min followed by heating under microware at 80 °C for 2 h. After cooling to room temperature, the resulting yellow solid was collected by

filtration sequentially washed with acetonitrile, hot water, boiling ethanol, boiling methanol, boiling acetone, and dried under vacuum.

General procedure for photocatalytic C-O coupling reactions

In a 10 mL borosilicate photoreactor vial charged with four glass beads (6 mm) Ni-mpg- CN_x (10 mg), aryl halide (0.4 mmol) and sodium hydroxide (0.48 mmol, 1.2 equiv.) were taken. The vial was crimped by a septum-aluminum cap and purged with N_2 atmosphere via "vacuum and N_2 refill" cycles (x3). Then, degassed alcohol (2 mL) as a solvent/substrate was added to vial with a syringe needle. The reaction mixture was then shaken rapidly using an orbital stirrer and irradiated with a blue LEDs based photoreactor (λ = 447 ± 20 nm, 1.03 W @ 700mA per LED) at 40 °C (Figure S15). The reaction was monitored by TLC. Upon completion of the reaction, the reaction mixture was purified by flash column chromatography.

General procedure for the NMR analysis during reaction optimization

Upon completion of the reaction, the reaction mixture was diluted with 1 mL of acetonitrile for better solubility of products and any side products. To the reaction mixture, 1,3,5-trimethoxy benzene (0.05 mmol) was added as an internal standard. Then, 200 µL of the resulting solution was transferred into a vial diluted it with 1 mL of deuterated acetonitrile. 700 µL of this solution was then submitted for NMR analysis. To get better resolution of the peaks in the NMR spectra 64 or 128 scans were performed. (Figure S16)

General procedure for the kinetic reactions

All catalytic reactions were conducted in a 10 mL septum-capped vial under vigorous stirring using an orbital stirrer and irradiating at 447 nm for 420 min under nitrogen atmosphere at 40 ºC, unless otherwise indicated. Catalytic photoreductions were performed in MeOH (2 mL) as reaction solvent mixture, 4-bromoacetophenone as substrate (200 mM, 0.4 mmol), Ni-mpg-CN_x (10 mg) and NaOH as base (1.2 equivalents, 240 mM). A 447 nm LED photoreactor was

employed as light source. 500 μl aliquots of the rection were taken along the reaction time and 1,3,5-Trimethoxybenzene (100 μl, 160 mM final concentration) was added as internal standard after the aliquot extraction and the reaction was quenched by adding 2 mL of DCM and 1 mL H_2O. The crude reaction mixtures were purified by extraction with DCM (1 x 2 mL), the organic layer was passed through a $MgSO_4$. The solvent from the resulting organic solution was evaporated using a rotary evaporator and subjected to ^{1}H-NMR analysis to determine the conversion of 4-bromoacetophenone and the yield of the desired coupling product respectively. All ^{1}H-NMR conversions and yields reported are an average of at least two runs.

General procedure for the recovery of the material after catalytic studies

After completion of the reaction, reaction mixture was transferred into tube and centrifuged at 7000 rpm for 10 mins. The recovered material was washed with water and acetone and centrifuged again. At the end of the process, more than 90% of the material can be recovered. (Figure S17)

External Quantum Efficiency (EQE) Measurement.

The external quantum efficiency (EQE) was determined using simulated solar light simulator (LOT LSN 254) equipped with a monochromator (LOT MSH 300). Sample was irradiated at 447 nm at a light intensity (I) of I ~ 6.40 mW cm^{-2} (exact intensity was checked after the experiment). For the calculation, we have assumed one photon is required to generate one coupling product although the Ni catalytic cycle is self-sustained. EQE was calculated using the following equation:

$$EQE\ (\%) = \frac{molecules\ of\ product}{number\ of\ photon\ absorbed} \times 100 = \frac{n_{product} N_A hc}{t_{irr} I \lambda A}$$

where, $n_{product}$ is number of moles of coupling product, N_A is Avogadro's number, h is Planck's constant, c is speed of light, t_{irr} is reaction time, I is the intensity of light, λ is the wavelength of incident light, and A is cross-sectional area of irradiation.

The reaction mixture of 4-bromoacetophenone (200 mM), NaOH (240 mM), Ni-mpg-CN_x (10 mg) and 1 mL methanol in 3 mL UV Cuvette was irradiated for 10 hours and repeated twice.

$$EQE\ (\%) = 2.26 \pm 0.1\ \%$$

2. Coupling products characterization

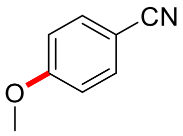

4-Methoxybenzonitrile

The reaction was completed in 5 hours and the desired product (white solid, 80% yield) was obtained by flash chromatography (10% ethyl acetate/hexane). ^1H NMR (400 MHz, Chloroform-*d*) δ 7.58 (d, J = 8.6 Hz, 2H), 6.95 (d, J = 8.6 Hz, 2H), 3.86 (s, 3H). ^{13}C NMR (101 MHz, $CDCl_3$) δ 162.97, 134.12, 119.36, 114.88, 104.10, 55.67. HRMS (ESI) calculated for $C_8H_8NO^+$ [$(M+H)^+$] 134.0601, found 134.0608.

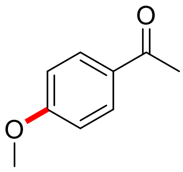

1-(4-Methoxyphenyl)ethan-1-one

The reaction was completed in 7 hours and the desired product (colorless oil, 86% yield) was obtained by flash chromatography (10% ethyl acetate/hexane). ^1H NMR (400 MHz, Chloroform-*d*) δ 7.94 (d, J = 8.6 Hz, 2H), 6.93 (d, J = 8.6 Hz, 2H), 3.87 (s, 3H), 2.55 (s, 3H). ^{13}C NMR (101 MHz, $CDCl_3$) δ 196.91, 163.63, 130.73, 130.51, 113.82, 55.60, 26.47. HRMS (ESI) calculated for $C_9H_{11}O_2^+$ [$(M+H)^+$] 151.0759, found 151.0763.

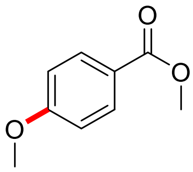

Methyl 4-methoxybenzoate

The reaction was completed in 8 hours and the desired product (white solid, 81% yield) was obtained by flash chromatography (10% ethyl acetate/hexane). ^1H NMR (400 MHz,

Chloroform-*d*) δ 7.99 (d, *J* = 8.6 Hz, 2H), 6.91 (d, *J* = 8.6 Hz, 2H), 3.88 (s, 3H), 3.85 (s, 3H). ^{13}C NMR (101 MHz, $CDCl_3$) δ 166.86, 163.34, 131.58, 122.63, 113.60, 55.40, 51.83. HRMS (ESI) calculated for $C_9H_{11}O_3^+$ [$(M+H)^+$] 167.0702, found 167.0701.

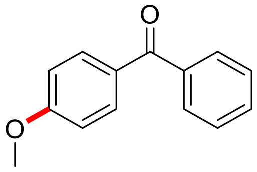

4-Methoxybenzophenone

The reaction was completed in 13 hours and the desired product (colorless oil, 87% yield) was obtained by flash chromatography (10% ethyl acetate/hexane). ^{1}H NMR (400 MHz, Chloroform-*d*) δ 7.83 (d, *J* = 8.7 Hz, 2H), 7.76 (d, *J* = 7.5 Hz, 2H), 7.55 (t, *J* = 7.5 Hz, 1H), 7.47 (t, *J* = 7.5 Hz, 2H), 6.96 (d, *J* = 8.7 Hz, 2H), 3.88 (s, 3H). ^{13}C NMR (101 MHz, $CDCl_3$) δ 195.64, 163.35, 138.43, 132.66, 131.99, 130.30, 129.83, 128.30, 113.68, 55.60. HRMS (ESI) calculated for $C_{14}H_{13}O_2^+$ [$(M+H)^+$] 213.0916, found 213.0916.

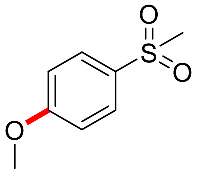

1-Methoxy-4-(methylsulfonyl)benzene

The reaction was completed in 7 hours and the desired product (white solid, 85% yield) was obtained by flash chromatography (15% ethyl acetate/hexane). ^{1}H NMR (400 MHz, Chloroform-*d*) δ 7.86 (d, *J* = 8.8 Hz, 1H), 7.01 (d, *J* = 8.8 Hz, 1H), 3.87 (s, 3H), 3.02 (s, 3H). ^{13}C NMR (101 MHz, $CDCl_3$) δ 163.81, 132.43, 129.65, 114.62, 55.82, 44.96. HRMS (ESI) calculated for $C_8H_{11}O_3S^+$ [$(M+H)^+$] 187.0423, found 187.0432.

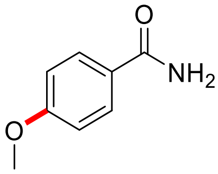

4-Methoxybenzamide

The reaction was completed in 23 hours and the desired product (white powder, 78% yield) was obtained by flash chromatography (20% ethyl acetate/hexane). ^{1}H NMR (400 MHz, Methanol-*d*) δ 7.84 (d, *J* = 8.7 Hz, 2H), 6.97 (d, *J* = 8.7 Hz, 2H), 3.84 (s, 3H). ^{13}C NMR (101 MHz, $CDCl_3$) δ 172.05, 164.17, 130.61, 126.95, 114.68, 55.91. Calculated for $C_8H_{10}NO_2^+$ [$(M+H)^+$] 152.0712, found 152.0714.

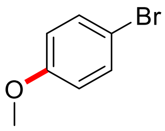

1-Bromo-4-methoxybenzene

The reaction was completed in 35 hours and the desired product (colorless oil, 78% yield) was obtained by flash chromatography (5% ethyl acetate/hexane). ^{1}H NMR (400 MHz, Chloroform-*d*) δ 7.38 (d, *J* = 8.9 Hz, 2H), 6.78 (d, *J* = 8.9 Hz, 2H), 3.78 (s, 3H). ^{13}C NMR (101 MHz, $CDCl_3$) δ 159.73, 132.39, 115.89, 112.98, 55.59. HRMS (ESI) calculated for $C_7H_8BrO^+$ [(M+H)$^+$] 186.9759, found 186.9774

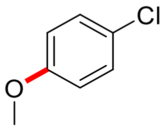

1-Chloro-4-methoxybenzene

The reaction was completed in 30 hours and the desired product NMR yield is 74% yield. ^{1}H NMR (400 MHz, Acetonitrile-*d*$_3$) δ 7.27 (d, *J* = 8.8 Hz, 2H), 6.89 (d, *J* = 8.8 Hz, 2H), 3.76 (s, 3H).

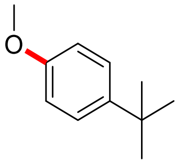

1-(tert-butyl)-4-methoxybenzene

The reaction was run for 72 hours and the desired product NMR yield is 42% with rest unreacted starting material. ^{1}H NMR (400 MHz, Acetonitrile-*d*$_3$) δ 7.44 (d, *J* = 8.0 Hz, 2H), 6.86 (d, *J* = 8.0 Hz, 2H), 3.77 (s, 3H), 1.29 (s, 9H).

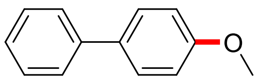

4-methoxy-1,1'-biphenyl

The reaction was completed in 64 hours and the desired product (white powder, 68% yield) was obtained by flash chromatography (3% ethyl acetate/hexane). ^{1}H NMR (400 MHz, Chloroform-*d*) δ 7.61-7.57 (m, 4H), 7.46 (t, *J* = 7.6 Hz, 2H), 7.35 (t, *J* = 7.6 Hz, 1H), 7.02 (d, *J* = 8.6 Hz, 2H), 3.88 (s, 3H). ^{13}C NMR (101 MHz, $CDCl_3$) δ 159.29, 140.95, 133.90, 128.84,

128.27, 126.85, 126.78, 114.34, 55.44. HRMS (ESI) calculated for $C_{13}H_{13}O^+$ $[(M+H)^+]$ 185.0966 , found 185.0971

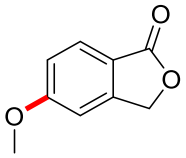

5-Methoxyisobenzofuran-1(3H)-one

The reaction was completed in 16 hours and the desired product (white powder, 64% yield) was obtained by flash chromatography (10% ethyl acetate/hexane). ^1H NMR (400 MHz, Chloroform-*d*) δ 7.82 (d, *J* = 8.5 Hz, 1H), 7.04 (d, *J* = 8.5 Hz, 1H), 6.92 (s, 1H), 5.25 (s, 2H), 3.90 (s, 3H). ^{13}C NMR (101 MHz, $CDCl_3$) δ 164.87, 149.51, 127.41, 118.26, 116.63, 106.15, 69.21, 55.99. HRMS (ESI) calculated for $C_9H_9O_3^+$ $[(M+H)^+]$ 165.0546, found 165.0551.

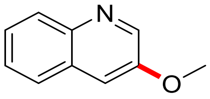

3-Methoxyquinoline

The reaction was completed in 14 h hours and the desired product (colorless oil, 66% yield) was obtained by flash chromatography (30 % ethyl acetate/hexane). ^1H NMR (400 MHz, Chloroform-*d*) δ 8.68 (d, *J* = 2.8 Hz, 1H), 8.05 (d, *J* = 8.2 Hz, 1H), 7.72 (d, *J* = 7.9 Hz, 1H), 7.59 – 7.47 (m, 2H), 7.37 (d, *J* = 2.8 Hz, 1H), 3.94 (s, 3H). ^{13}C NMR (101 MHz, $CDCl_3$) δ 153.23, 144.71, 143.67, 129.33, 128.94, 127.20, 126.80, 126.78, 112.33, 55.59. HRMS (ESI) calculated for $C_{10}H_{10}NO^+$ $[(M+H)^+]$ 160.0757, found 160.0755.

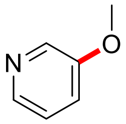

3-Methoxypyridine

The reaction was completed in 14 hours and the desired product (Colorless liquid, 74% yield) was obtained by flash chromatography (20% ethyl acetate/hexane). ^1H NMR (400 MHz, Chloroform-*d*) δ 8.32 (d, *J* = 2.7 Hz, 1H), 8.27 – 8.19 (m, 1H), 7.24 – 7.14 (m, 2H), 3.86 (s, 3H). ^{13}C NMR (101 MHz, $CDCl_3$) δ 156.75, 142.10, 137.58, 123.79, 120.48, 55.49.
HRMS (ESI) calculated for $C_6H_7NO^+$ $[(M+H)^+]$ 110.0600, found 110.0600.

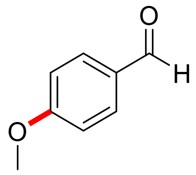

4-Methoxybenzaldehyde

The reaction was completed in 7 hours and the desired product (colorless liquid, 92% yield) was obtained by flash chromatography (10% ethyl acetate/hexane). ^{1}H NMR (400 MHz, Chloroform-*d*) δ 9.87 (s, 1H), 7.82 (d, *J* = 8.3 Hz, 2H), 6.99 (d, *J* = 8.3 Hz, 2H), 3.87 (s, 3H). ^{13}C NMR (101 MHz, $CDCl_3$) δ 190.91, 164.73, 132.08, 130.07, 114.42, 55.67.
HRMS (ESI) calculated for $C_8H_9O_2^+$ [(M+H)$^+$] 137.0603, found 137.0604.

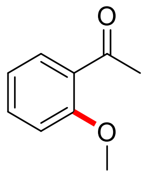

1-(2-methoxyphenyl)ethan-1-one

The reaction was completed in 12 hours and the desired product (colorless liquid, 60% yield) was obtained by flash chromatography (5% ethyl acetate/hexane). ^{1}H NMR (400 MHz, Chloroform-*d*) δ 7.73 (d, *J* = 8.0 Hz, 1H), 7.48 – 7.44 (m, 1H), 7.07 – 6.89 (m, 2H), 3.91 (s, 3H), 2.61 (s, 3H). ^{13}C NMR (101 MHz, $CDCl_3$) δ 200.00, 159.04, 133.76, 130.48, 128.44, 120.68, 111.70, 55.60, 31.94. HRMS (ESI) calculated for $C_9H_{11}O_2^+$ [(M+H)$^+$] 151.0759, found 151.0763.

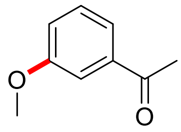

1-(3-methoxyphenyl)ethan-1-one

The reaction was completed in 36 hours and the desired product (colorless liquid, 84% yield) was obtained by flash chromatography (5% ethyl acetate/hexane). ^{1}H NMR (400 MHz, Chloroform-*d*) δ 7.53 (d, *J* = 7.8 Hz, 1H), 7.48 (s, 1H), 7.37 (dd, *J* = 7.8 Hz, *J* = 8.2 Hz, 1H), 7.11 (d, *J* = 8.2 Hz, 1H), 3.86 (s, 3H), 2.59 (s, 3H). ^{13}C NMR (101 MHz, $CDCl_3$) δ 198.10, 159.97, 138.69, 129.69, 121.26, 119.76, 112.51, 55.58, 26.85. $C_9H_{11}O_2^+$ [(M+H)$^+$] 151.0759, found 151.0759.

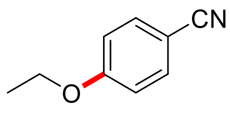

4-ethoxybenzonitrile

The reaction was completed in 18 hours and the desired product (white powder, 75% yield) was obtained by flash chromatography (10% ethyl acetate/hexane). ^{1}H NMR (400 MHz,

Chloroform-*d*) δ 7.57 (d, *J* = 7.6 Hz, 2H), 6.92 (d, *J* = 7.6 Hz, 2H), 4.07 (q, *J* = 6.9 Hz, 2H), 1.43 (t, *J* = 6.9 Hz, 3H). ^{13}C NMR (101 MHz, $CDCl_3$) δ 162.40, 134.09, 119.42, 115.29, 103.85, 64.06, 14.68. HRMS (ESI) calculated for $C_9H_{10}NO^+$ $[(M+H)^+]$ 148.0762, found 148.0765.

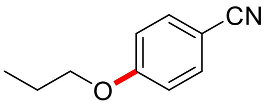

4-propoxybenzonitrile

The reaction was completed in 30 hours and the desired product (colorless liquid, 68% yield) was obtained by flash chromatography (5% ethyl acetate/hexane). ^{1}H NMR (400 MHz, Chloroform-*d*) δ 7.58 (d, *J* = 8.4 Hz, 2H), 6.95 (d, *J* = 8.4 Hz, 2H), 3.98 (t, *J* = 6.6 Hz, 2H), 1.87– 1.82 (m, 2H), 1.06 (t, *J* = 7.4 Hz, 3H). ^{13}C NMR (101 MHz, $CDCl_3$) δ 162.58, 134.06, 119.43, 115.30, 103.77, 69.98, 22.46, 10.51 HRMS (ESI) calculated for $C_{10}H_{12}NO^+$ $[(M+H)^+]$ 162.0919, found 162.0922.

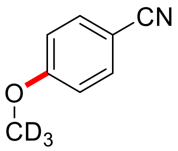

4-(methoxy-d$_3$)benzonitrile

The reaction was completed in 7 hours and the desired product (white powder, 78% yield) was obtained by flash chromatography (5% ethyl acetate/hexane). ^{1}H NMR (400 MHz, Chloroform-*d*) δ 7.59 (d, *J* = 8.4 Hz, 2H), 6.95 (d, *J* = 8.4 Hz, 2H). ^{13}C NMR (101 MHz, $CDCl_3$) δ 162.96, 134.08, 119.32, 114.85, 104.05. HRMS (ESI) calculated for $C_8H_5D_3NO^+$ $[(M+H)^+]$ 137.07887, found 137.0844 (due to Deuterium the mass error is 40 ppm).

3. Kinetic studies

The activation parameters of the C-O coupling reaction by the dual Ni-mpg- CN_x photocatalytic system were obtained through kinetic analysis of the formation of 4-methoxy acetophenone (**3**) during initial reaction times in a range of temperatures between 293 K and 323 K (Figure S11). The reaction rate at a given temperature was approximated as the initial rate of formation of **3** quantified by means of ^{1}H-NMR. Then, the experimental data obtained from the kinetics

experiments (Table S5) was fitted to the Eyring equation (Eq. 1) and represented in Figure S12:[2,3]

$$\ln\frac{k}{T} = -\frac{\Delta H^{\ddagger}}{R}\frac{1}{T} + \ln\frac{k k_B}{h} + \frac{\Delta S^{\ddagger}}{R} \qquad \text{(Eq. 1)}$$

where,

k = reaction rate constant

T = absolute temperature

$\Delta H^{\ddagger}$ = enthalpy of activation

R = gas constant

k_B = Boltzmann constant

h = Planck's constant

$\Delta S^{\ddagger}$= entropy of activation

After the analysis of the linear fit in Figure S13 (slope of -1230.7 and an intercept of -12.0212), the calculation of the experimental activation parameters was performed according to the following expressions:

$$\Delta H^{\ddagger} = -slope \cdot R \qquad \text{(Eq. 2)}$$

$$\Delta S^{\ddagger} = R \cdot intercept - R \cdot \ln\frac{k_B}{h} \qquad \text{(Eq. 3)}$$

$$\Delta G^{\ddagger} = \Delta H^{\ddagger} - T \cdot \Delta S^{\ddagger} \qquad \text{(Eq. 4)}$$

The results obtained from the Eyring analysis are summarized in Table S6.

Since the value of $\Delta S^{\ddagger}$ is related to the rate determining step in a reaction.[4] The negative value of it suggest that entropy decreases upon achieving the transition state, which often indicates an associative mechanism in which two reaction partners form a single activated complex, this is agreement with an associative mechanism and the first order observed regarding the substrate.

4. Computational studies

DFT calculations have been performed with the Gaussian09 software package.[5] First, geometry optimizations and frequency calculations of the ground state structure of a simplified model for the Ni-mpg-CN_x system have been performed at the B3LYP/6-31G* level of theory (see Scheme S1a).[6–8] Solvent effects (MeOH) are considered with the polarizable continuum model PCM-SMD of Truhlar and coworkers. [9–12] Additionally, the energy of the geometry optimized molecules was refined by single point calculation with the 6-311+G** basis set[13–15] for all atoms.

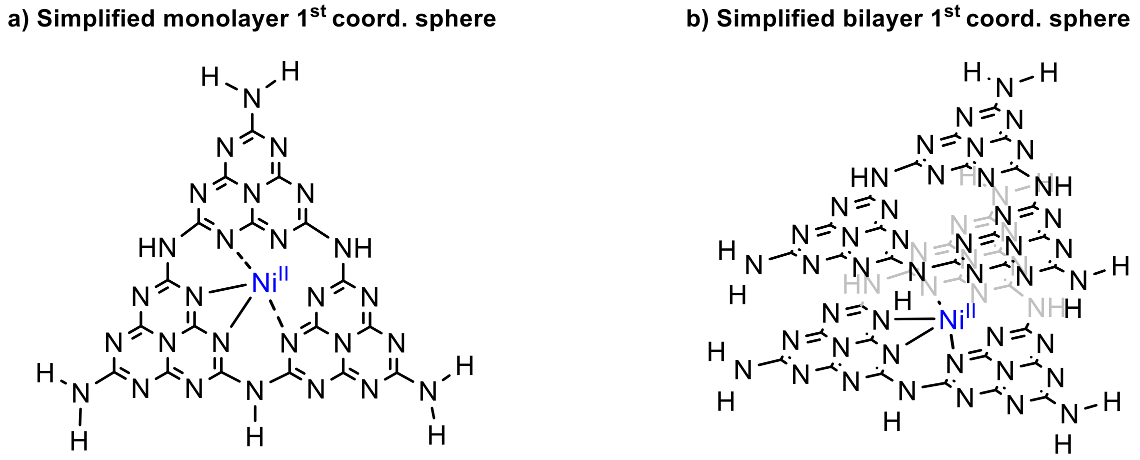

Scheme S1. Simplified model systems used for the computational studies.

Standard reduction potentials (E°) have been evaluated through the Nernst equation in standard state conditions using the Standard Hydrogen Electrode (SHE) as reference following the equation 5:

$$E^o = -\frac{\Delta G^o - \Delta G^o_{SHE}}{nF} \quad \text{(Eq. 5)}$$

where ΔG^o is the free energy change associated with reduction at standard conditions, n is the number of electrons involved in the redox reaction, F is the Faraday constant and ΔG^o_{NHE} is the free energy change associated with the reduction of a proton (-4.28 eV).[16]

Finally, a 2-layer ONIOM (own n-layered integrated molecular orbital and molecular mechanics) method developed by Morokuma and co-workers[12,17,18] has been used to get a better picture of the Ni coordination within the heterogeneous catalytic material at a very simple level of theory while having a compromise between a reliable geometry Optimisation and computational cost. The previously optimized Ni(II)-mpg-CNx and Ni(I)-mpg-CNx structures at a B3LYP/6-31G* level of theory were further used in combination with a second layer of the heterogeneous CN_x material for the 2-layer ONIOM calculations (see scheme 1 b). Additionally, the energy of the geometry optimized molecules at the ONIOM B3LYP/6-31G* level was refined by single point calculation with the 6-311+G** basis set[13–15] for all atoms. The optimized structures for Ni^{II}-mpg-CN_x and Ni^{I}-mpg-CN_x show the same distorted square planar type coordination of the Ni centre within the mpg-CN_x layer (Figure S18).

All energies given in this work are referred to Gibbs energies G in kcal·mol^{-1}, unless otherwise noted. The nature of the stationary points was established by frequency calculations in the solvent-phase, in which minima have no imaginary frequencies.

Redox potentials

The possibility to have a first reduction of the Ni(II) centre in Ni-mpg-CNx to Ni(I) after photoinduced electron transfer (PET) from the mpg-CN_x matrix to the Ni site and then a subsequent reduction to Ni(0) by intramolecular PET was evaluated:

$$\mathbf{Ni^{II}}\text{-mpg-}CN_x \xrightarrow{E^o = 0.33\ V} \mathbf{Ni^{I}}\text{-mpg-}CN_x \xrightarrow{E^o = -1.51\ V} \mathbf{Ni^{0}}\text{-mpg-}CN_x$$

Considering that the potential of the CNx conduction band is - 0.5 V vs. SHE the reduction of the Ni(II) centre to Ni(I) within the mpg-CN_x matrix is possible (0.33 V *vs* SHE), but not a second reduction of the Ni(I) to Ni(0) (–1.51 V $_{vs}$ SHE).

Calculated energy profile and proposed catalytic cycle

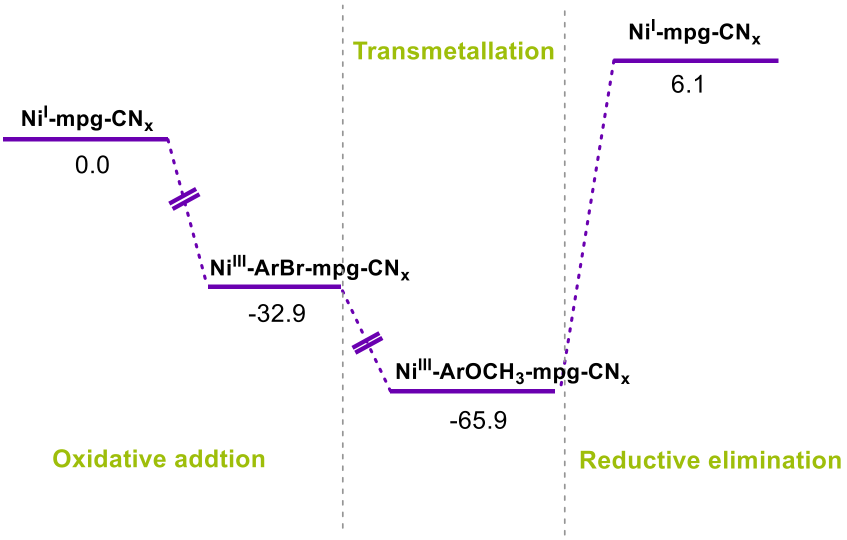

Scheme S2. Energy profile for the C-O coupling catalytic cycle starting from Ni-mpg-CNx.

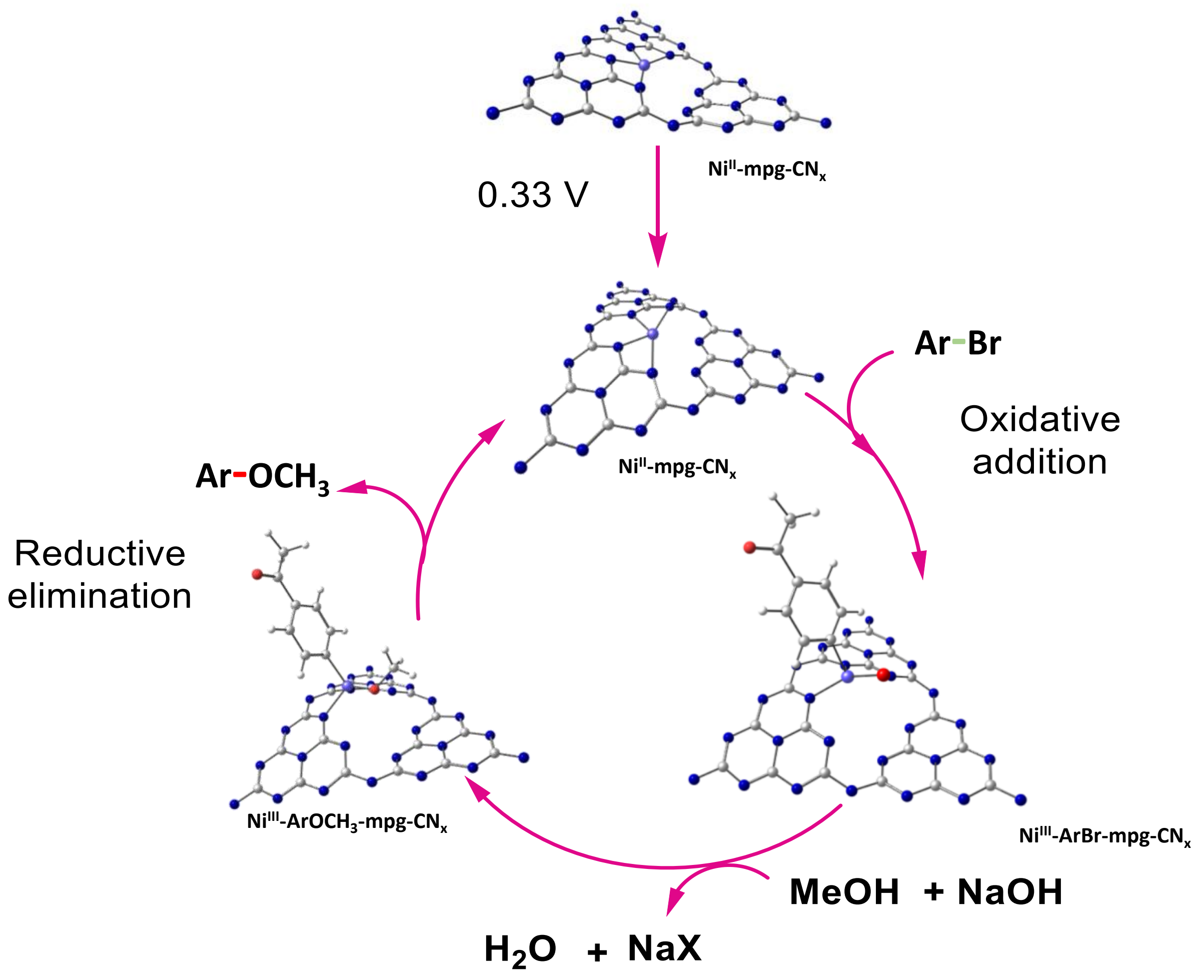

Scheme S3. Summary of the computed C-O coupling catalytic cycle for Ni-mpg-CNx with theoretical redox values. Values represent the redox potential for the ET values [V *vs* SHE] at 298 K. Subscripts indicate the spin multiplicity. Energies represent elementary steps and they are given in kcal·mol^{-1}.

5. Tables

Table S1. ICP-OES data for different Ni deposited carbon nitrides.

Sample	Ni wt%
Ni-mpg-CN_x (batch 1)	5.427
Ni- CN_x	3.882
Ni-^{NCN}CNx	2.600
Ni-mpg-CN_x (batch 2)	4.002
Ni-mpg-CN_x (batch 3)	2.997
Recycle 1st Ni-mpg-CN_x	2.638
Recycle 3rd Ni-mpg-CN_x	2.240
Recycle 4th Ni-mpg-CN_x	2.238
$NiCl_2$ (1wt%) + mpg-CN_x post-catalysis	0.757
$NiCl_2$ (2wt%) + mpg-CN_x post-catalysis	1.267
$NiCl_2$ (3wt%) + mpg-CN_x post-catalysis	2.001
$NiCl_2$ (5wt%) + mpg-CN_x post-catalysis	3.348

Note: Ni-mpg-CN_x batch 1 and 2 was used for Optimization and substrate scope and batch 3 was used for kinetic and recycling experiment. All the batches have similar activity for the cross-coupling reaction.

Table S2. Screening of different solvents.

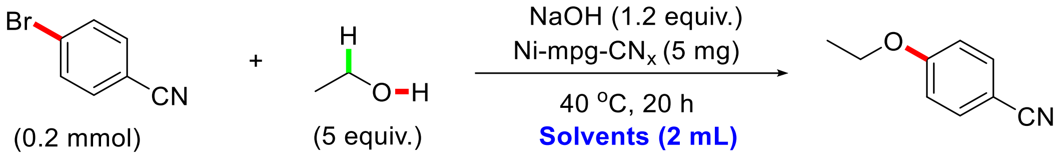

Entry	Solvent	Product[a]
1	Acetonitrile	traces
2	Dimethoxy ethane	traces
3	Acetone	0
4	*N, N*- Dimethylacetamide (DMA)	18%
5[b]	DMA	25%
6[c]	DMA	20%
7[d]	DMA	12%
8	Tetrahydrofuran	traces
9	Toluene	0
10	Ethyl acetate	0
11	Dimethyl sulfoxide (DMSO)	26%
12[b]	DMSO	27%
13[c]	DMSO	16%
14[d]	DMSO	27%
15	*N, N*- dimethylformamide	15%

Reaction conditions: 4-bromobenzonitrile = 100 mM, Ni-mpg- CN_x = 5 mg, [base] = 120 mM (1.2 equiv.), ethanol = 500 mM (5 equiv.) in different solvent solution (2 mL), 20 hours irradiation at λ = 447 ± 20 nm and 40 °C under N_2 [a] Determined by ^{1}H-NMR using 1,3,5-trimethoxybenzene (50 µmol) as internal standard. [b] Ethanol = 1000 M (10 equiv); [c] Ethanol = 2 M (20 equiv.); [d] Ethanol = 500 mM (5 equiv.), triethyl amine = 200 mM (2 equiv).

Table S3. Screening of different bases.

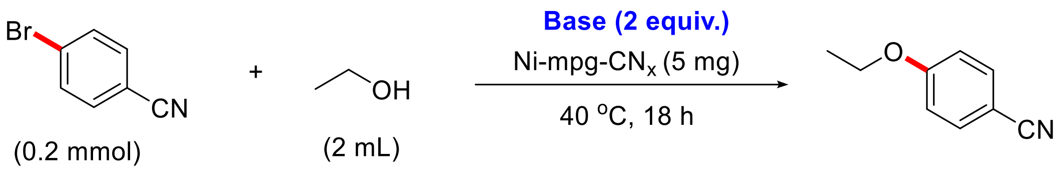

Entry	Base	Product[a]
1.	K_3PO_4	traces
2.	K_2CO_3	32%
3.	NaOH	75%
4.	LiOH	42%
5.	TBAOH	19%
6.	DABCO	3%
7.	DBU	traces
8.	Triethyl amine	8%
9.	N, N-Diisopropylethylamine	6%
10.	Diisopropylamine	10%
11.	N-*tert*-butylisopropylamine	6%
12.	Tetramethylpiperidine	8%

Reaction conditions: 4-bromobenzonitrile = 100 mM, Ni-mpg- CN_x = 5 mg, [base] = 100 mM in ethanol solution (2 mL), 18 hours irradiation at $\lambda = 447 \pm 20$ nm and 40 °C under N_2 [a] Determined by ^{1}H-NMR using 1,3,5-trimethoxybenzene (50 µmol) as internal standard. Abbreviations: TBAOH is tetrabutylammonium hydroxide; DABCO is 1,4-diazabicyclo[2.2.2]octane; DBU is 1,8-Diazabicyclo[5.4.0]undec-7-ene.

Table S4. Optimisation of NaOH loading.

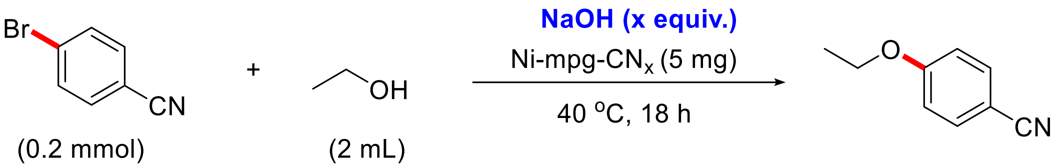

Entry	Loading (equiv.)	Product[a]
1.	0.5	33%
2.	1	68%
3.	1.2	82%
4.	2	75%

Reaction conditions: 4-bromobenzonitrile = 100 mM, Ni-mpg- CN_x = 5 mg, [NaOH] = x equivalence in ethanol solution (2 mL), 18 hours irradiation at λ = 447 ± 20 nm and 40 °C under N_2. [a] Determined by ^{1}H-NMR using 1,3,5-trimethoxybenzene (50 µmol) as internal standard.

Table S5. Experimental values employed in the Eyring analysis.

T (K)	T^{-1} (K^{-1})	k ($M\cdot s^{-1}$)	ln($k\cdot T^{-1}$)
293	0.0034	2.67E-05	–16.21
303	0.0033	3.17E-05	–16.07
313	0.0032	3.67E-05	–15.96
323	0.0031	4.67E-05	–15.75

Table S6. Summary of the results obtained from the Eyring plot.

Activation parameter	Value	Units
$\Delta H^{\ddagger}$	2.81 ± 0.31	$kcal\cdot mol^{-1}$
$\Delta S^{\ddagger}$	–70.0 ± 1	$cal\cdot mol^{-1}\cdot K^{-1}$
$\Delta G^{\ddagger}$	24.68 ± 0.31	$kcal\cdot mol^{-1}$

6. Figures

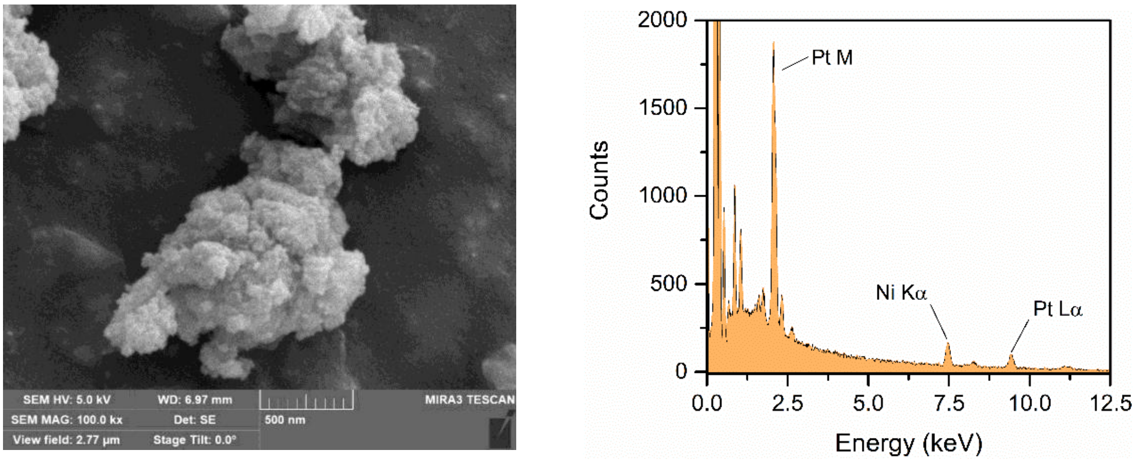

Figure S1: SEM image of Ni-mpg- CN_x and corresponding EDS spectrum of the region shown in yellow box (Pt peaks are caused by sputtering).

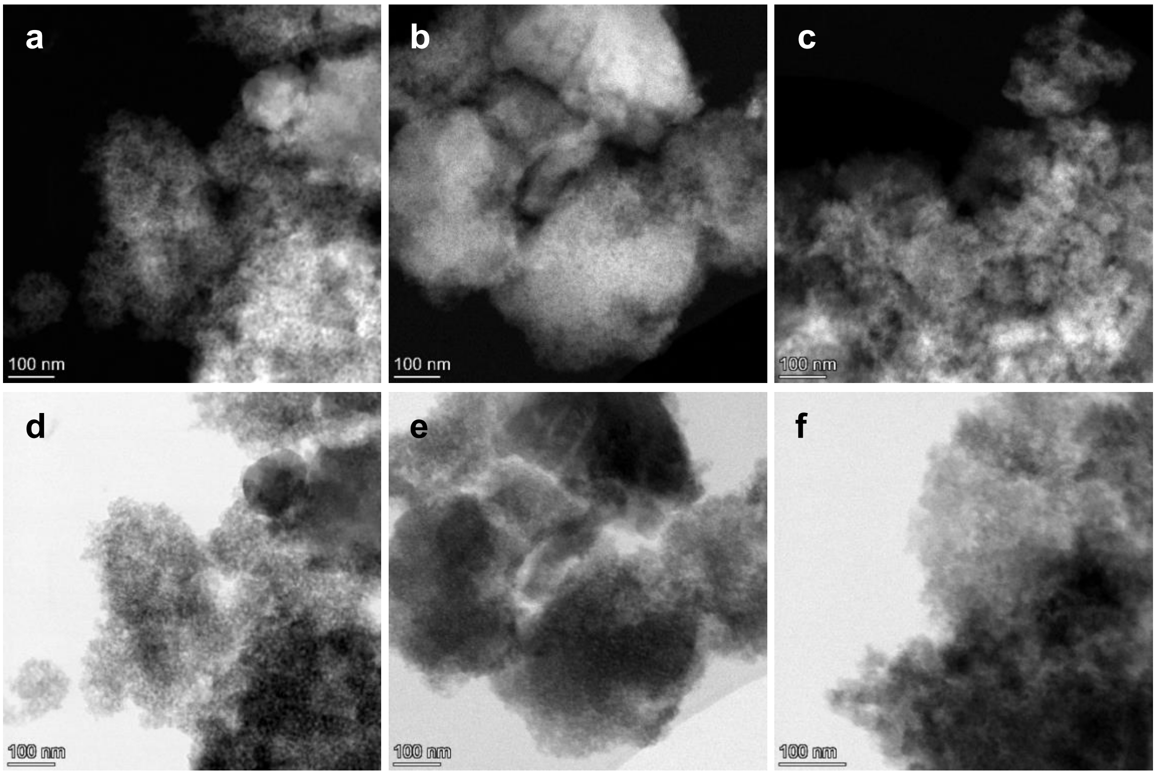

Figure S2: High-angle annular dark-field (HAADF-STEM, top panel, a-c) and bright-field (BF-STEM, bottom panel, d-f). Images of Ni-mpg- CN_x (a and d), same material after photocatalysis (b and e), and the material obtained after performing catalysis with $NiCl_2$(salt)/mpg- CN_x (c and f).

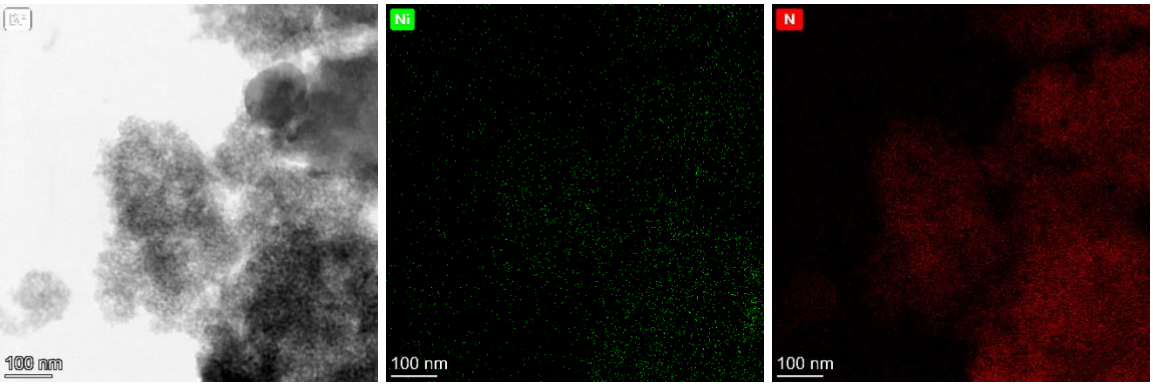

Figure S3: BF-STEM image (left) and EDS mapping (Ni and N, middle and right images respectively) of Ni-mpg- CN_x.

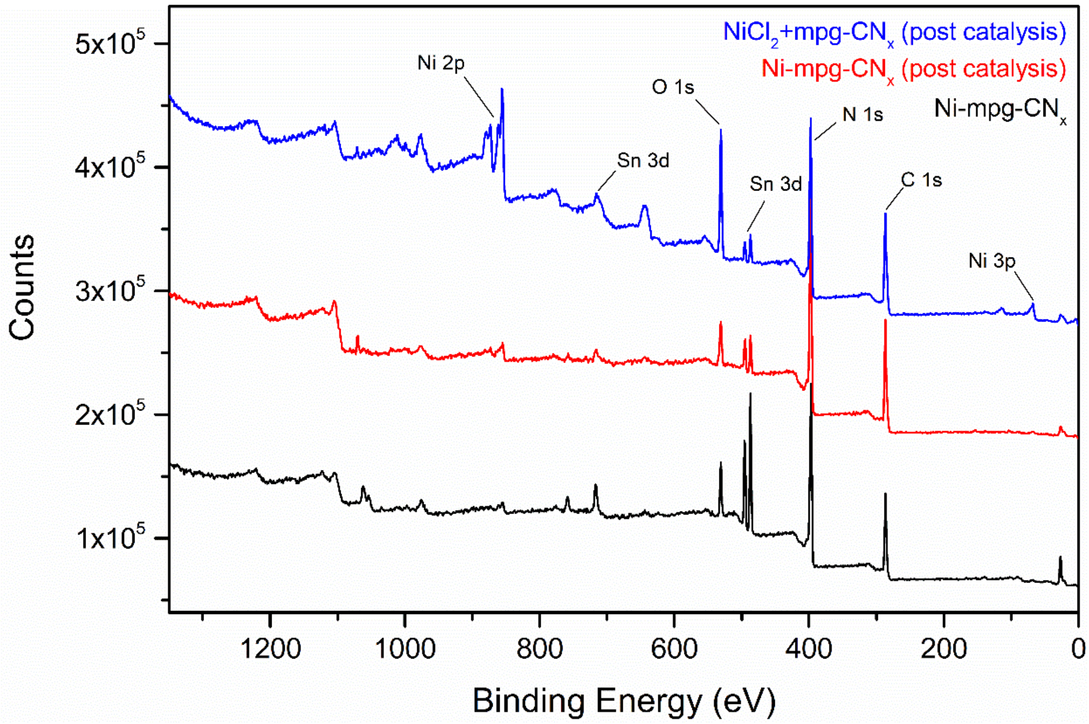

Figure S4: Survey XPS spectra of Ni-mpg- CN_x (as synthesized, black trace), same material after photocatalysis (red trace), and the material obtained after performing catalysis with $NiCl_2$ (salt)/mpg- CN_x (blue trace).

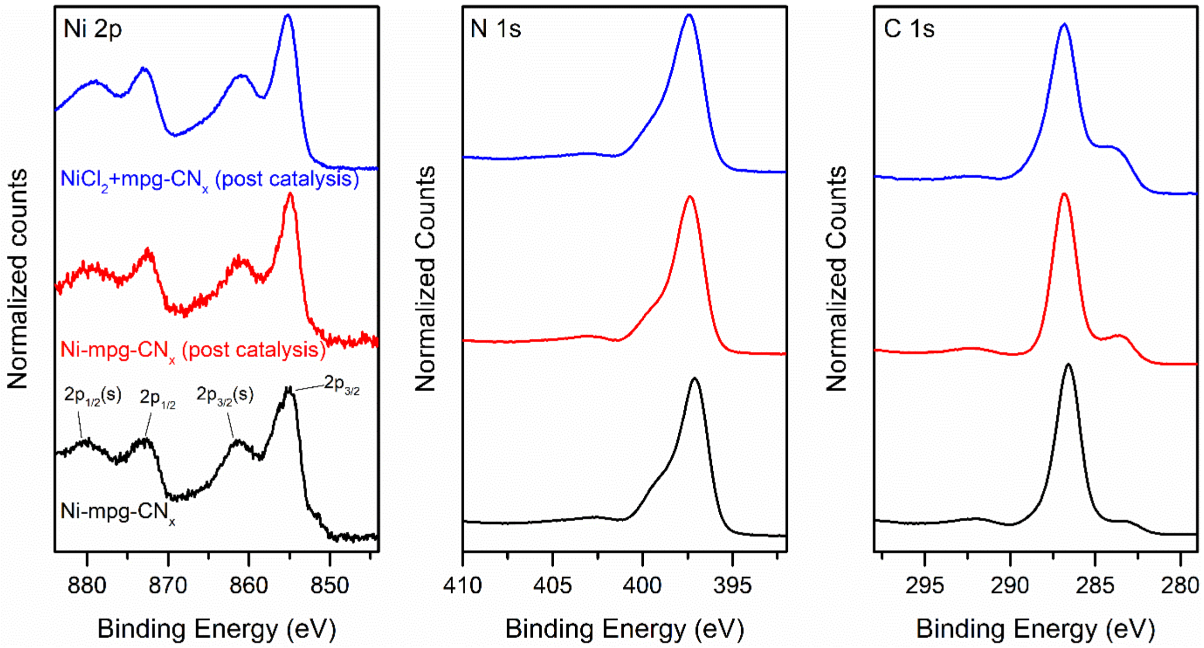

Figure S5: High resolution Ni 2p, N 1s and C 1s XPS spectra of Ni-mpg- CN_x (as synthesized, black trace), same material after photocatalysis (red trace), and the material obtained after performing catalysis with $NiCl_2$ (salt)/mpg- CN_x (blue trace).

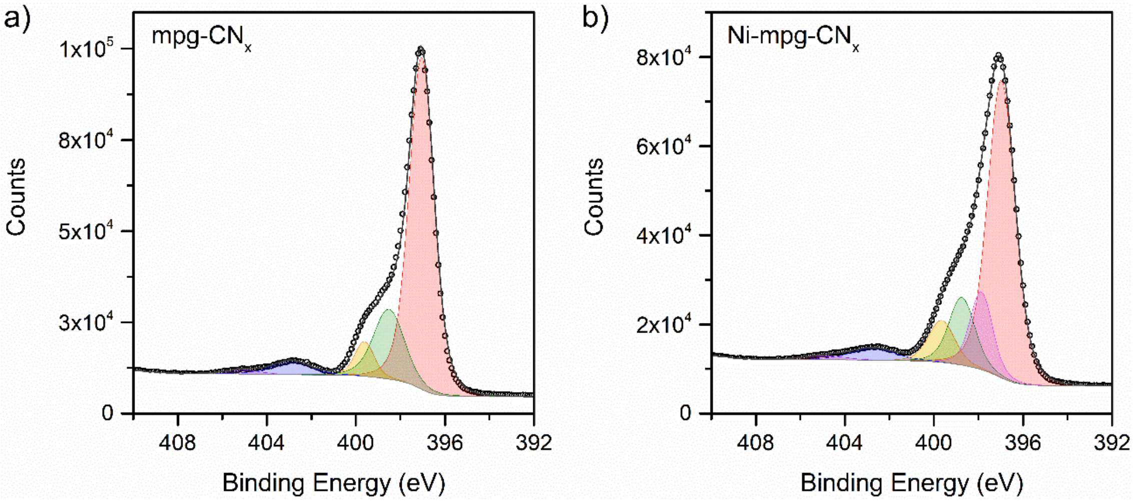

Figure S6: High resolution N1s XPS spectra of mpg-CN_x and Ni-mpg-CN_x. The raw data (open circles) was fitted to the components using casaXPS software. The dark grey solid line corresponds to the overall fitted result. Deconvolution of the main XPS peak for unmodified mpg-CN_x shows three peaks at 397.0 eV (red), 398.6 eV (green) and 399.6 eV (yellow). Ni-mpg-CN_x shows an additional peak ta 397.9 eV which can be attributed to pyridinic N coordinated to Ni^{2+}.

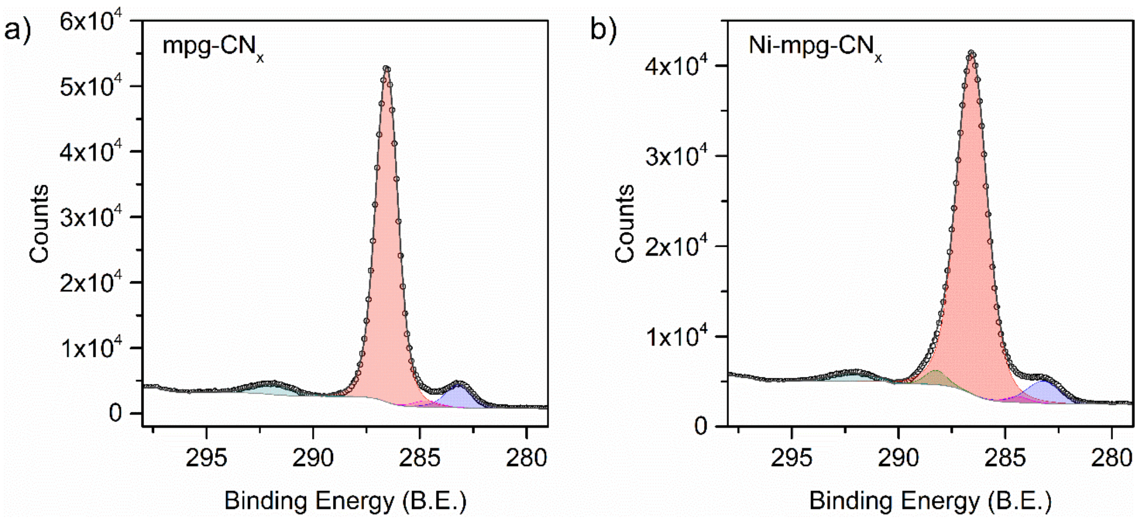

Figure S7: High resolution C1s XPS spectra of mpg-CN$_x$ and Ni-mpg-CN$_x$. The raw data (open circles) was fitted to the components using casaXPS software. The dark grey solid line corresponds to the overall fitted result.

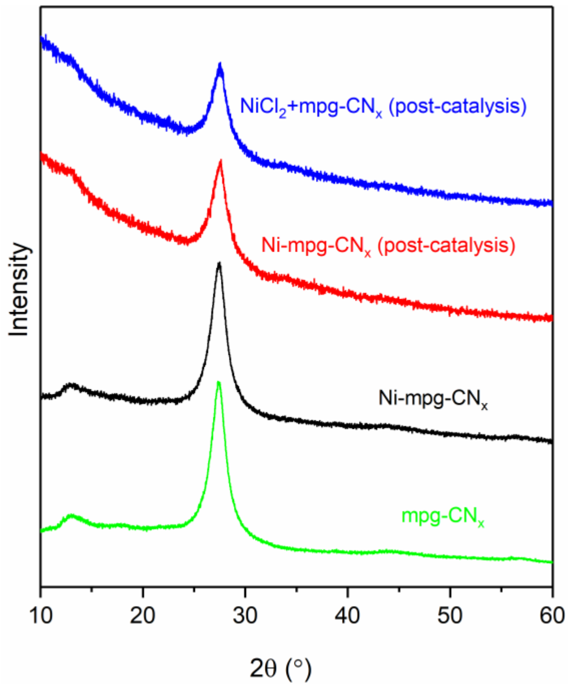

Figure S8: Powder X-ray diffraction patterns of mpg- CN$_x$ (green), Ni- mpg- CN$_x$ (black) same material after photocatalysis (red trace), and the material obtained after performing catalysis with $NiCl_2$ (salt)/mpg- CN$_x$ (blue trace).

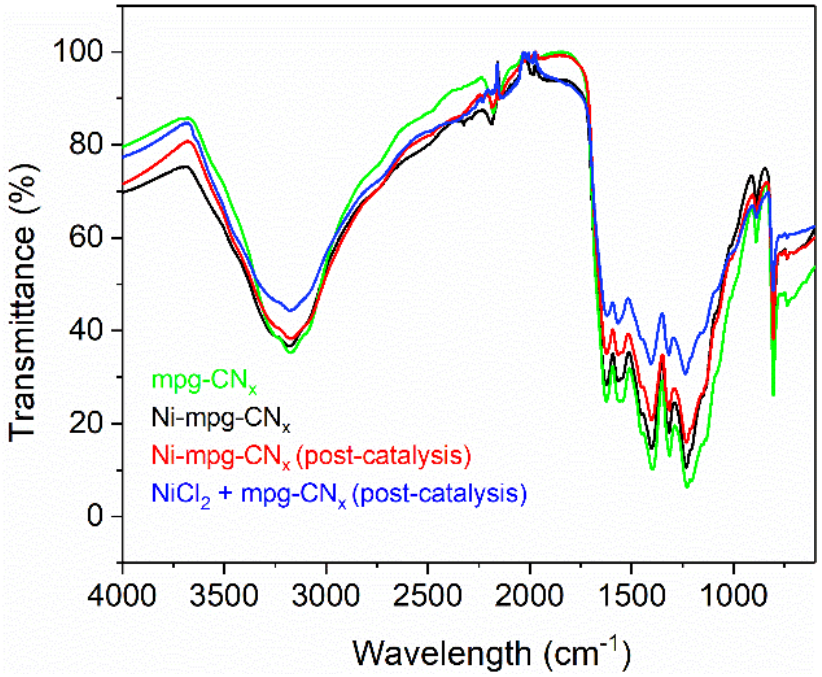

Figure S9: ATR-IR spectrum of mpg- CN_x (green), Ni- mpg- CN_x (black) same material after photocatalysis (red trace), and the material obtained after performing catalysis with $NiCl_2$ (salt)/mpg- CN_x (blue trace).

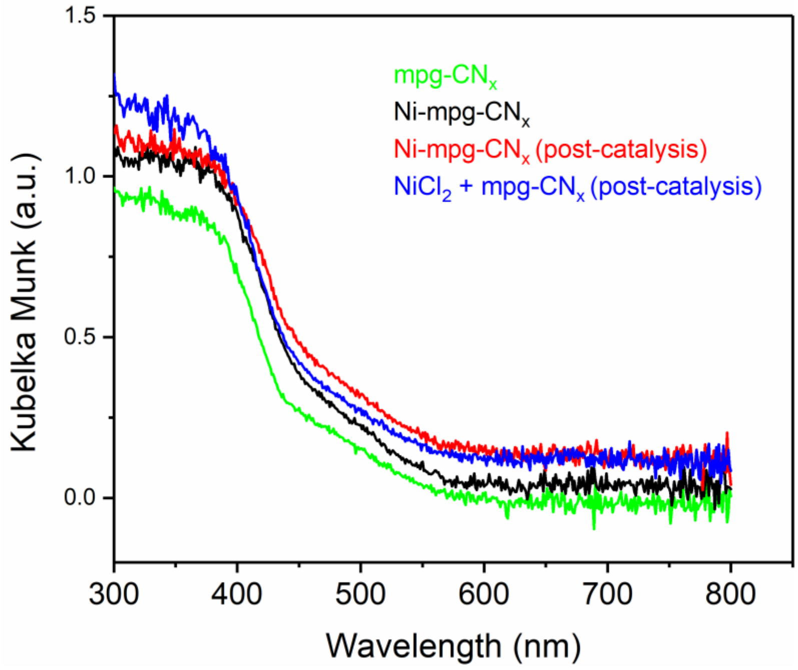

Figure S10: UV-vis spectra of mpg- CN_x (green), Ni- mpg- CN_x (black) same material after photocatalysis (red trace), and the material obtained after performing catalysis with $NiCl_2$ (salt)/mpg- CN_x (blue trace).

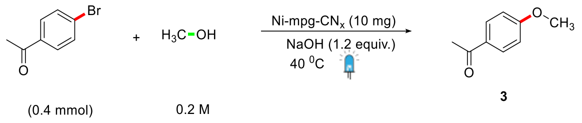

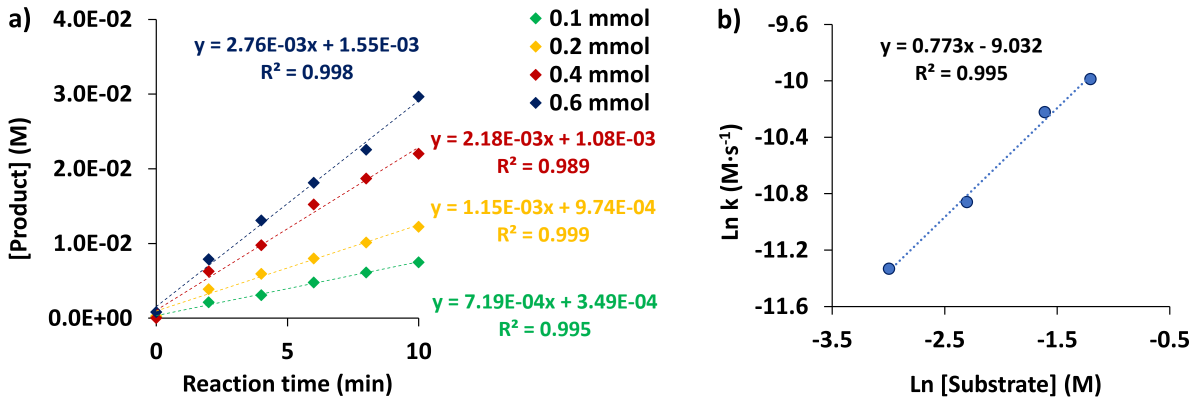

Figure S11: a) Kinetic initial rate traces of the C-O coupling reaction between 4-bromoacetophenone and methanol at 40 °C during 447 nm irradiation along 10 min reaction time. b) Fitting to extract the reaction order regarding the substrate. Catalytic conditions: [**3**] = 0.05 M (green), 0.1 M (yellow), 0.2 M (red) and 0.3 M (blue), Ni-mpg-CN_x = 10 mg, [NaOH]= 240 mM in MeOH solution (2 mL), 10 min irradiation at λ = 447 ± 20 nm and 40 °C under N_2. The rate order was found to be 0.77 ± 0.04 with an intercept of -9.032 ± 0.086.

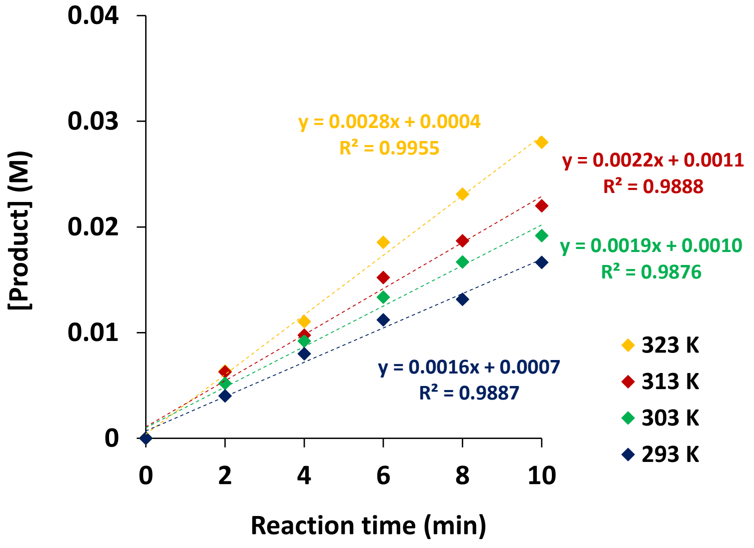

Figure S12: Kinetic traces of the light-driven C-O coupling between **3** and methanol over the initial reaction time (first 8 min of reaction) at 293 K (blue), 303 K (green), 313 K (red) and 323 K (yellow) as well as the corresponding linear fit. Time 0 (where 0 % yield of product and 0 % conversion of substrate are observed) was also included into the linear fit.

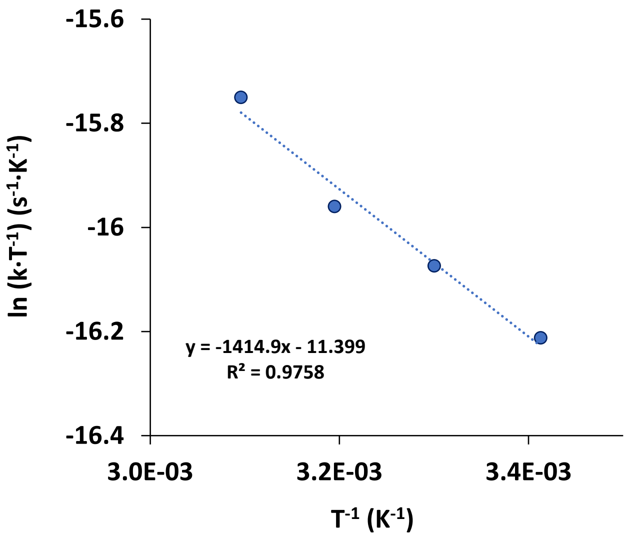

Figure S13: Eyring plot and linear fit of the kinetic data in Table S5.

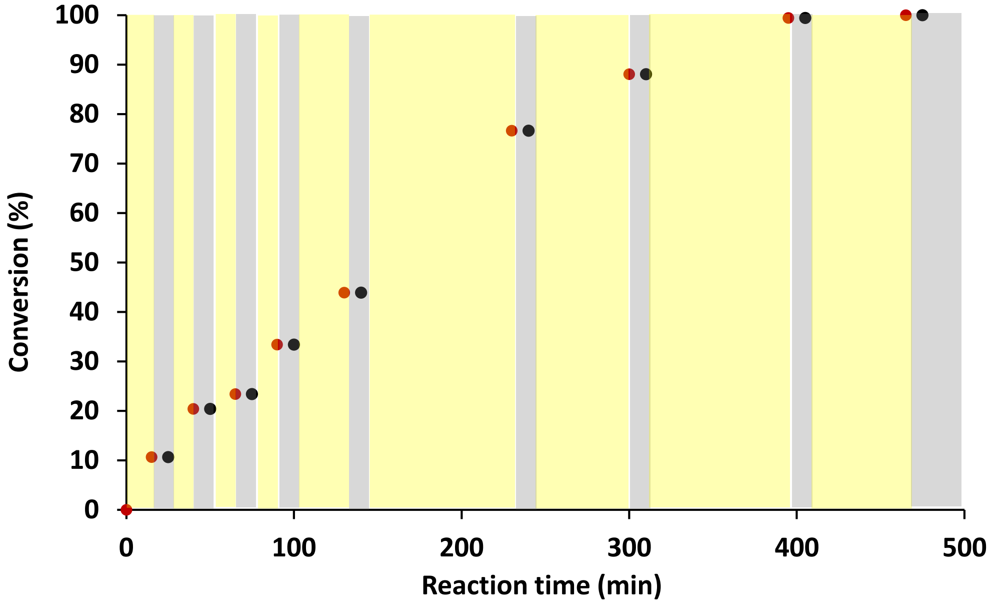

Figure S14: Light-dark monitoring of C–O coupling reaction between 4-bromoacetophenone with MeOH. Catalytic conditions: 4-bromoacetophenone= 200 mM, Ni-mpg-CN_x = 10 mg, [NaOH] = 240 mM in MeOH solution (2 mL), 420 min total irradiation at $\lambda = 447 \pm 20$ nm and 40 ºC under N_2 (Dark time was 10 mins).

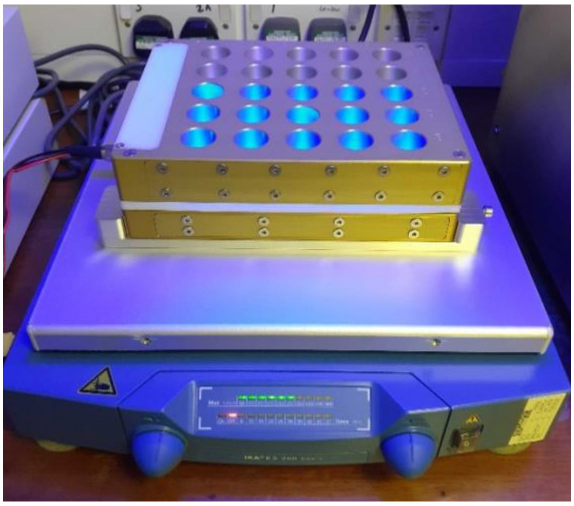

Figure S15: Representation of the photoreactor and setup used for the photocatalytic experiments.

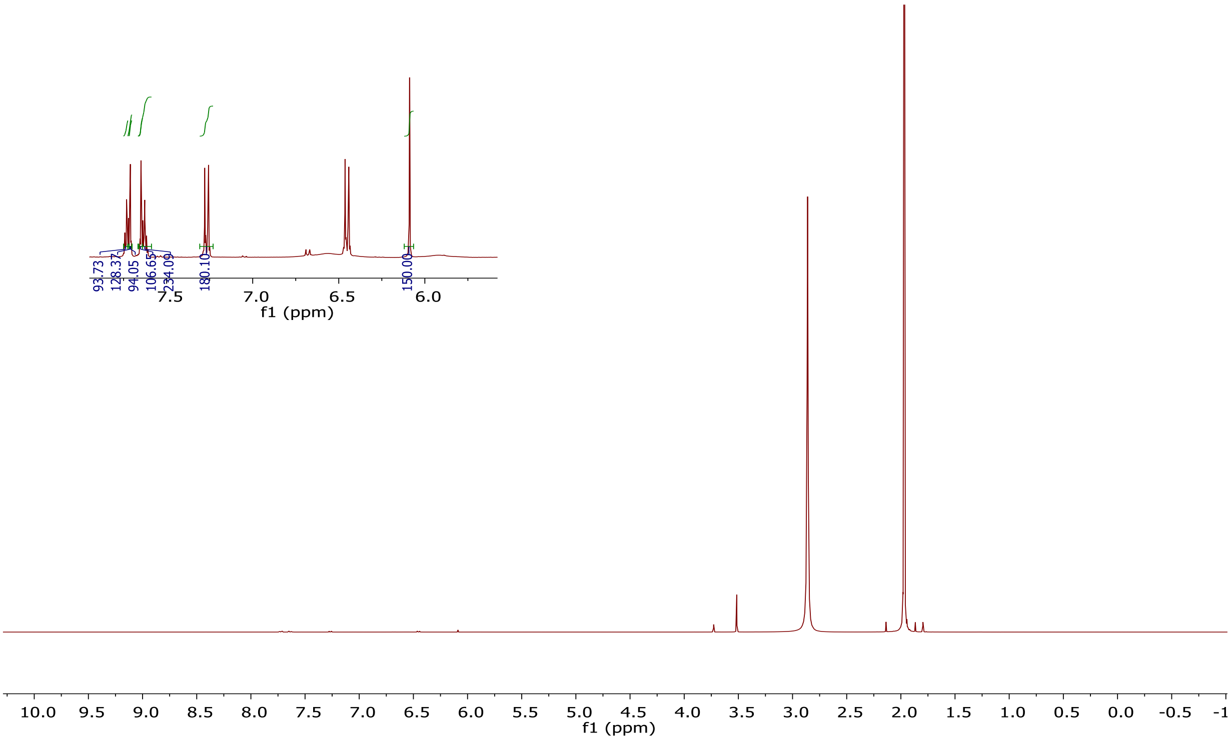

Figure S16: Representation of NMR spectra used for yield determination.

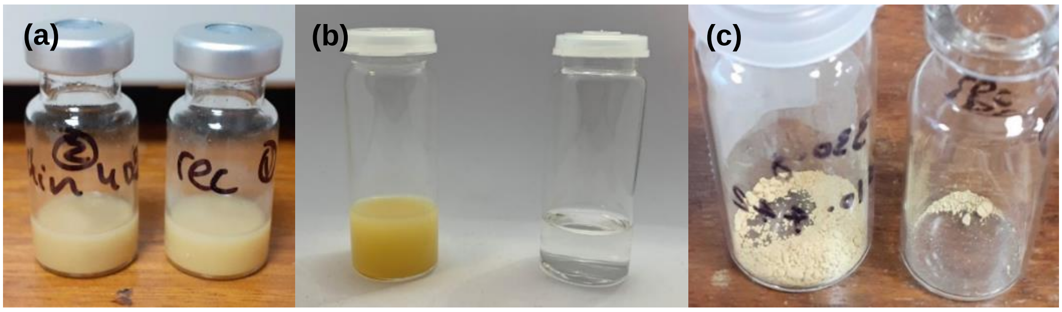

Figure S17: (a) Reaction mixture with new Ni-mpg- CN_x (left) and recovered Ni-mpg- CN_x (right); (b) Reaction mixture before centrifugation (left) and after (right); (c) color of new Ni-mpg- CN_x (left) and recovered Ni-mpg- CN_x

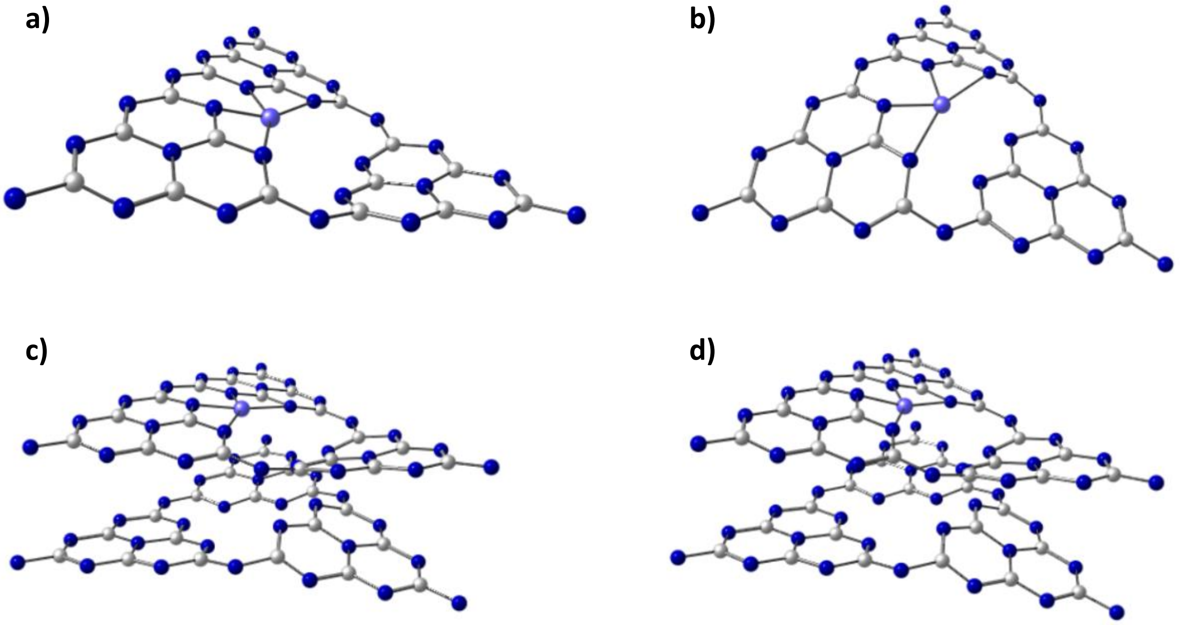

Figure S18: Comparison of the DFT optmised structures (B3LYP/6-31G*) for NiII-mpg-CNx (a) and NiI-mpg-CNx (b) with the ONIOM (B3LYP/6-31G*/UFF) opitmised structures (c and d).

7. NMR spectra of purified products

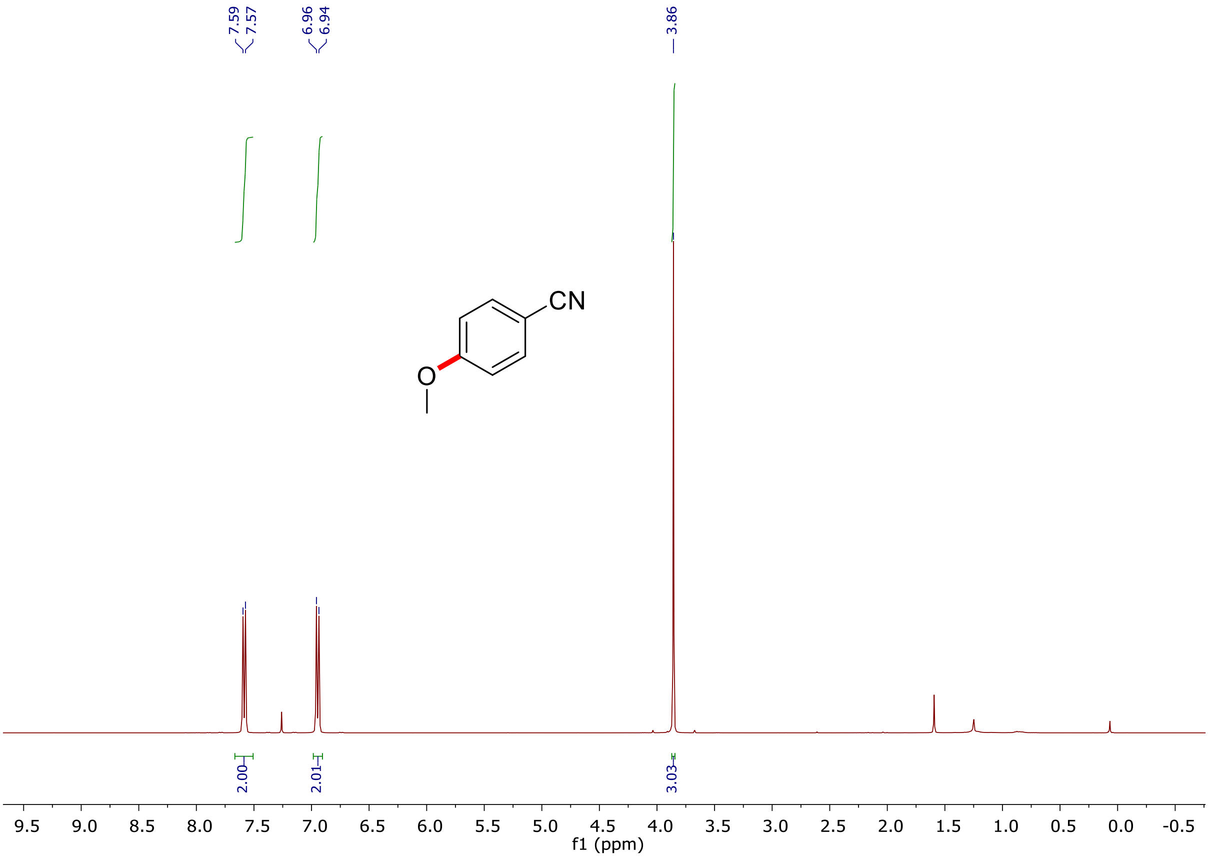

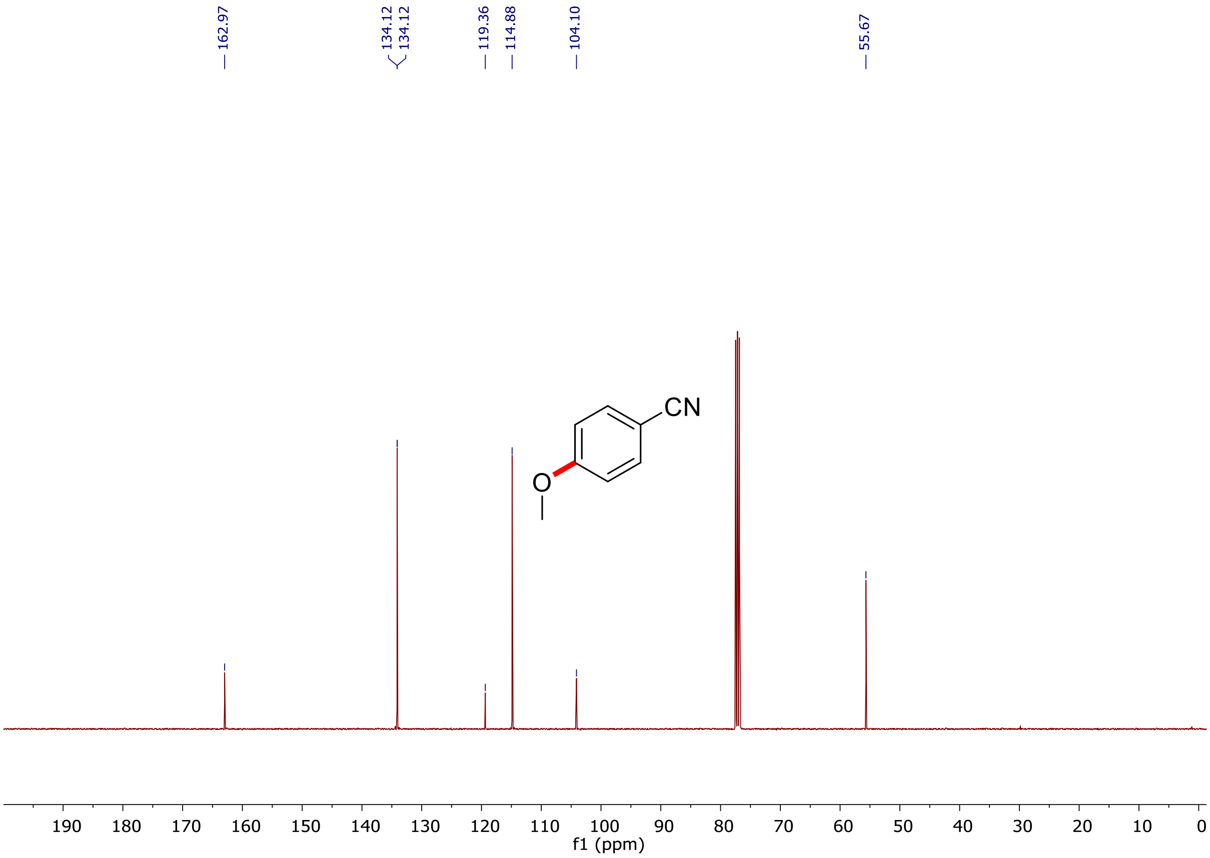

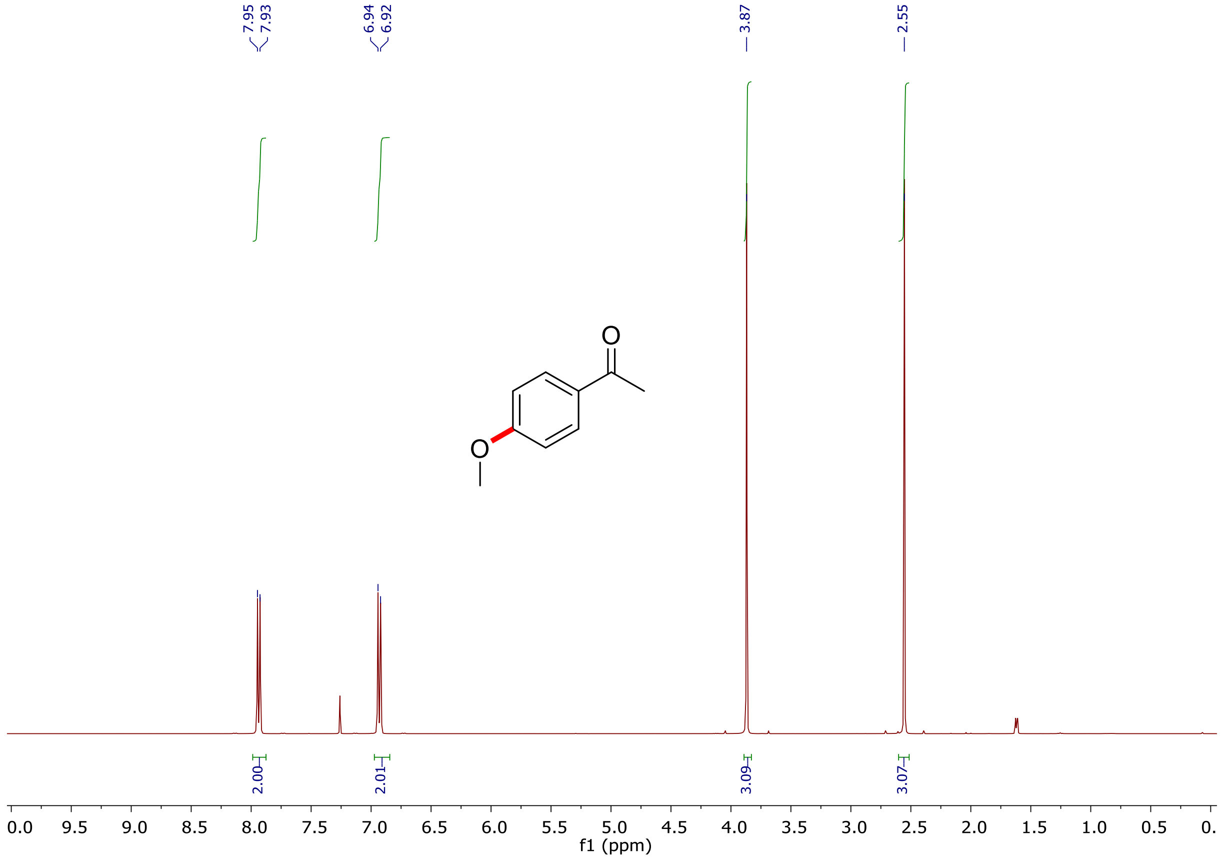
7.95
7.93
6.94
6.92
3.87
2.55
2.00
2.01
3.09
3.07
f1 (ppm)

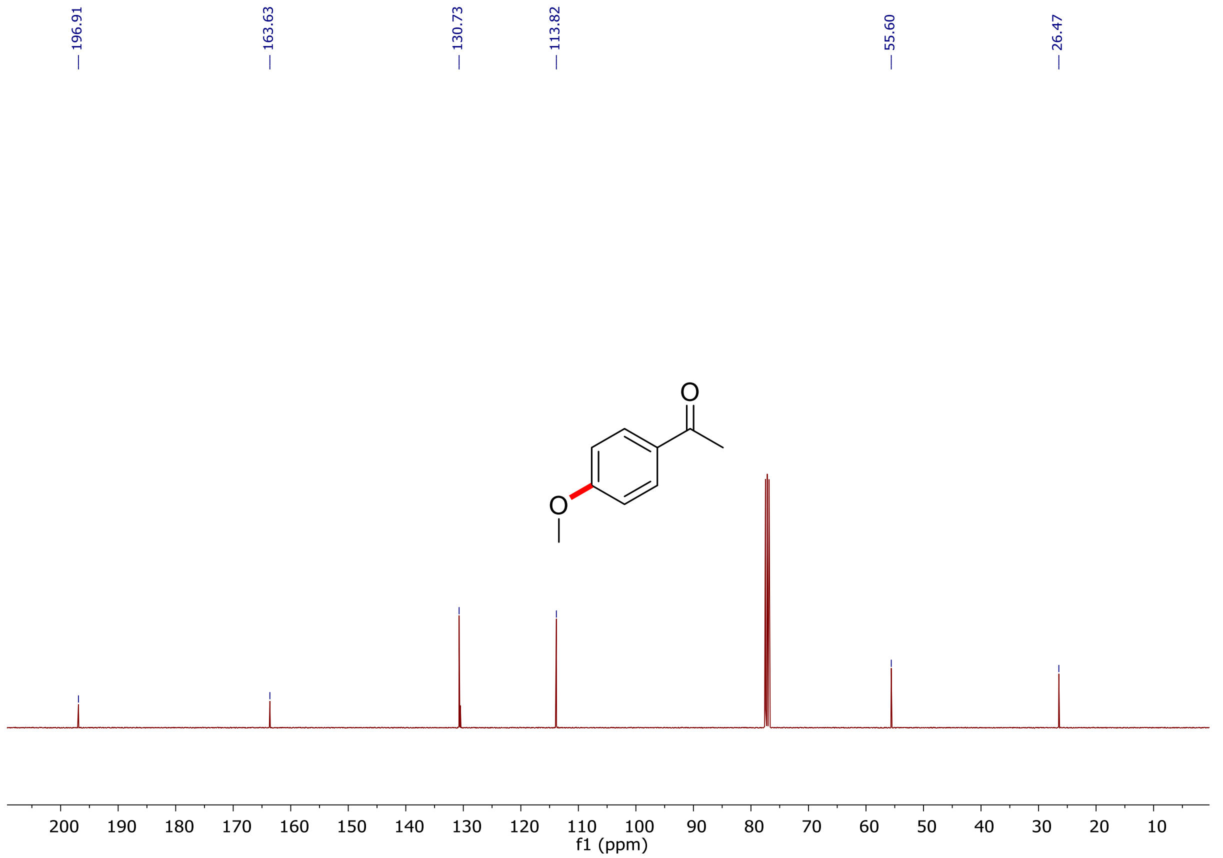
196.91
163.63
130.73
113.82
55.60
26.47
f1 (ppm)

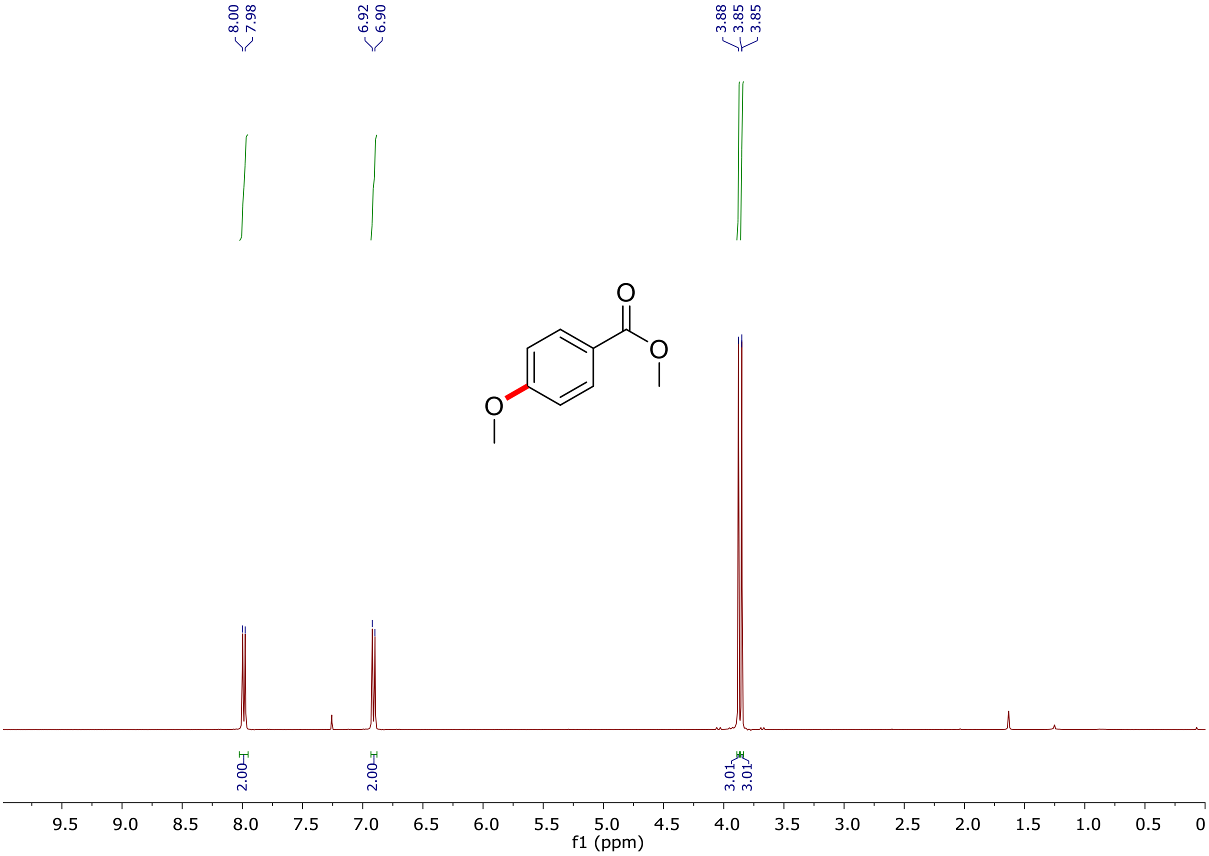
8.00
7.98
6.92
6.90
3.88
3.85
3.85
O
O
O
2.00
2.00
3.01
3.01
9.5 9.0 8.5 8.0 7.5 7.0 6.5 6.0 5.5 5.0 4.5 4.0 3.5 3.0 2.5 2.0 1.5 1.0 0.5 0
f1 (ppm)

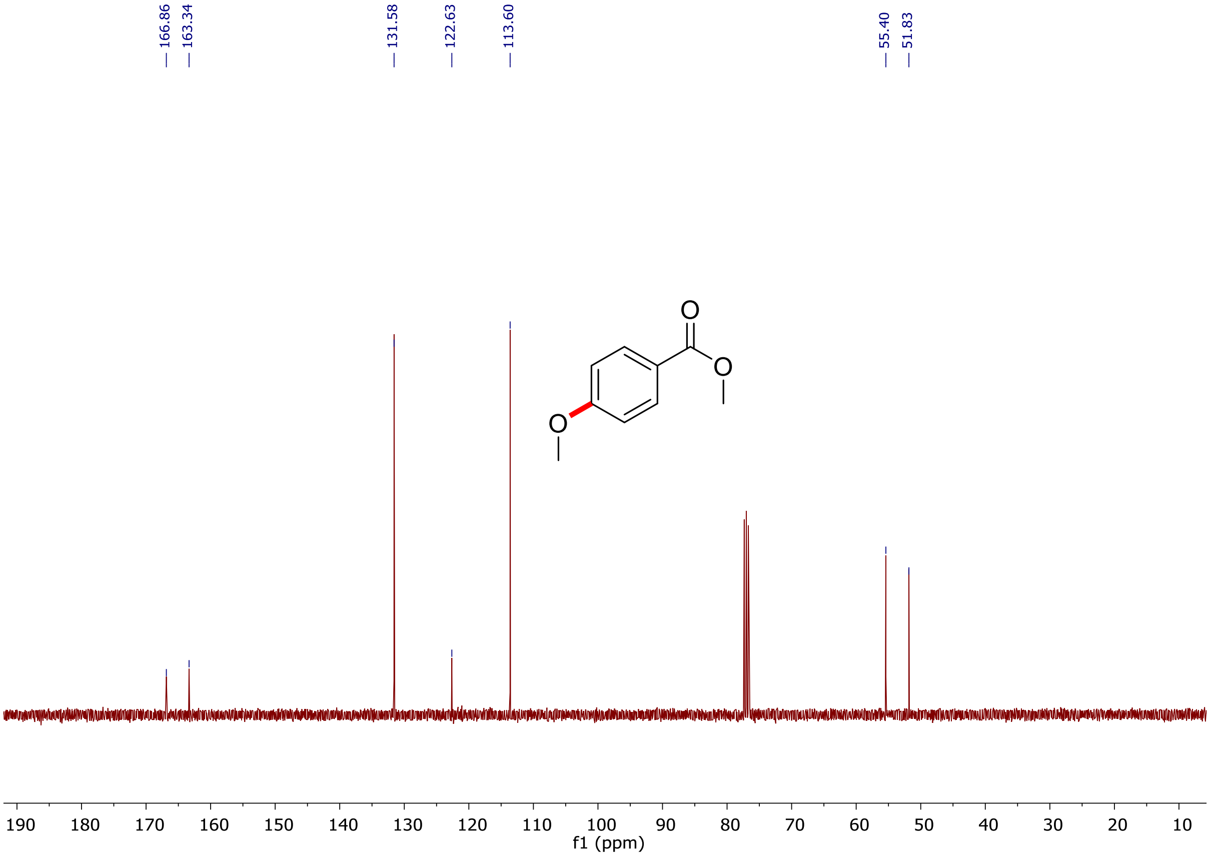
166.86
163.34
131.58
122.63
113.60
55.40
51.83
O
O
O
190 180 170 160 150 140 130 120 110 100 90 80 70 60 50 40 30 20 10
f1 (ppm)

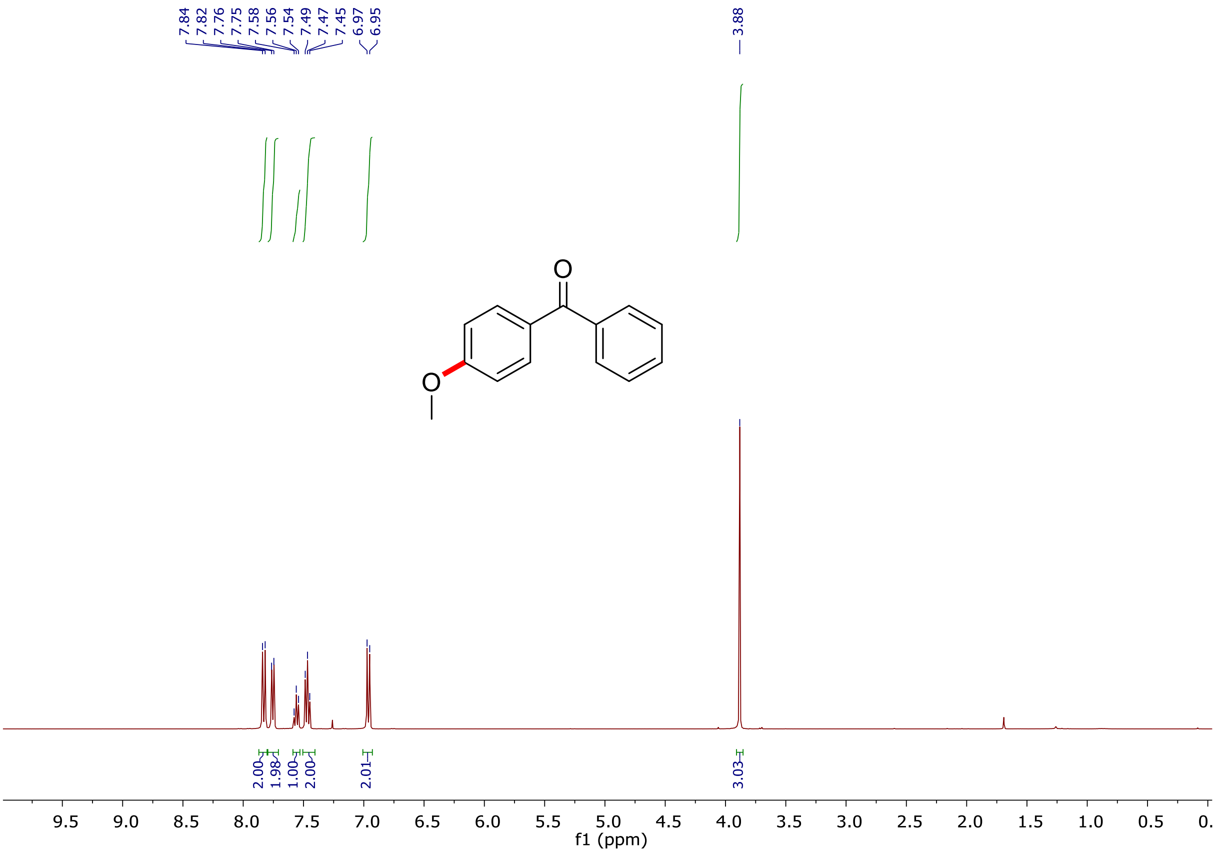
7.84
7.82
7.76
7.75
7.58
7.56
7.54
7.49
7.47
7.45
6.97
6.95
3.88
O
2.00
1.98
1.00
2.00
2.01
3.03
9.5 9.0 8.5 8.0 7.5 7.0 6.5 6.0 5.5 5.0 4.5 4.0 3.5 3.0 2.5 2.0 1.5 1.0 0.5 0.
f1 (ppm)

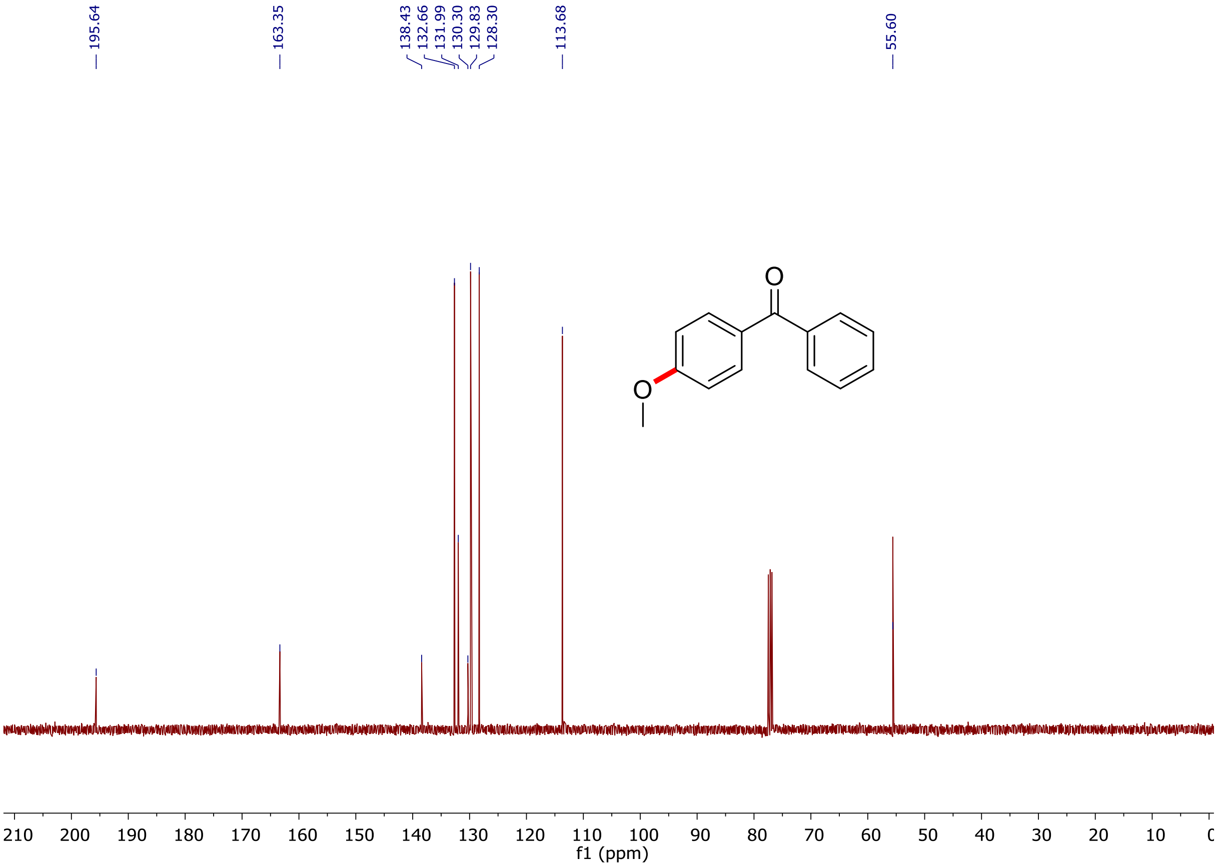
195.64
163.35
138.43
132.66
131.99
130.30
129.83
128.30
113.68
55.60
O
210 200 190 180 170 160 150 140 130 120 110 100 90 80 70 60 50 40 30 20 10 0
f1 (ppm)

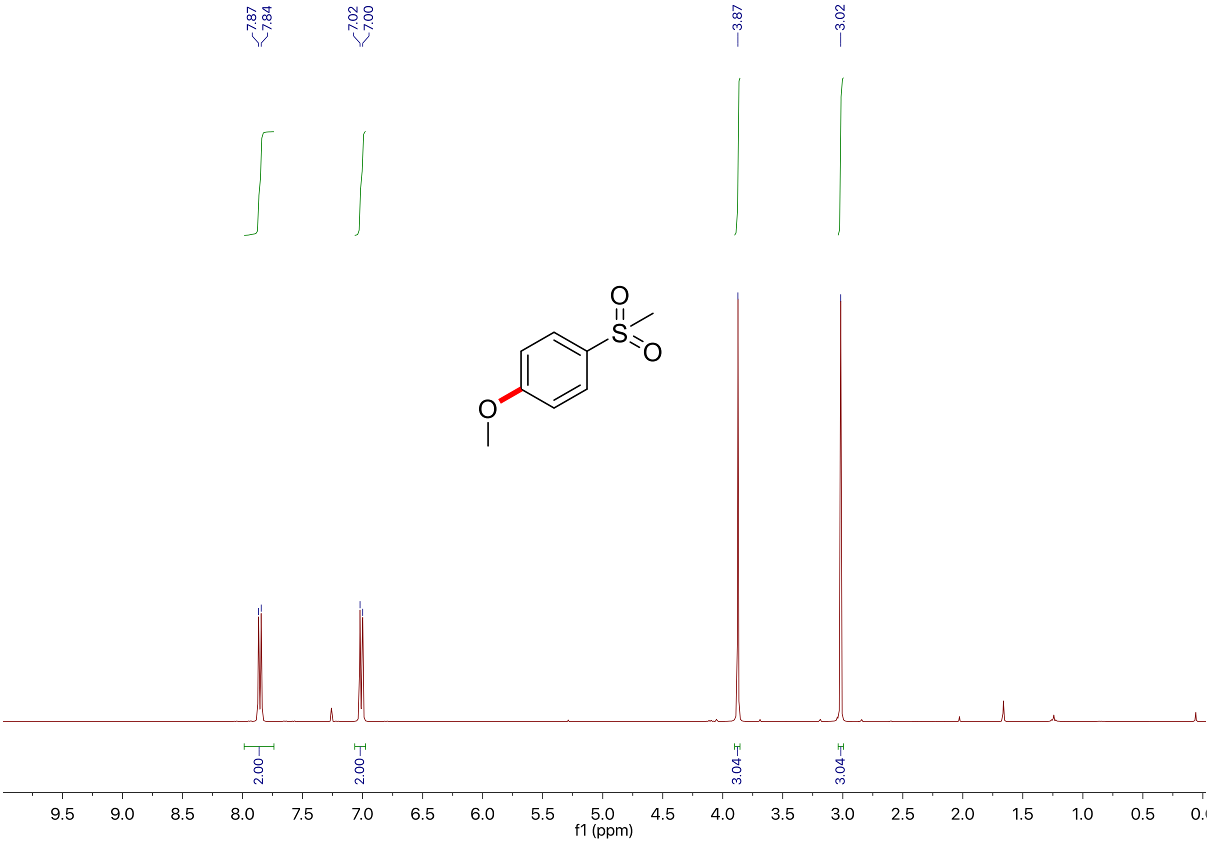
7.87
7.84
7.02
7.00
3.87
3.02
O
S
O
O
2.00
2.00
3.04
3.04
9.5
9.0
8.5
8.0
7.5
7.0
6.5
6.0
5.5
5.0
4.5
4.0
3.5
3.0
2.5
2.0
1.5
1.0
0.5
f1 (ppm)

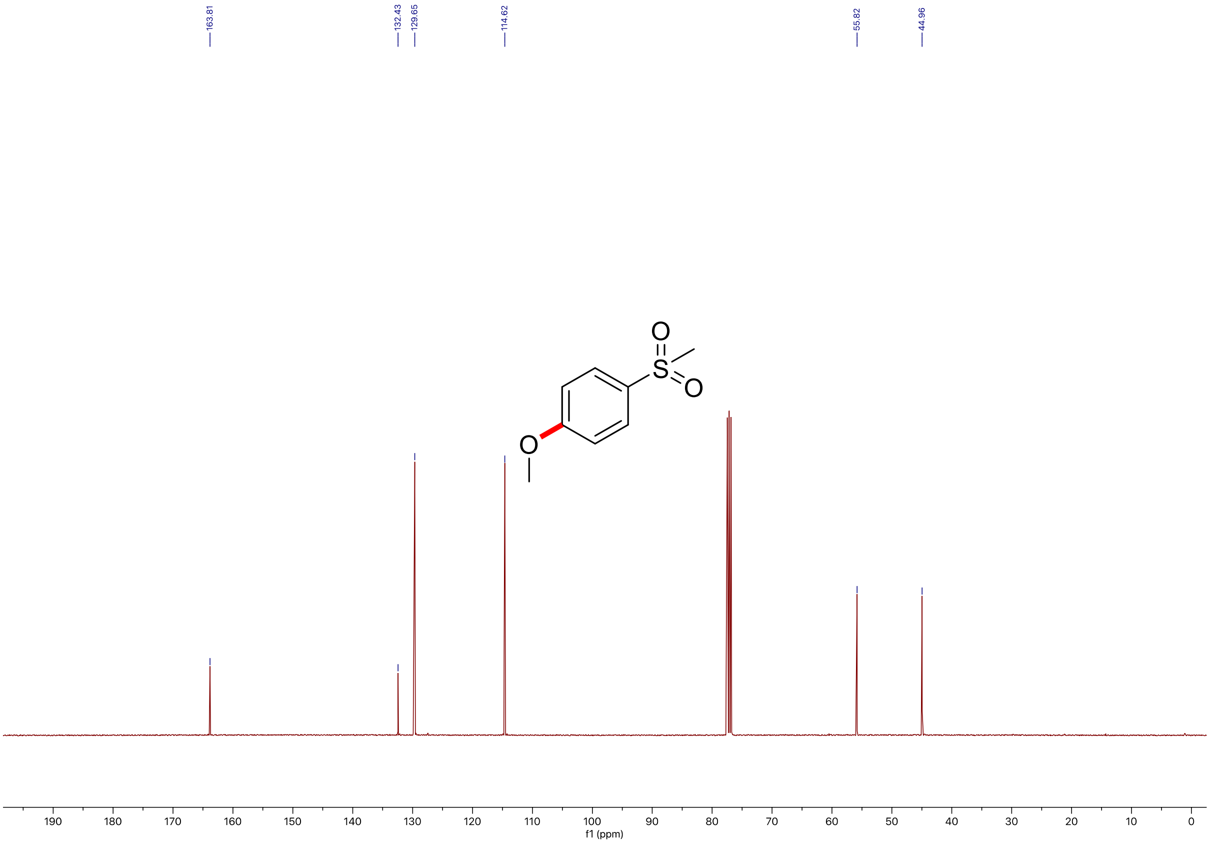
163.81
132.43
129.65
114.62
55.82
44.96
O
S
O
O
190
180
170
160
150
140
130
120
110
100
90
80
70
60
50
40
30
20
10
0
f1 (ppm)

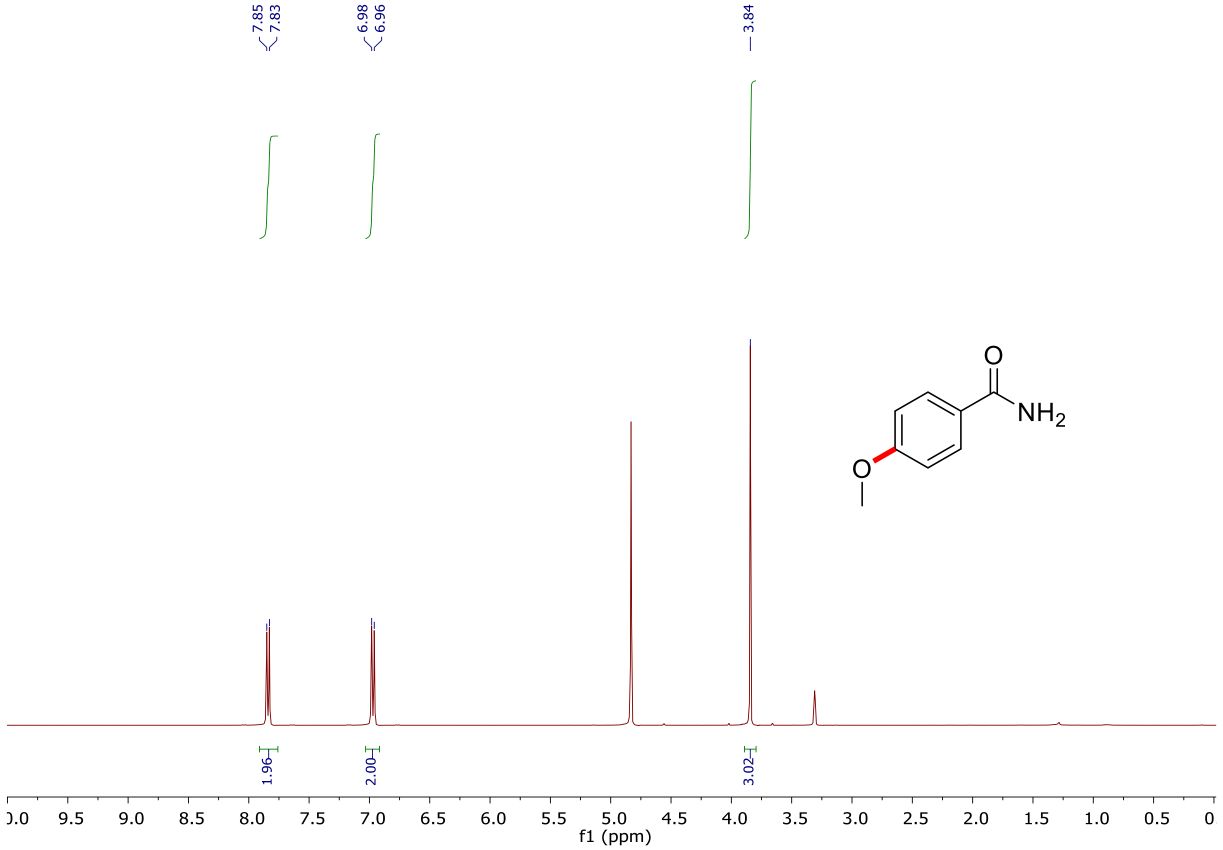
7.85
7.83
6.98
6.96
3.84
1.96
2.00
3.02
O
NH2
O
f1 (ppm)

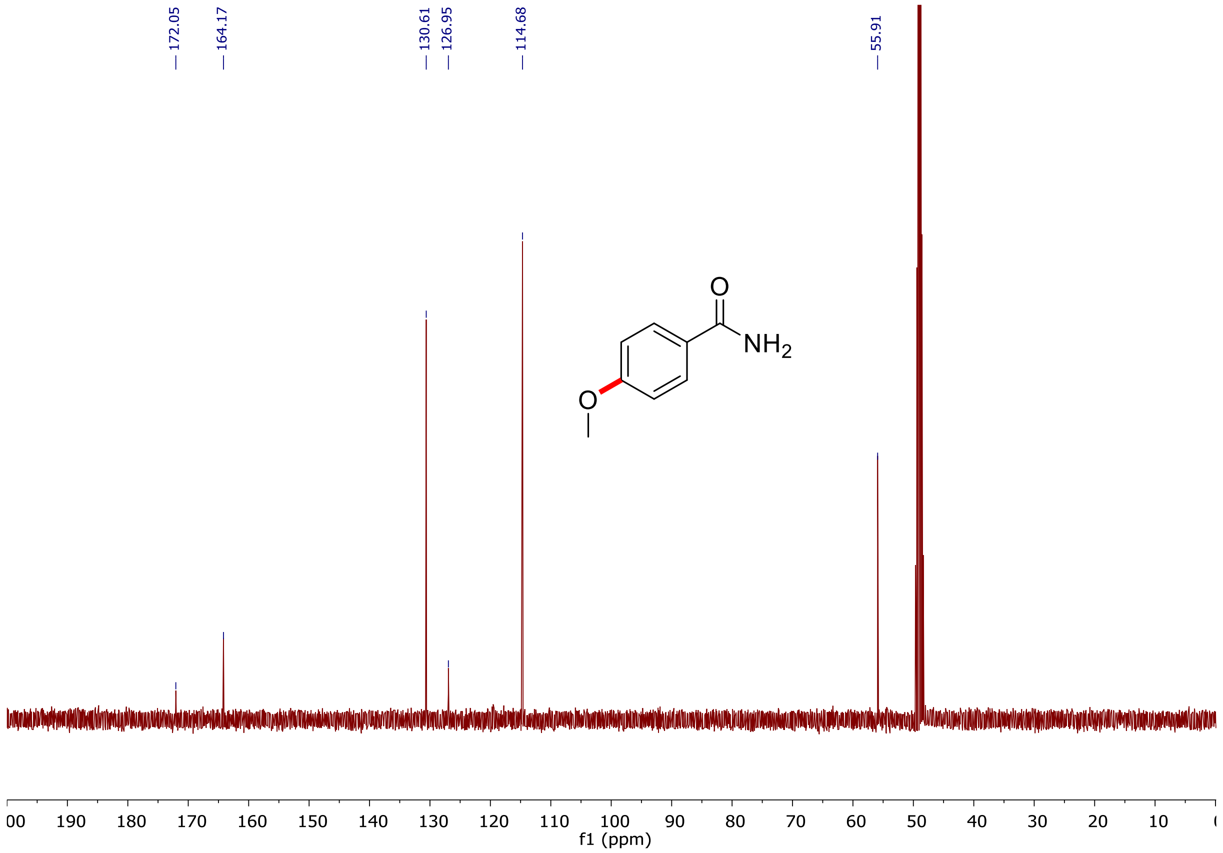
172.05
164.17
130.61
126.95
114.68
55.91
O
NH2
O
f1 (ppm)

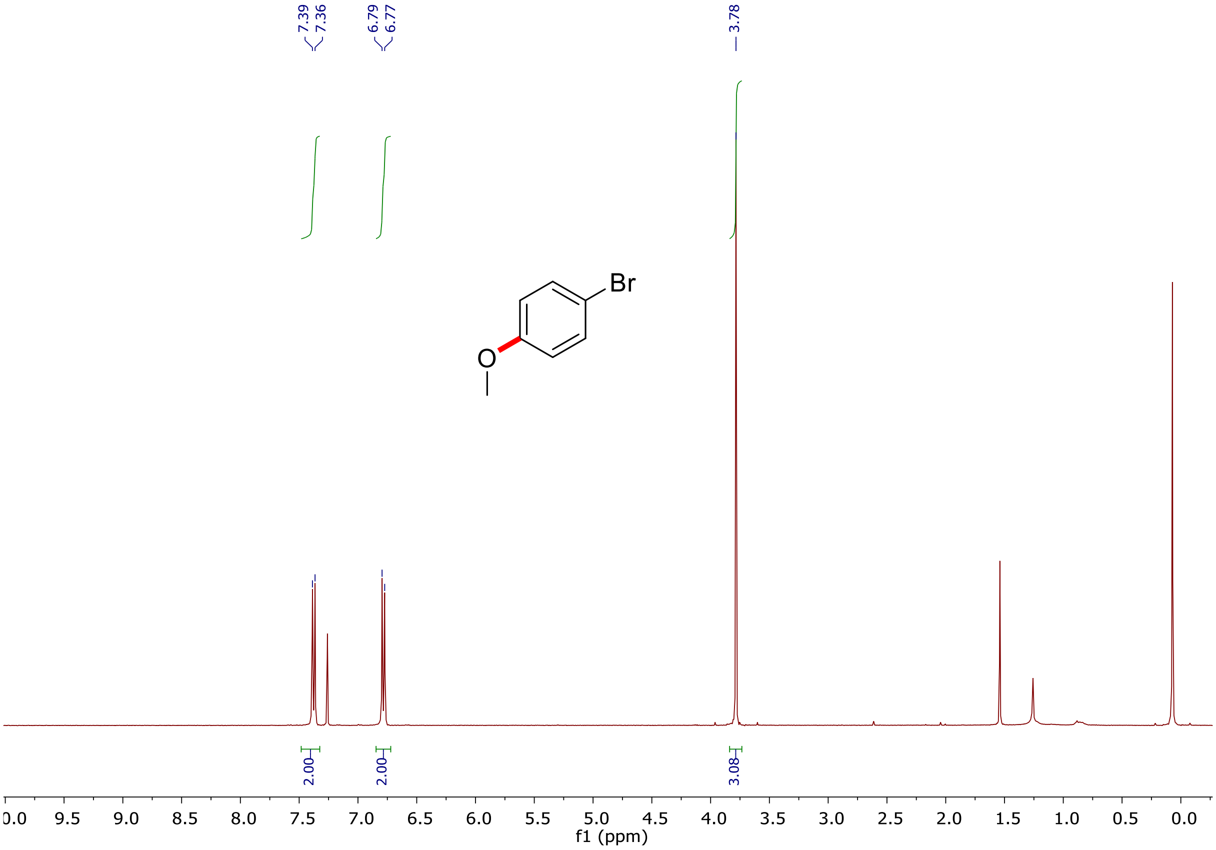
7.39
7.36
6.79
6.77
3.78
Br
O
2.00
2.00
3.08
f1 (ppm)

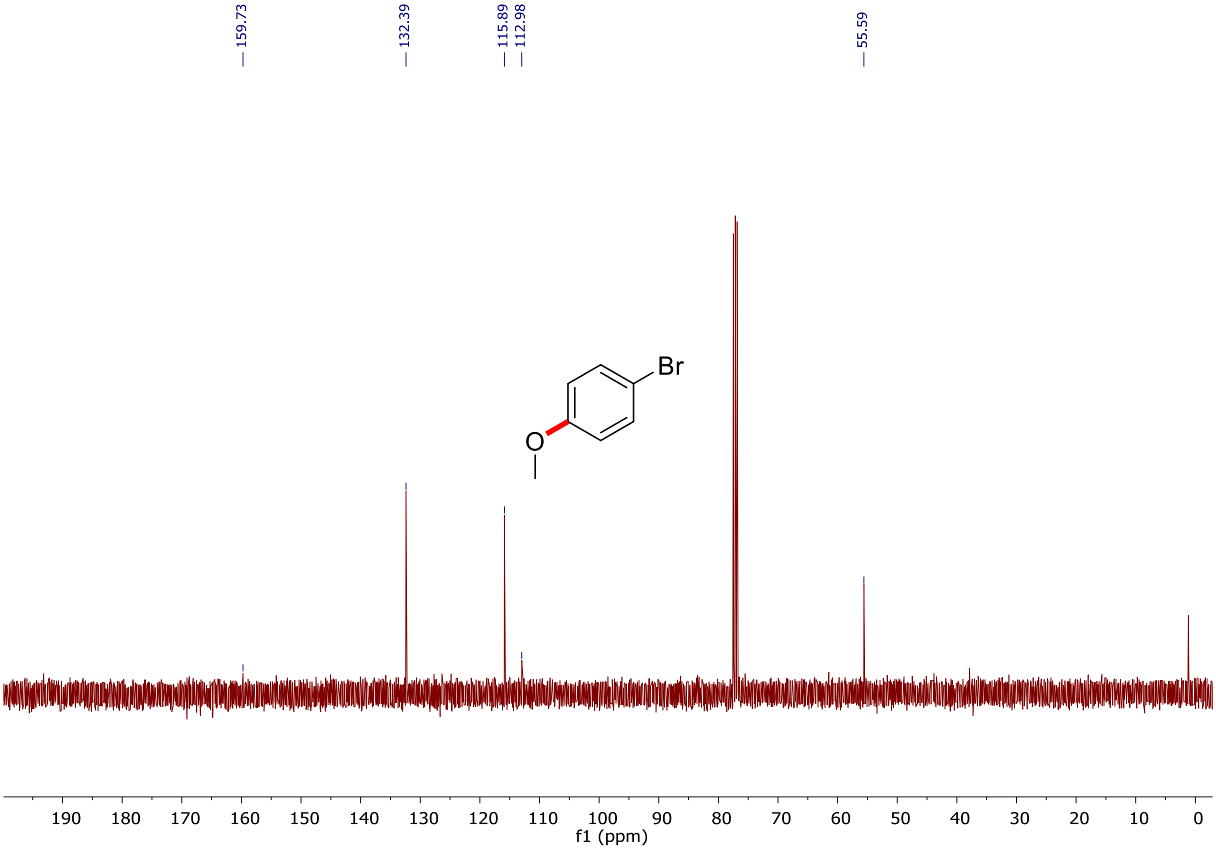
159.73
132.39
115.89
112.98
55.59
Br
O
f1 (ppm)

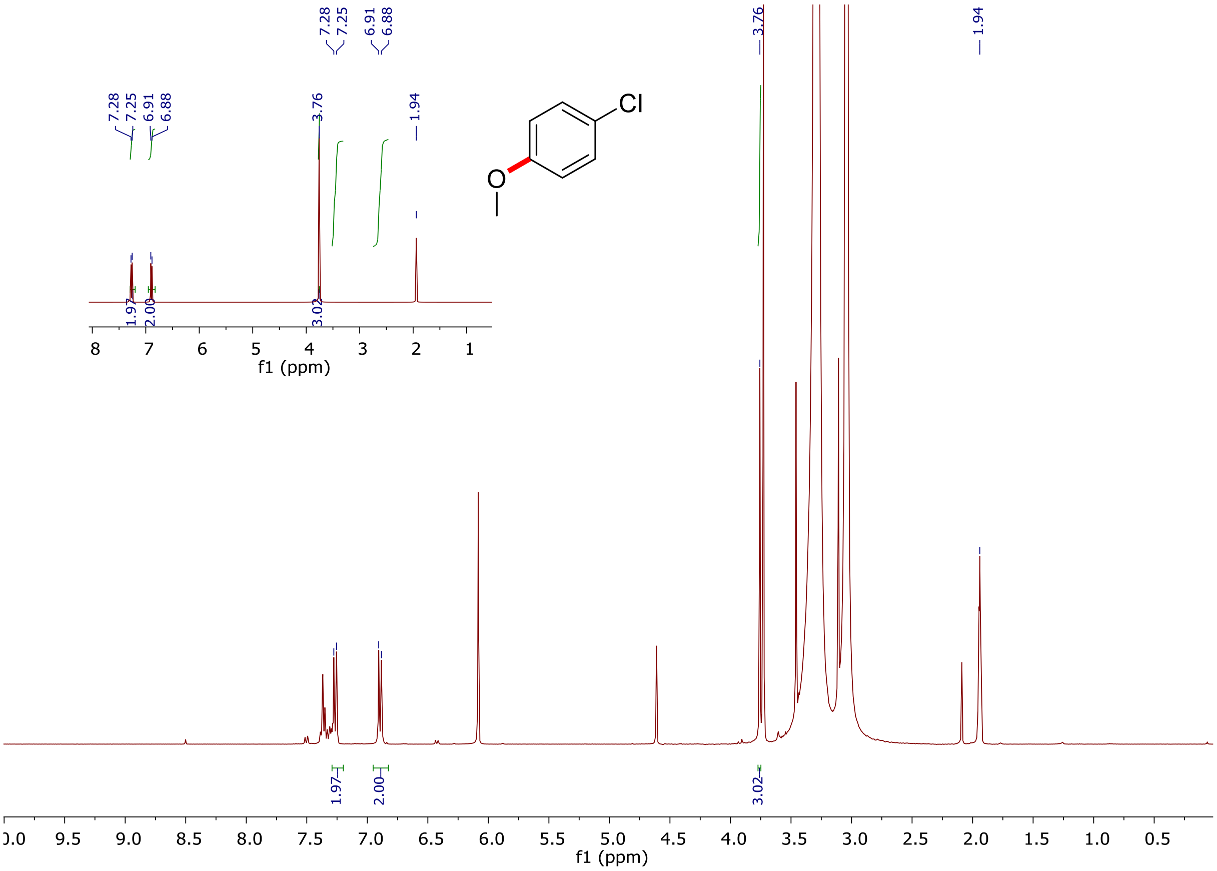
7.28
7.25
6.91
6.88
3.76
1.94
Cl
O
1.97
2.00
3.02
f1 (ppm)

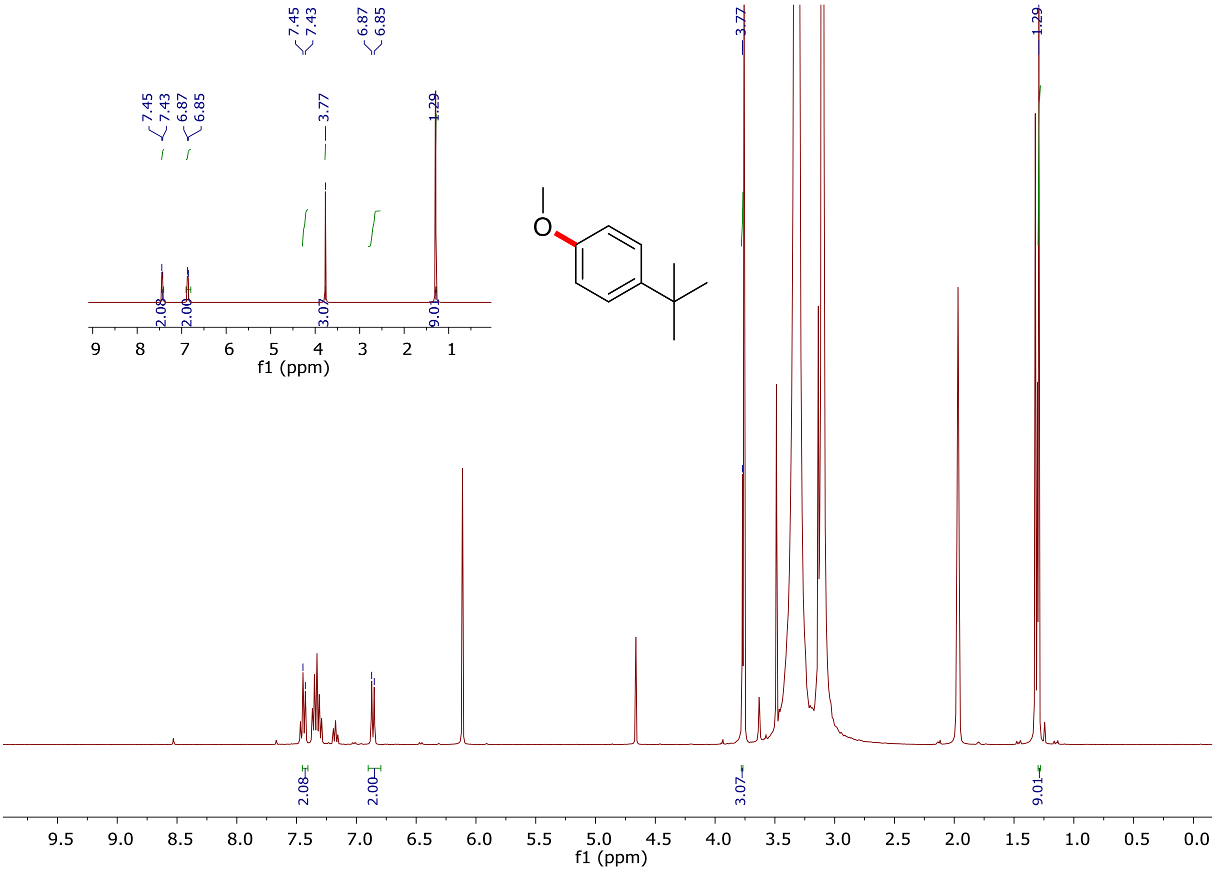
7.45
7.43
6.87
6.85
3.77
1.29
O
2.08
2.00
3.07
9.01
f1 (ppm)

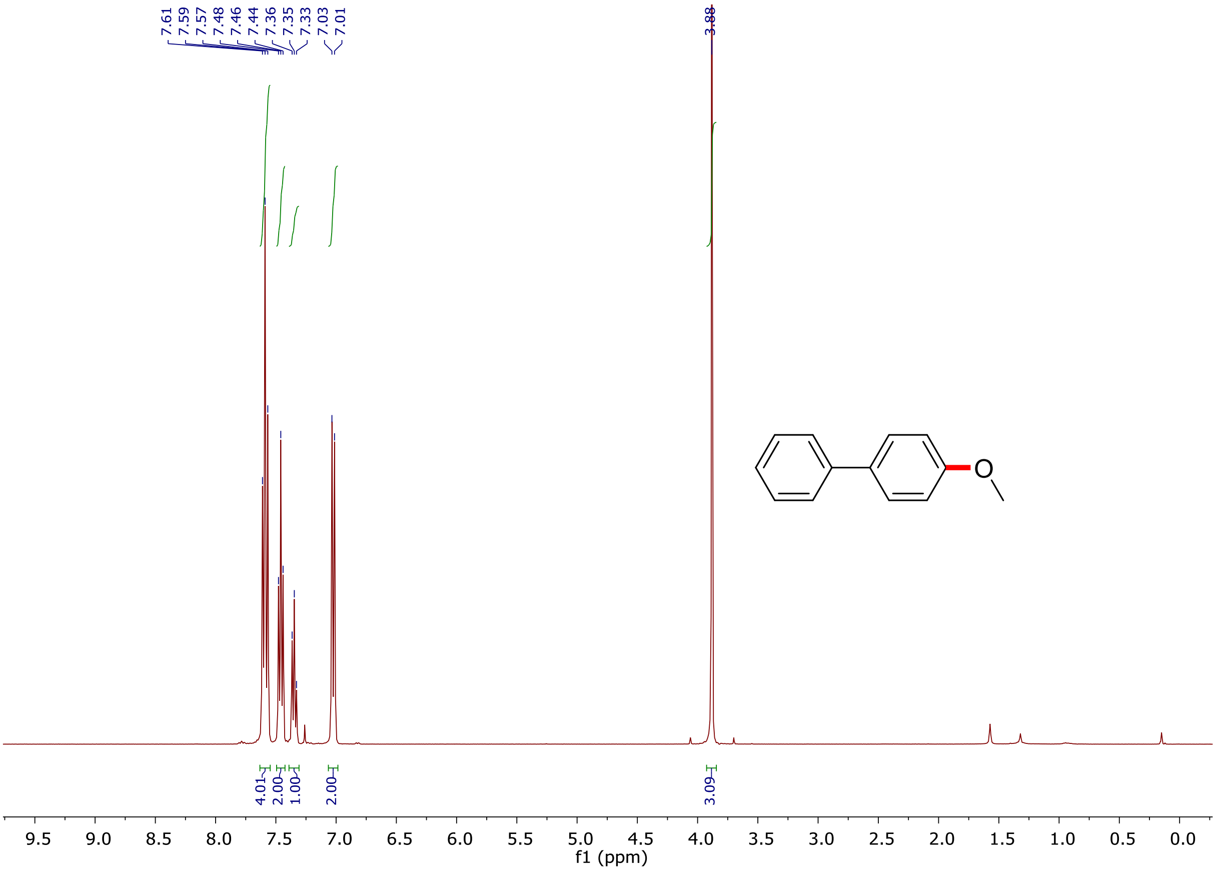
7.61
7.59
7.57
7.48
7.46
7.44
7.36
7.35
7.33
7.03
7.01
3.88
4.01
2.00
1.00
2.00
3.09
9.5 9.0 8.5 8.0 7.5 7.0 6.5 6.0 5.5 5.0 4.5 4.0 3.5 3.0 2.5 2.0 1.5 1.0 0.5 0.0
f1 (ppm)
O

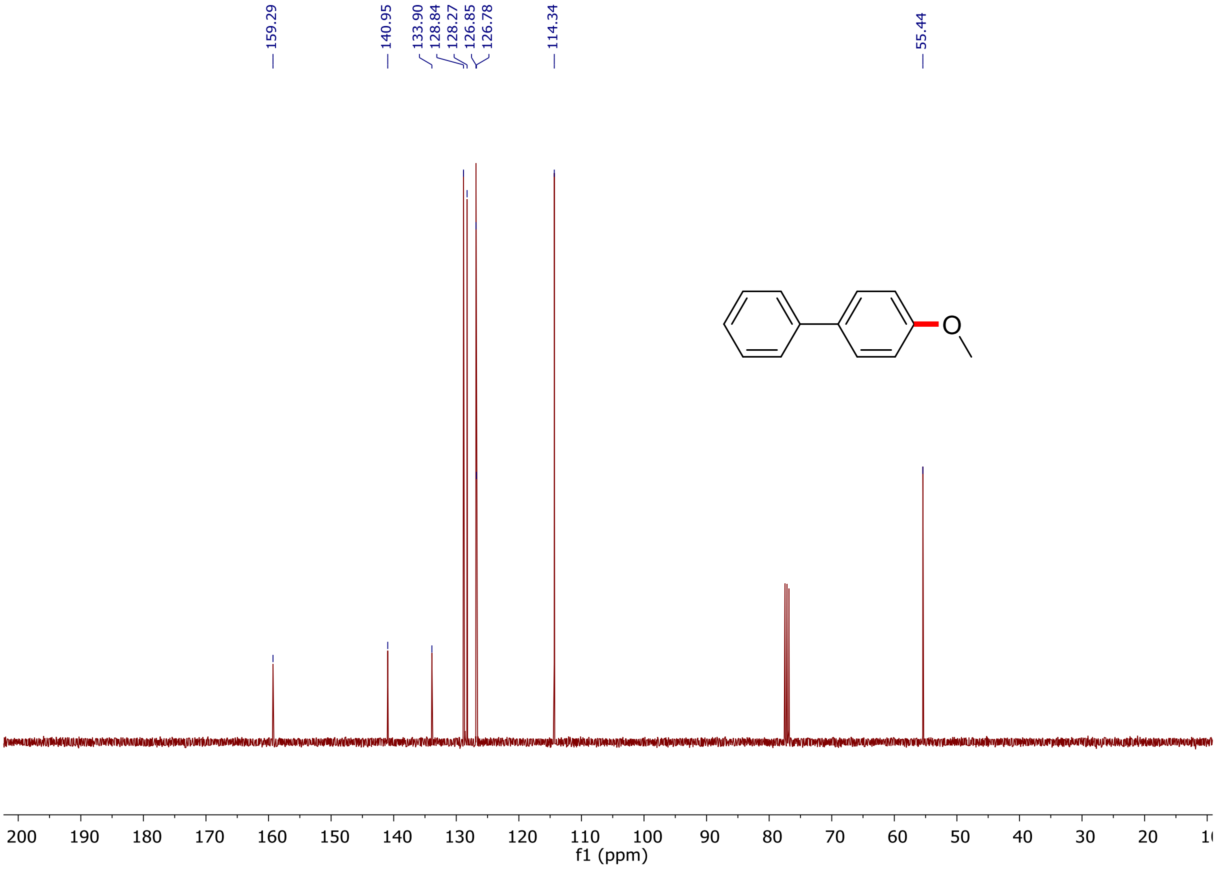
159.29
140.95
133.90
128.84
128.27
126.85
126.78
114.34
55.44
200 190 180 170 160 150 140 130 120 110 100 90 80 70 60 50 40 30 20 10
f1 (ppm)
O

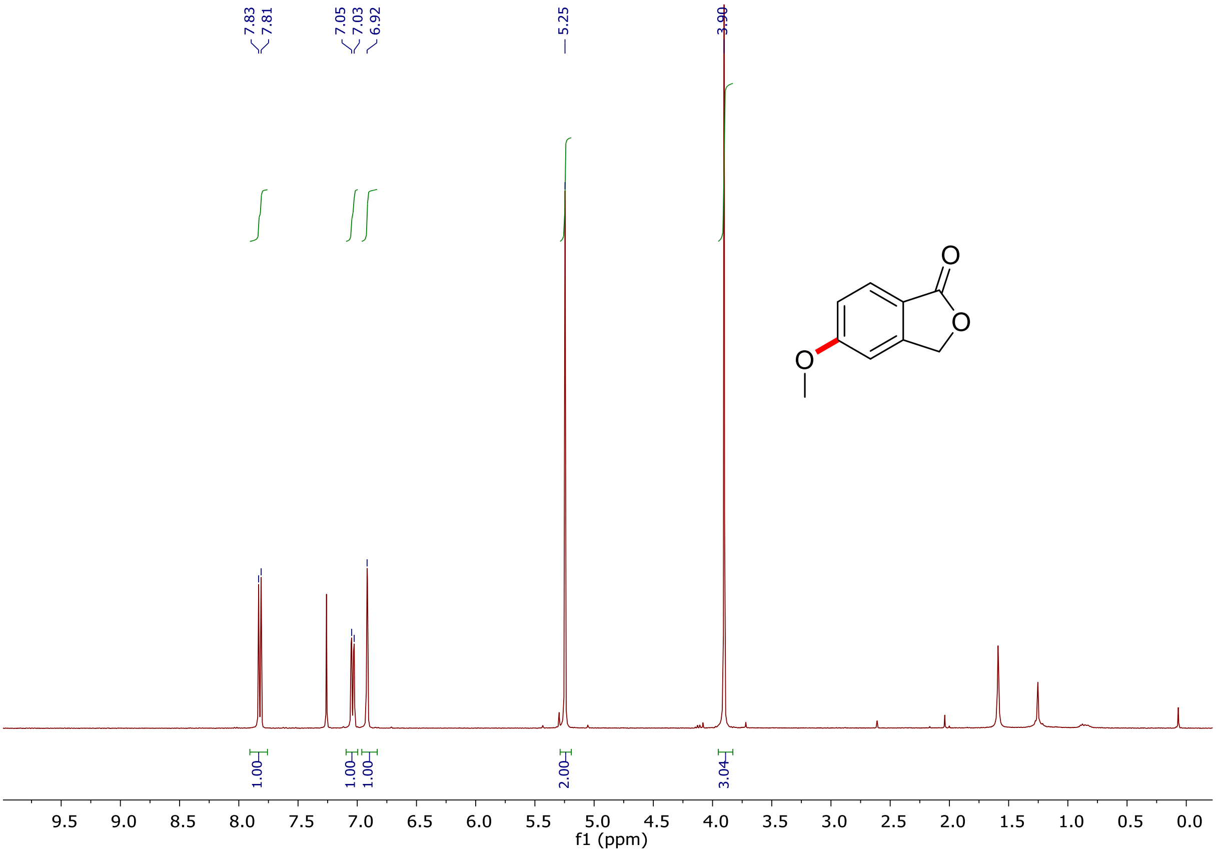
7.83
7.81
7.05
7.03
6.92
5.25
3.90
O
O
O
1.00
1.00
1.00
2.00
3.04
9.5
9.0
8.5
8.0
7.5
7.0
6.5
6.0
5.5
5.0
4.5
4.0
3.5
3.0
2.5
2.0
1.5
1.0
0.5
0.0
f1 (ppm)

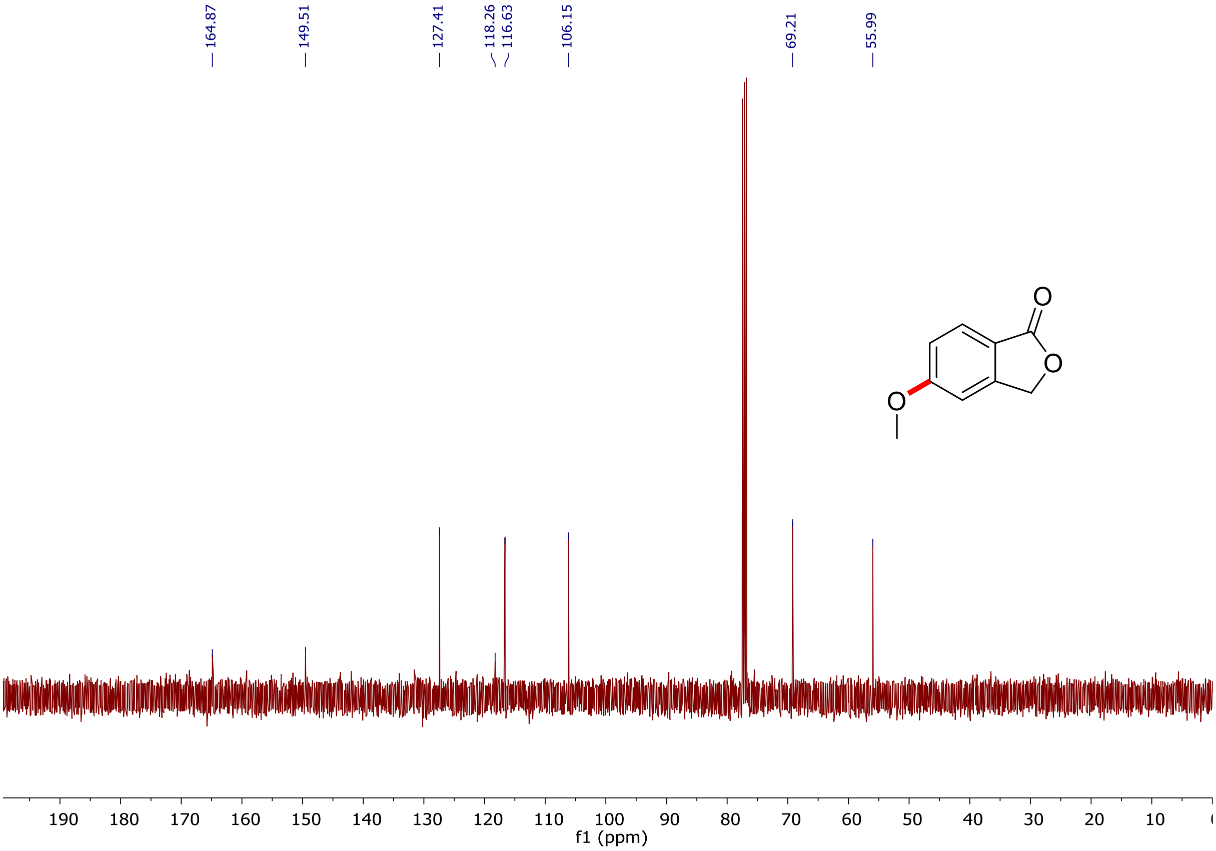
164.87
149.51
127.41
118.26
116.63
106.15
69.21
55.99
O
O
O
190
180
170
160
150
140
130
120
110
100
90
80
70
60
50
40
30
20
10
f1 (ppm)

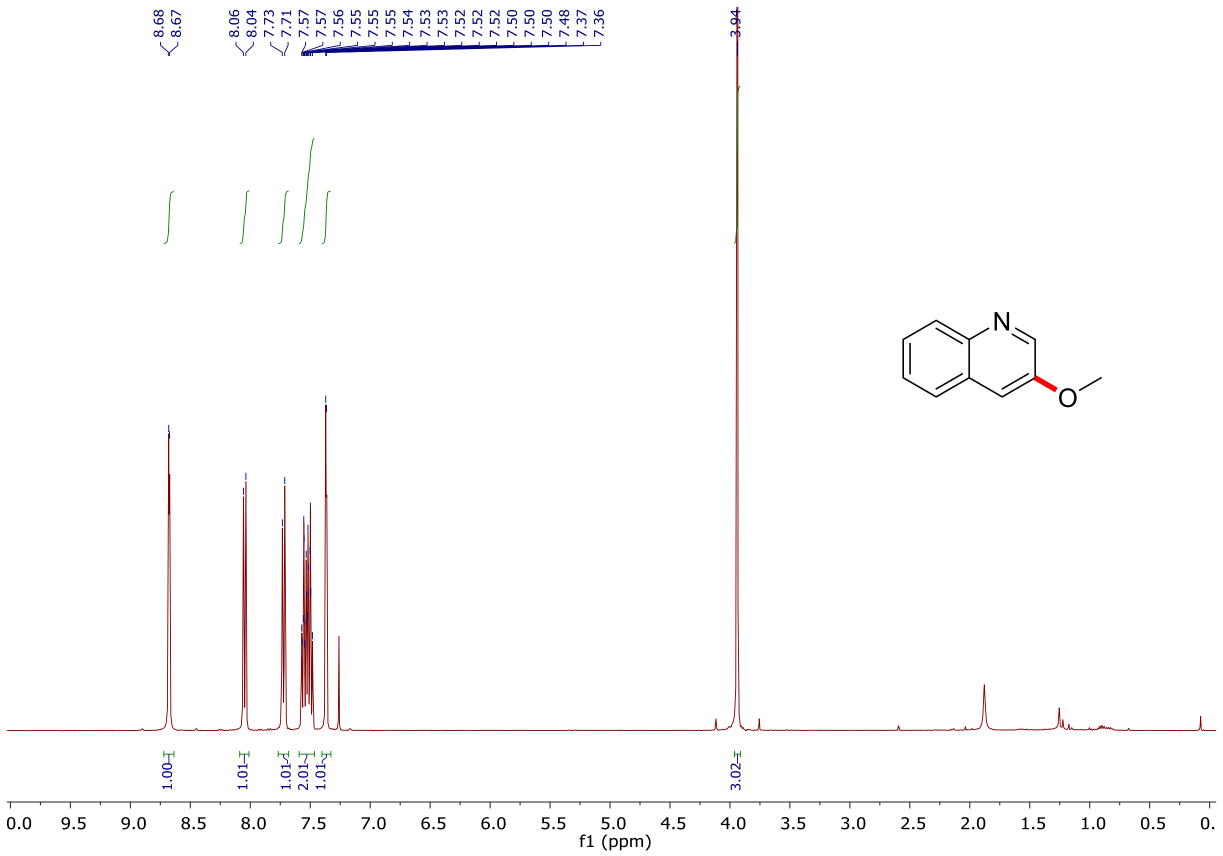

8.68
8.67
8.06
8.04
7.73
7.71
7.57
7.57
7.56
7.55
7.55
7.55
7.54
7.53
7.53
7.52
7.52
7.52
7.50
7.50
7.50
7.48
7.37
7.36
3.94
N
O
1.00
1.01
1.01
2.01
1.01
3.02
f1 (ppm)

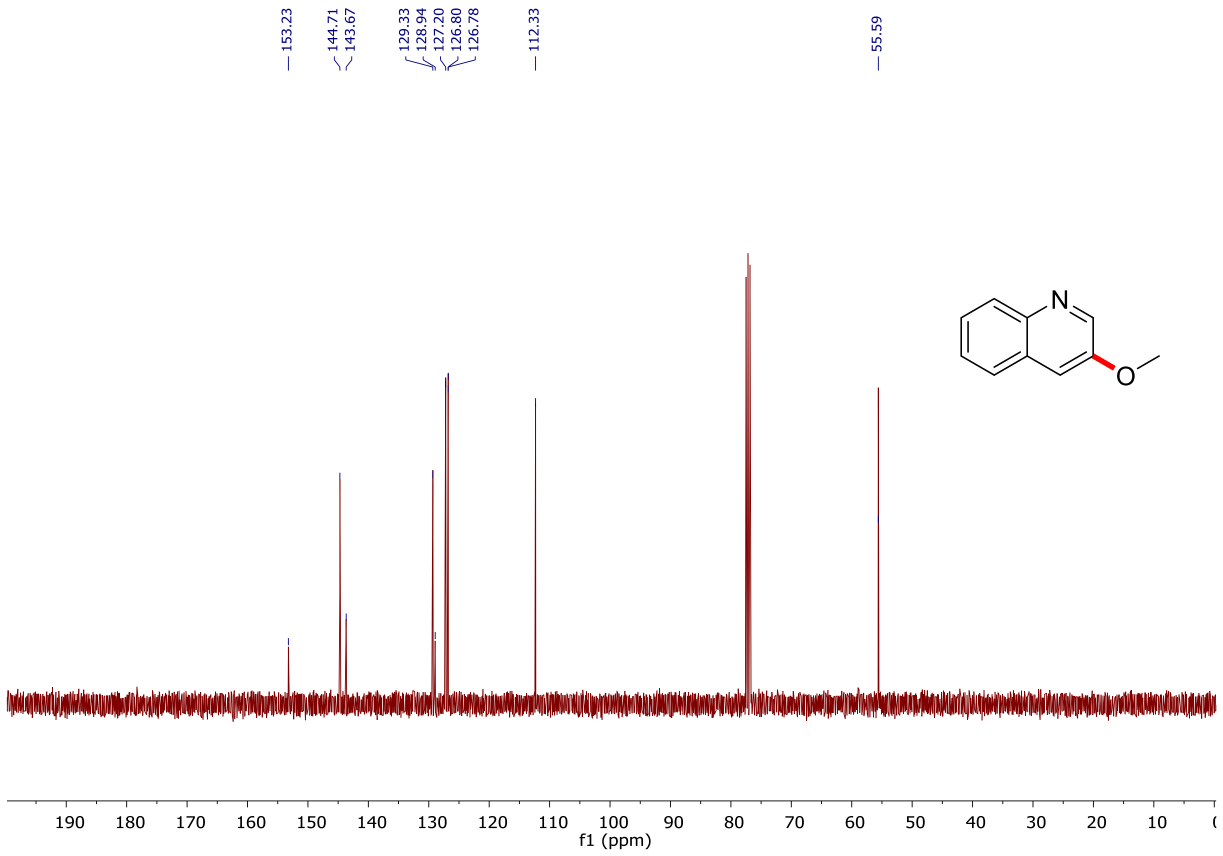

153.23
144.71
143.67
129.33
128.94
127.20
126.80
126.78
112.33
55.59
N
O
f1 (ppm)

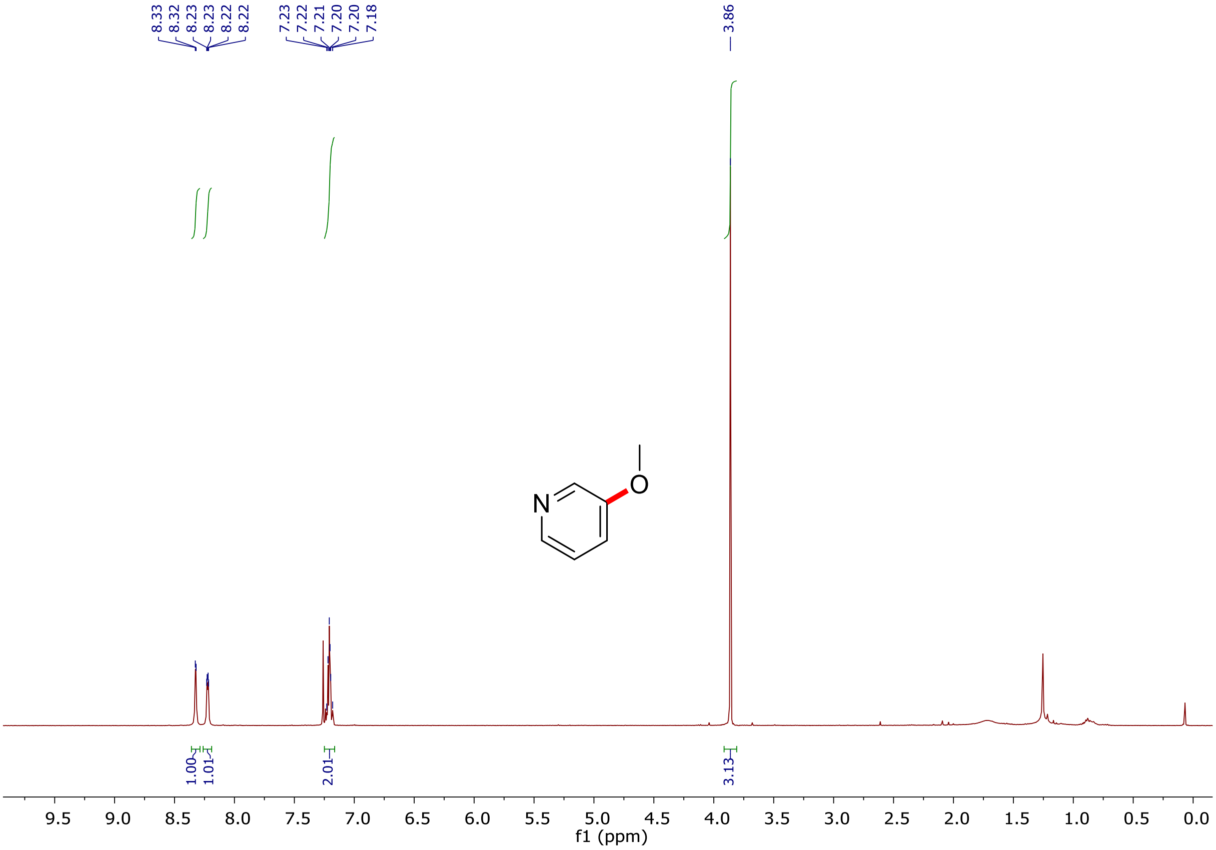

8.33
8.32
8.23
8.23
8.22
8.22
7.23
7.22
7.21
7.20
7.20
7.18
3.86
N
O
1.00
1.01
2.01
3.13
9.5 9.0 8.5 8.0 7.5 7.0 6.5 6.0 5.5 5.0 4.5 4.0 3.5 3.0 2.5 2.0 1.5 1.0 0.5 0.0
f1 (ppm)

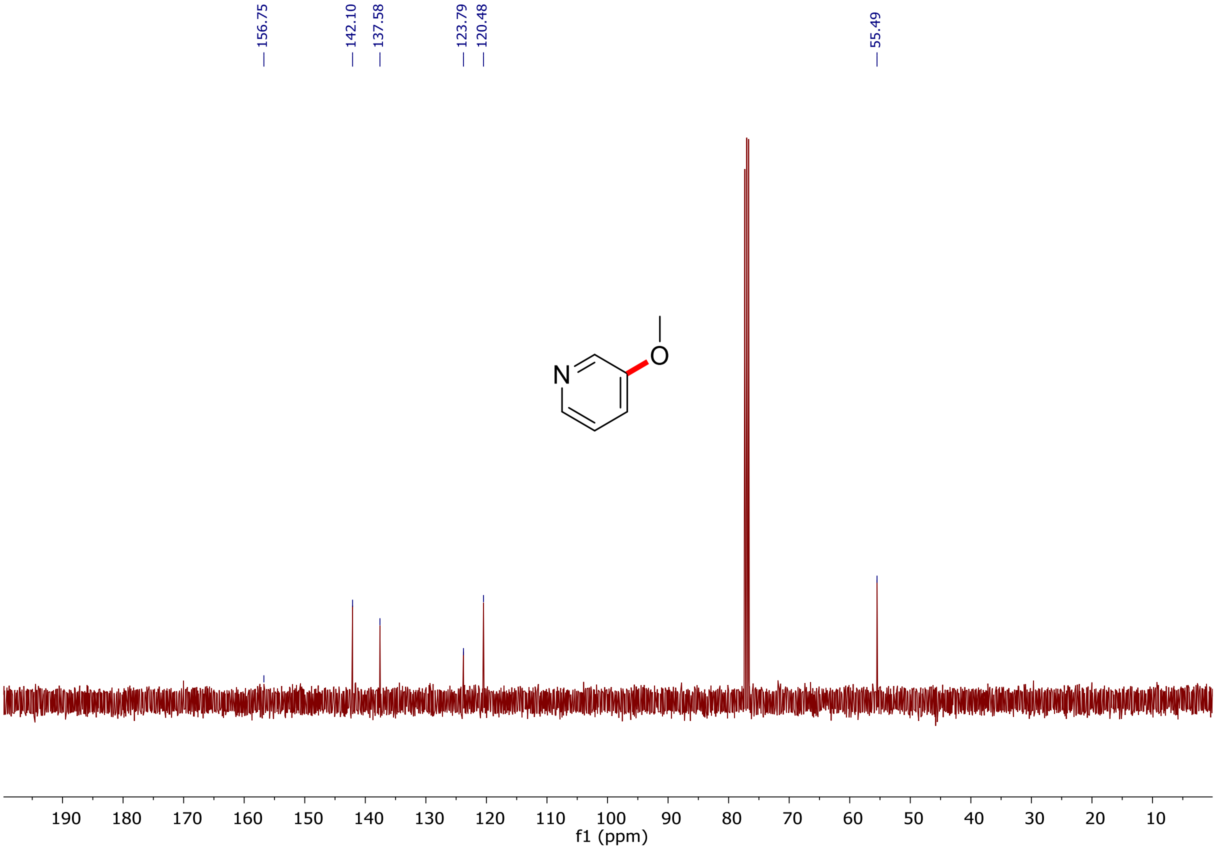

156.75
142.10
137.58
123.79
120.48
55.49
N
O
190 180 170 160 150 140 130 120 110 100 90 80 70 60 50 40 30 20 10
f1 (ppm)

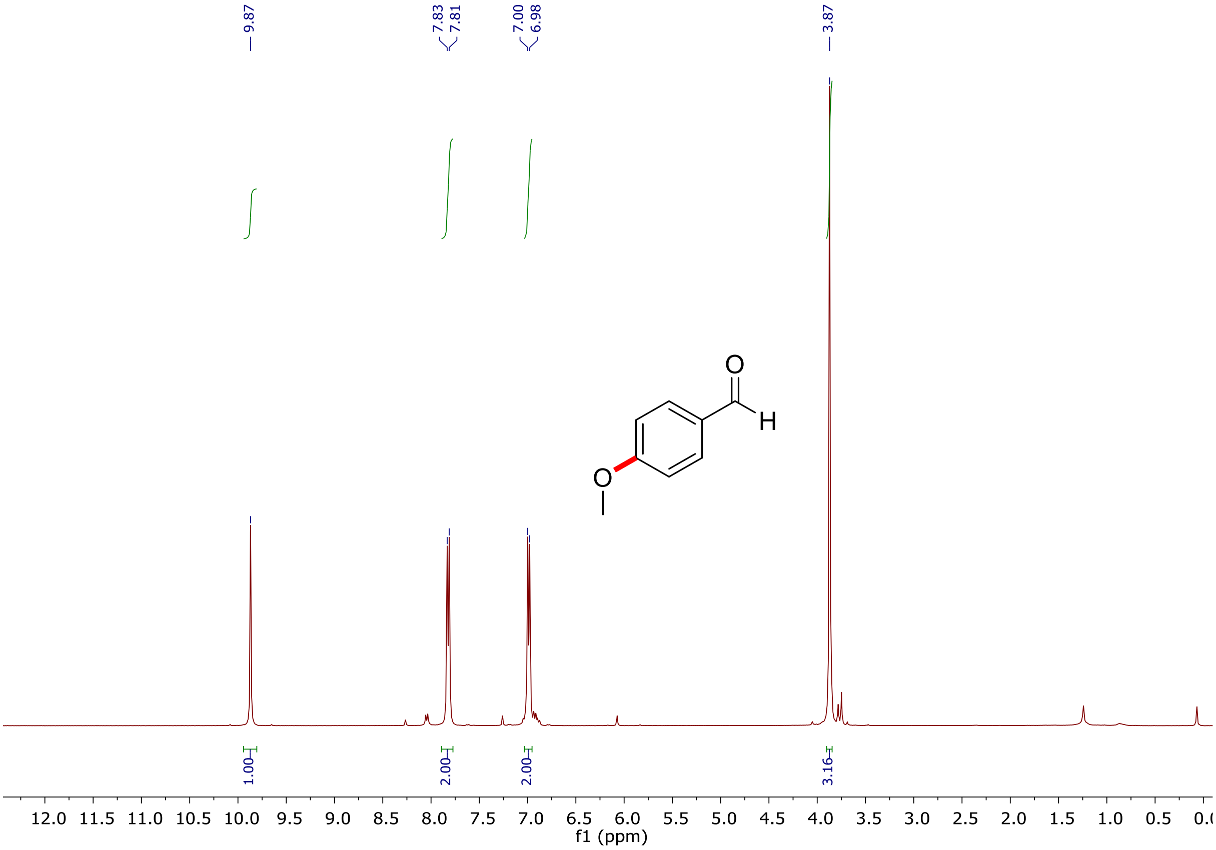
9.87
7.83
7.81
7.00
6.98
3.87
O
H
O
1.00
2.00
2.00
3.16
12.0 11.5 11.0 10.5 10.0 9.5 9.0 8.5 8.0 7.5 7.0 6.5 6.0 5.5 5.0 4.5 4.0 3.5 3.0 2.5 2.0 1.5 1.0 0.5
f1 (ppm)

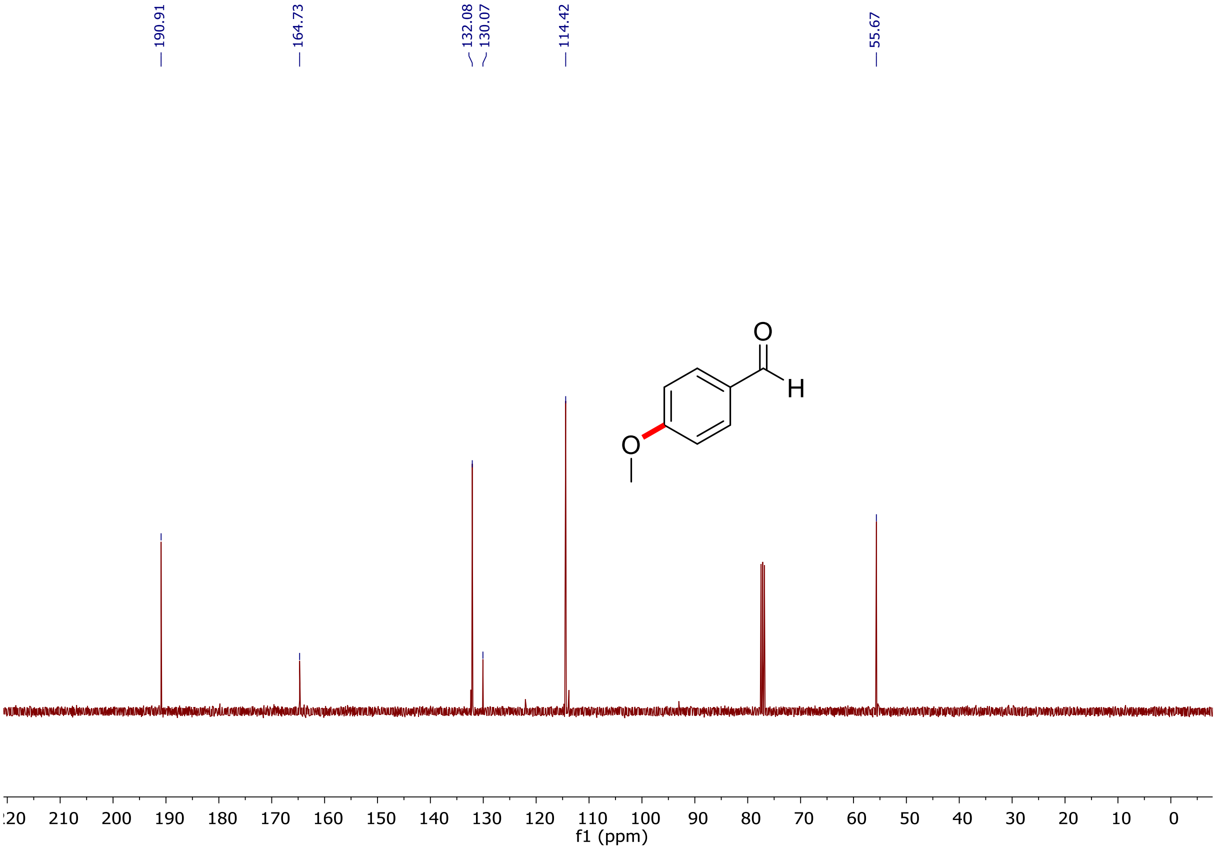
190.91
164.73
132.08
130.07
114.42
55.67
O
H
O
210 200 190 180 170 160 150 140 130 120 110 100 90 80 70 60 50 40 30 20 10 0
f1 (ppm)

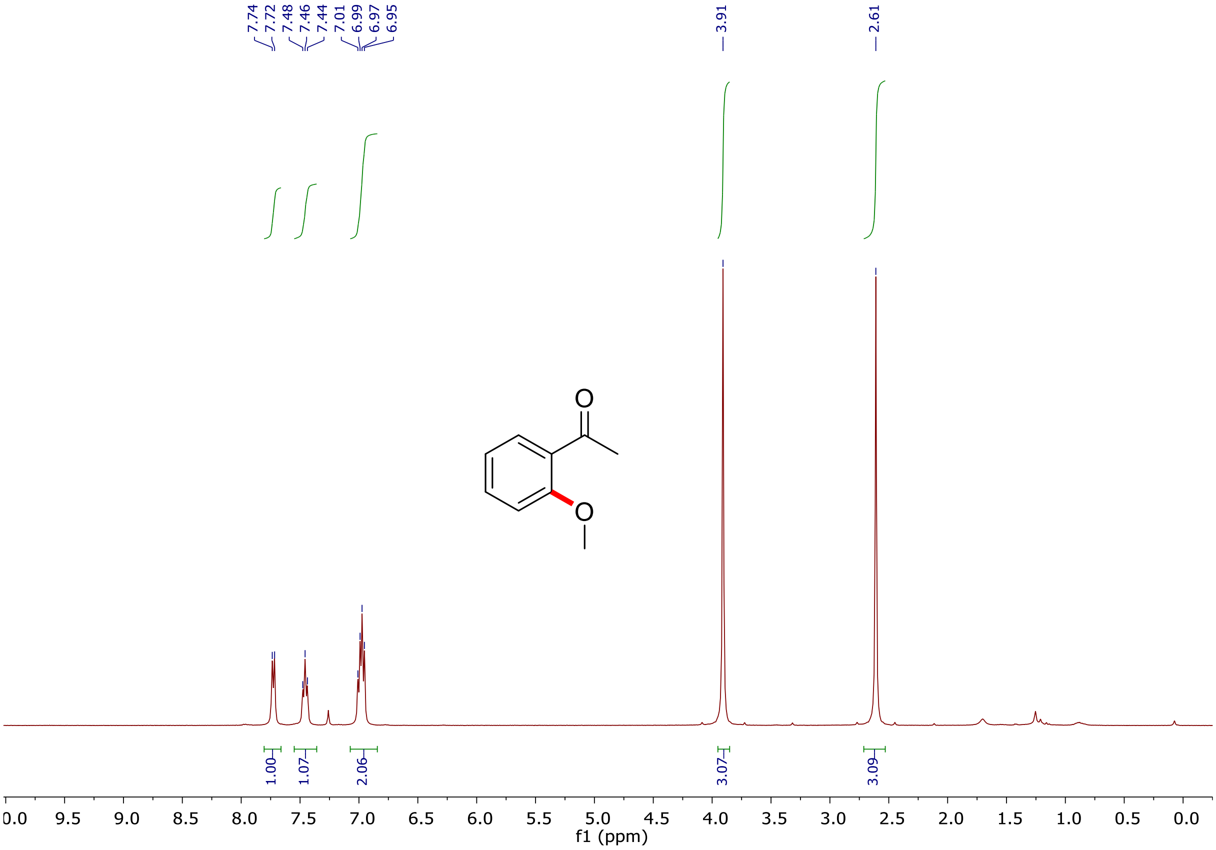
7.74
7.72
7.48
7.46
7.44
7.01
6.99
6.97
6.95
3.91
2.61
O
O
1.00
1.07
2.06
3.07
3.09
f1 (ppm)

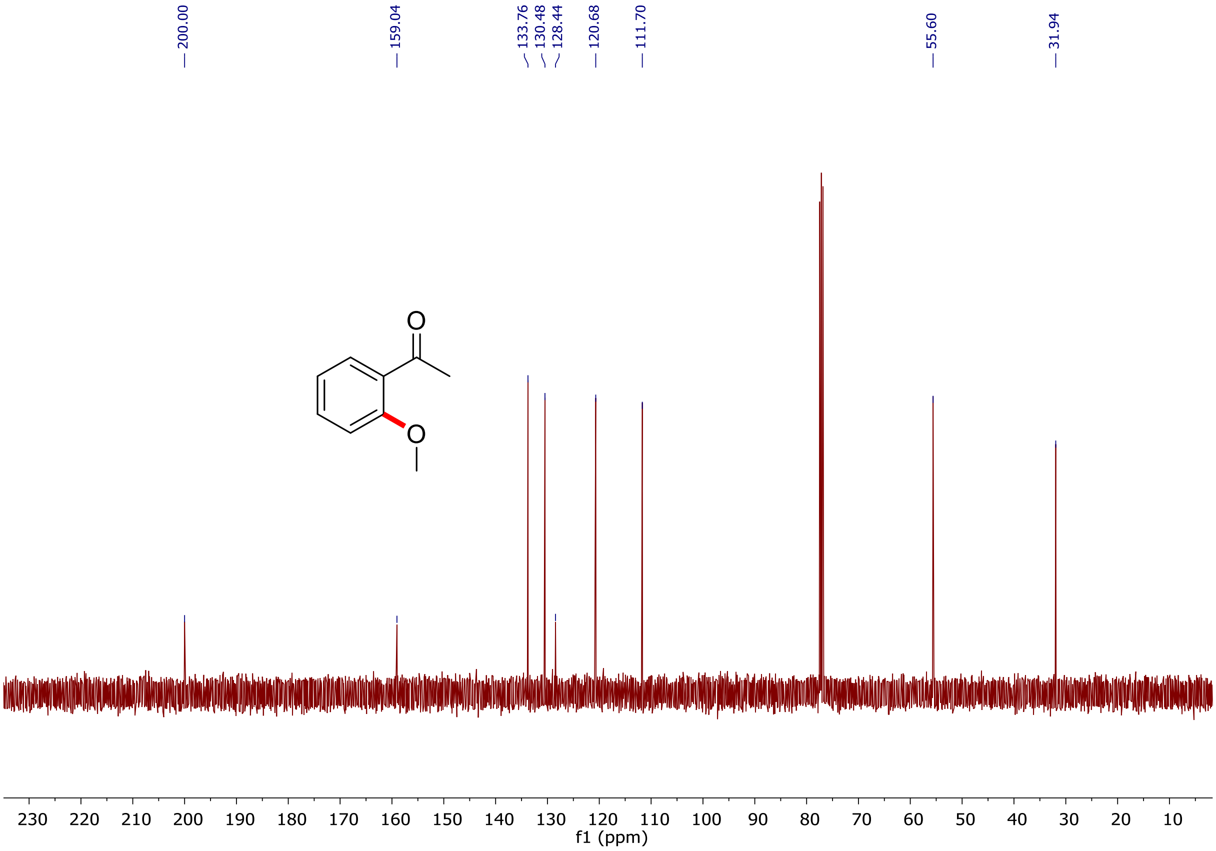
200.00
159.04
133.76
130.48
128.44
120.68
111.70
55.60
31.94
O
O
f1 (ppm)

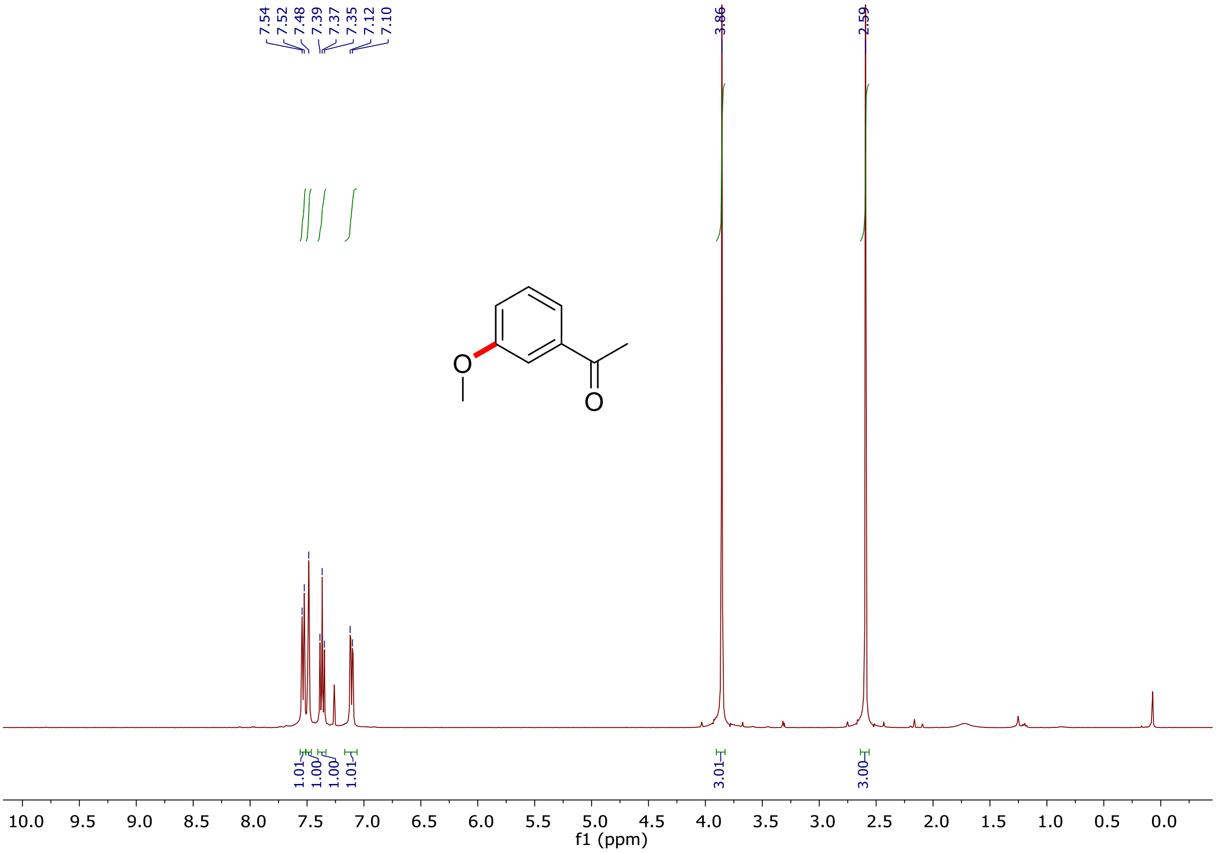
7.54
7.52
7.48
7.39
7.37
7.35
7.12
7.10
3.86
2.59
O
O
1.01
1.00
1.00
1.01
3.01
3.00
10.0 9.5 9.0 8.5 8.0 7.5 7.0 6.5 6.0 5.5 5.0 4.5 4.0 3.5 3.0 2.5 2.0 1.5 1.0 0.5 0.0
f1 (ppm)

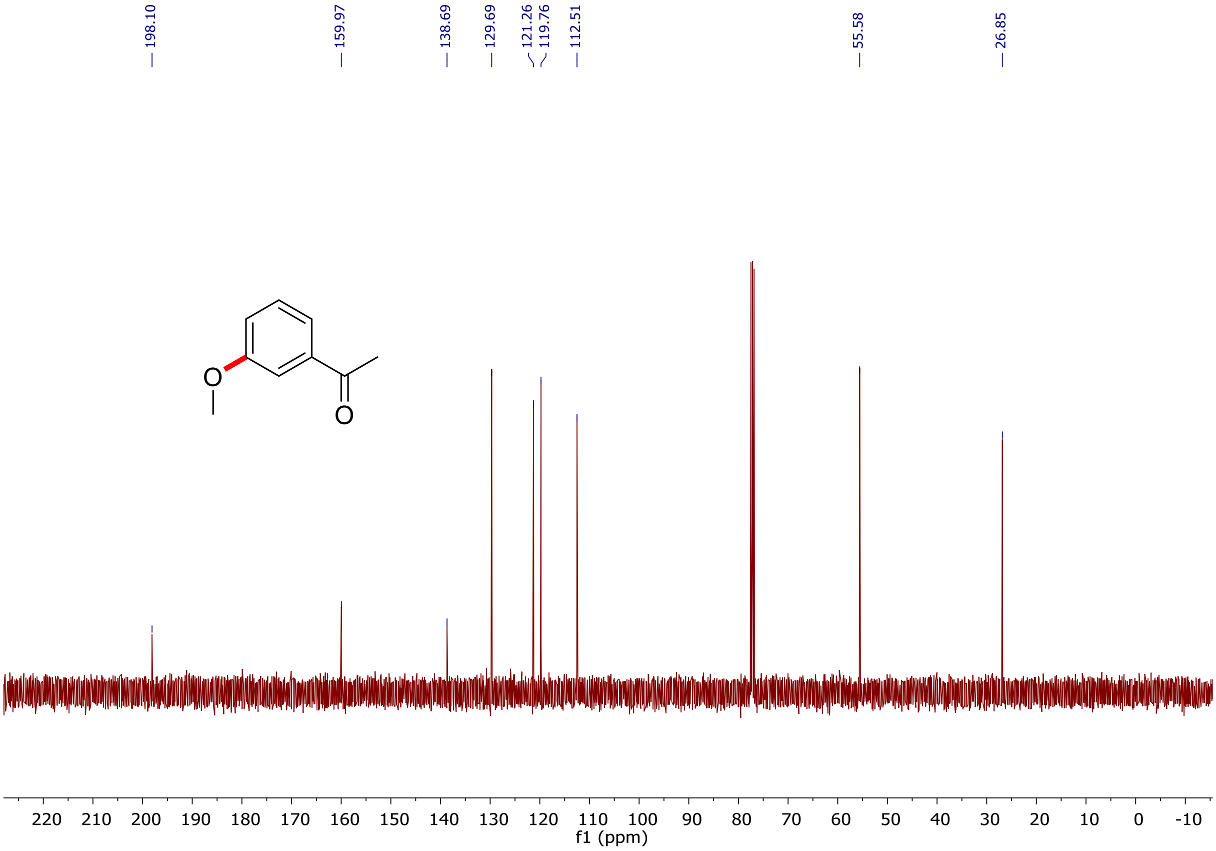
198.10
159.97
138.69
129.69
121.26
119.76
112.51
55.58
26.85
O
O
220 210 200 190 180 170 160 150 140 130 120 110 100 90 80 70 60 50 40 30 20 10 0 -10
f1 (ppm)

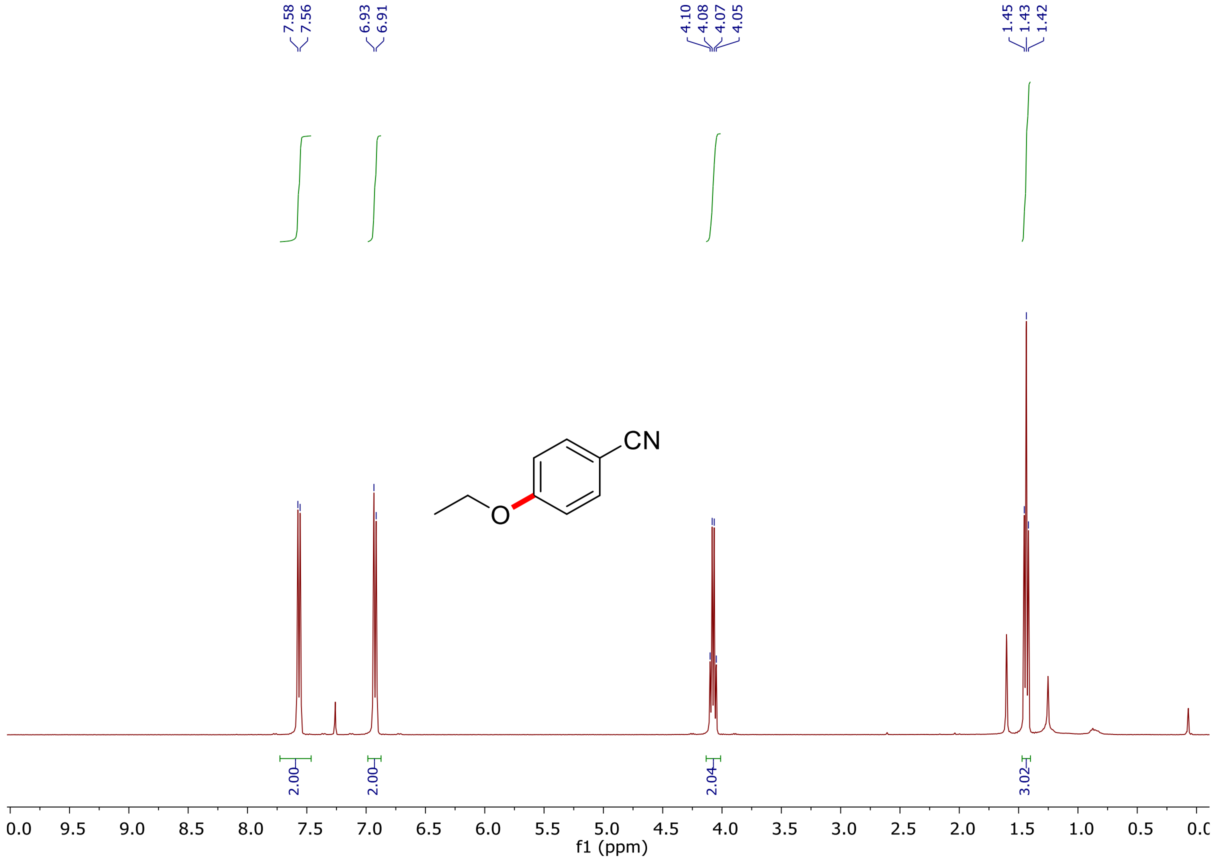
7.58
7.56
6.93
6.91
4.10
4.08
4.07
4.05
1.45
1.43
1.42
CN
O
2.00
2.00
2.04
3.02
f1 (ppm)

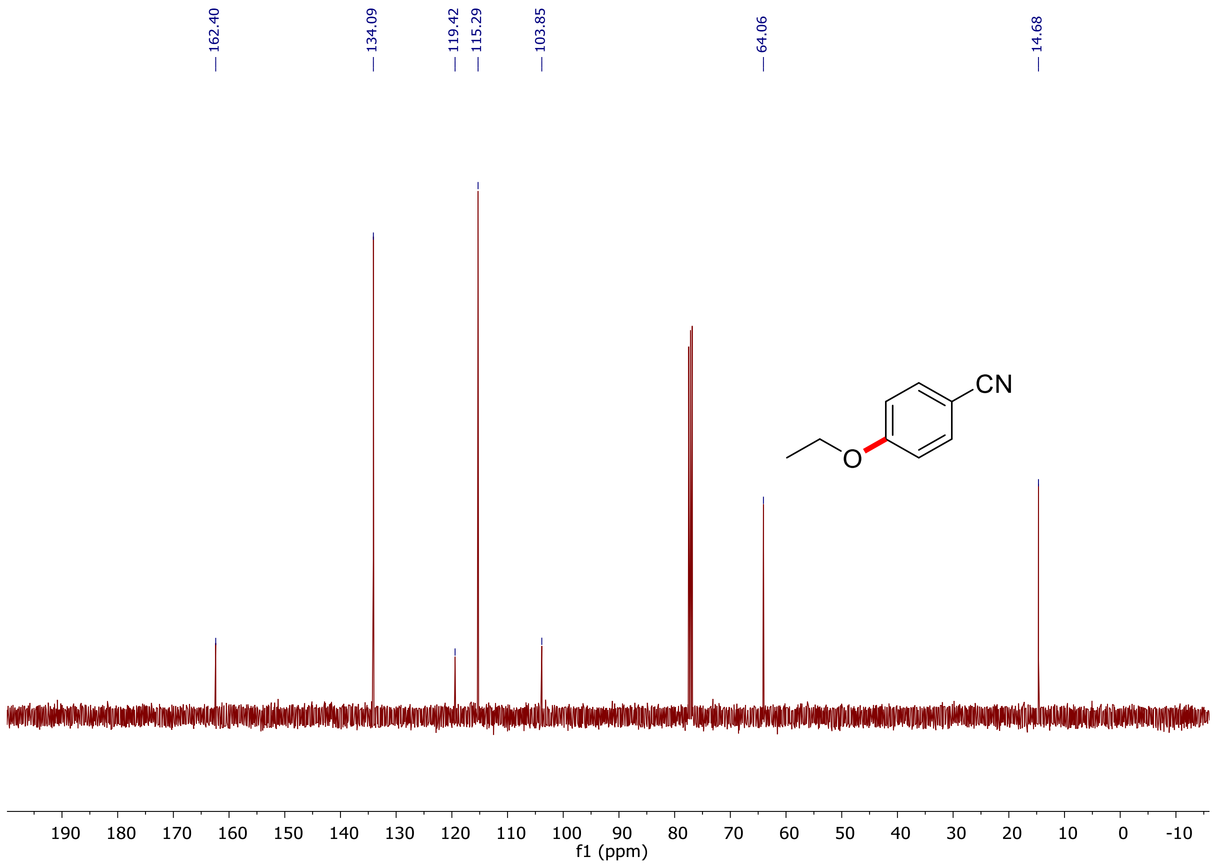
162.40
134.09
119.42
115.29
103.85
64.06
14.68
CN
O
f1 (ppm)

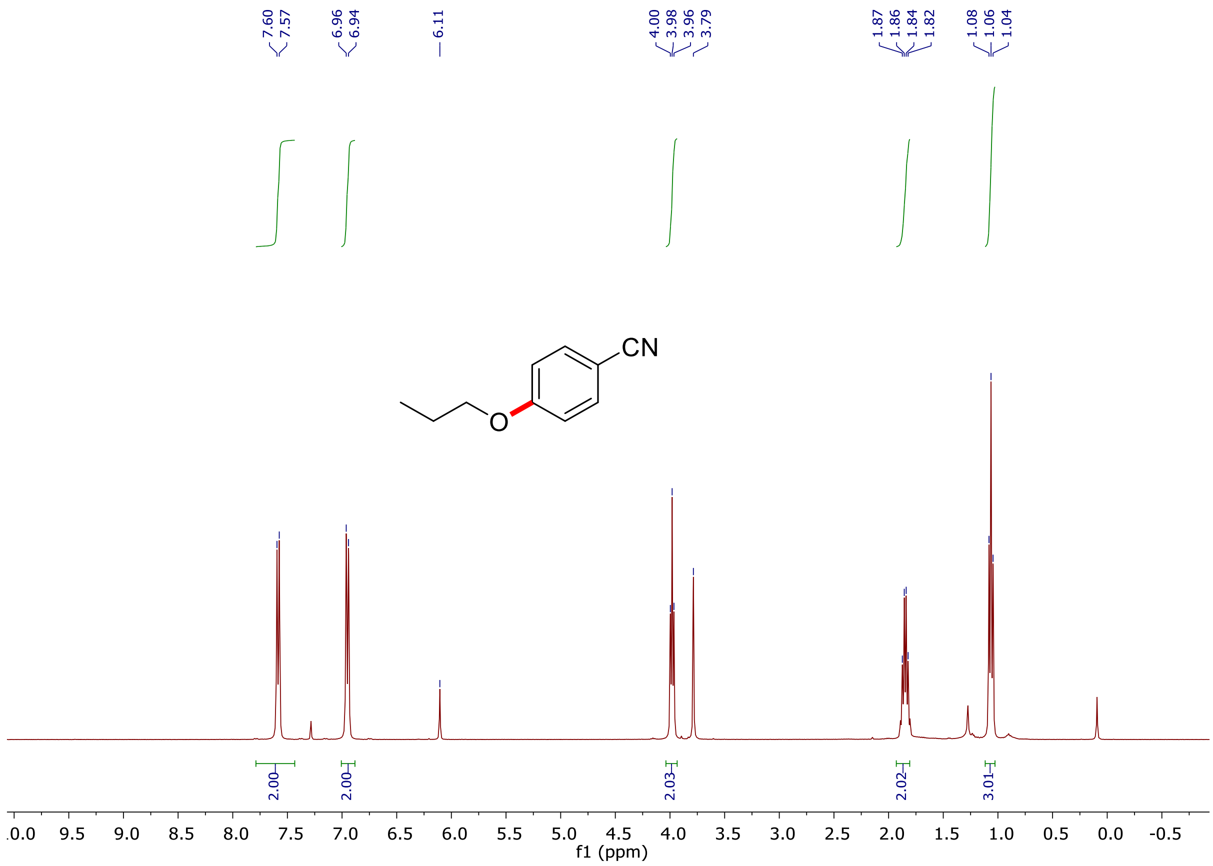
7.60
7.57
6.96
6.94
6.11
4.00
3.98
3.96
3.79
1.87
1.86
1.84
1.82
1.08
1.06
1.04
CN
O
2.00
2.00
2.03
2.02
3.01
f1 (ppm)

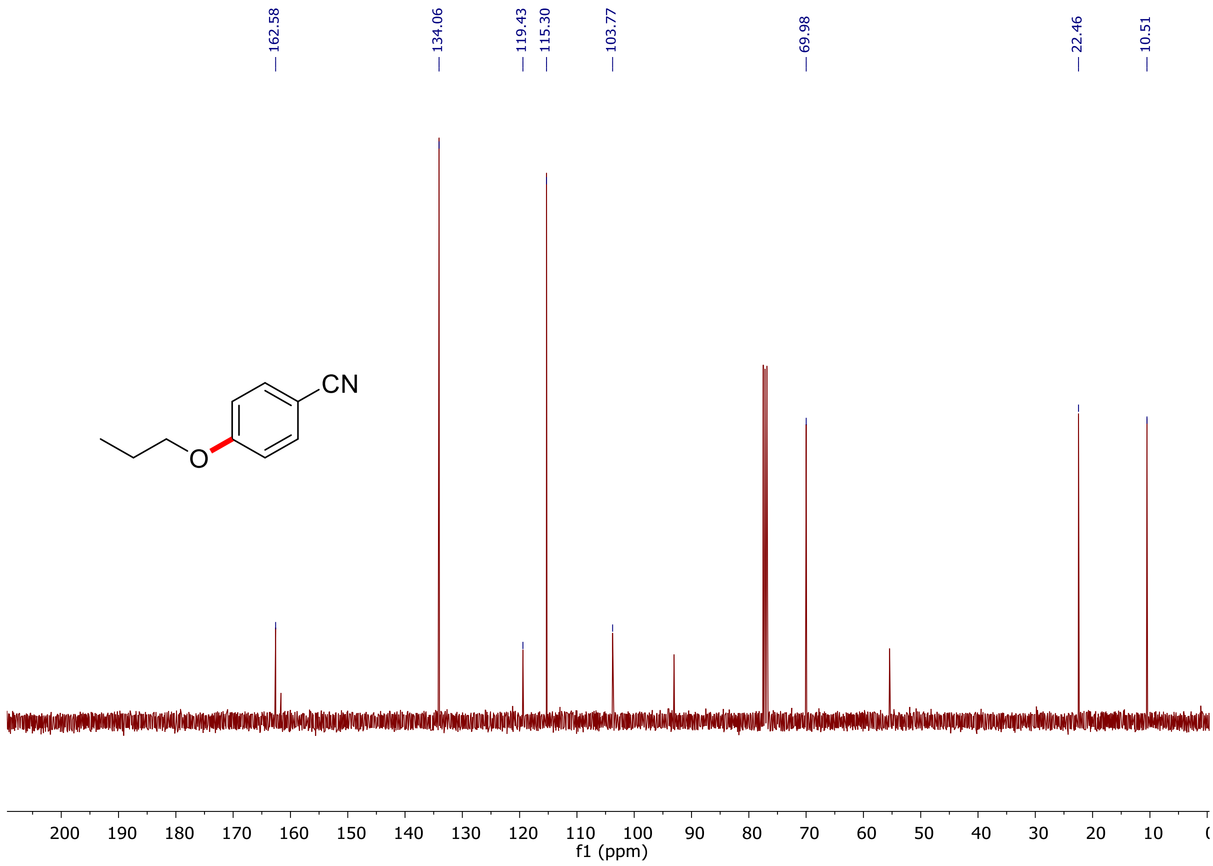
162.58
134.06
119.43
115.30
103.77
69.98
22.46
10.51
CN
O
f1 (ppm)

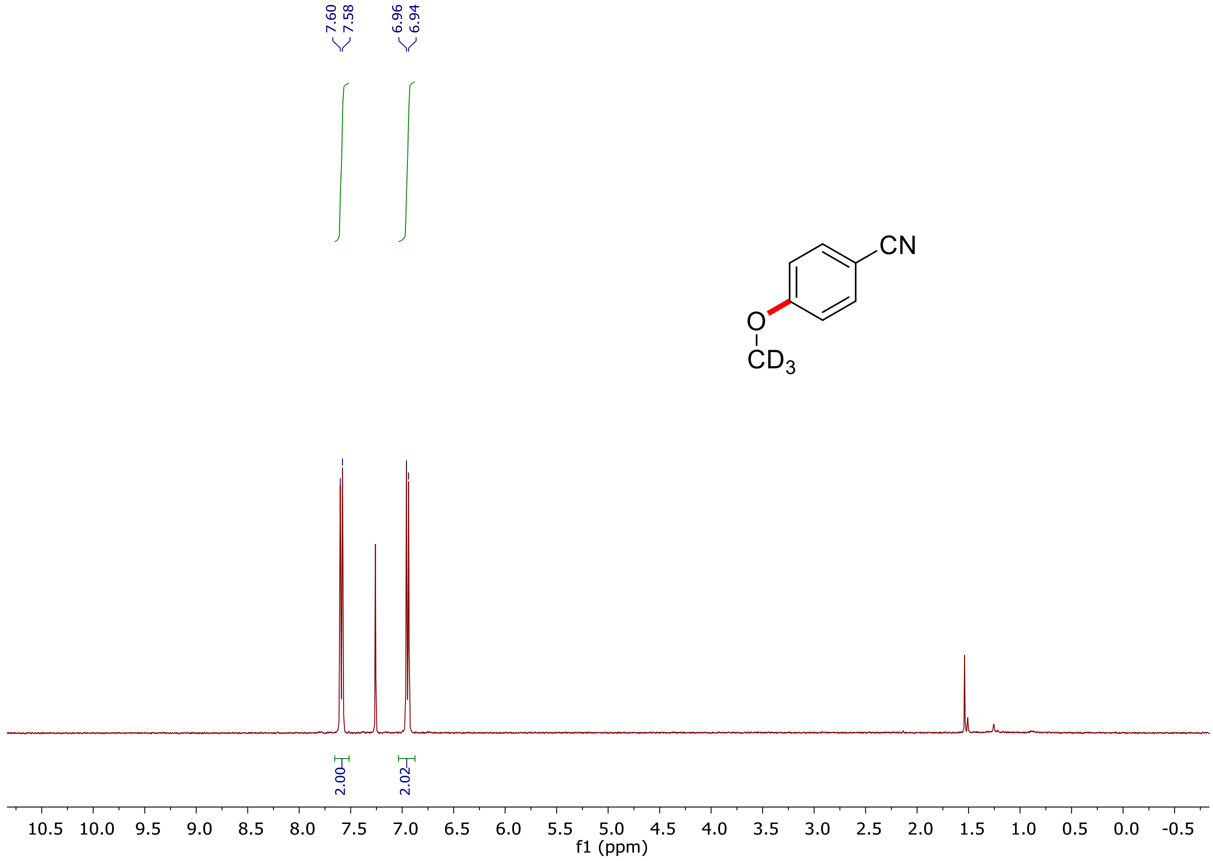
7.60
7.58
6.96
6.94
CN
O
CD3
2.00
2.02
10.5 10.0 9.5 9.0 8.5 8.0 7.5 7.0 6.5 6.0 5.5 5.0 4.5 4.0 3.5 3.0 2.5 2.0 1.5 1.0 0.5 0.0 -0.5
f1 (ppm)

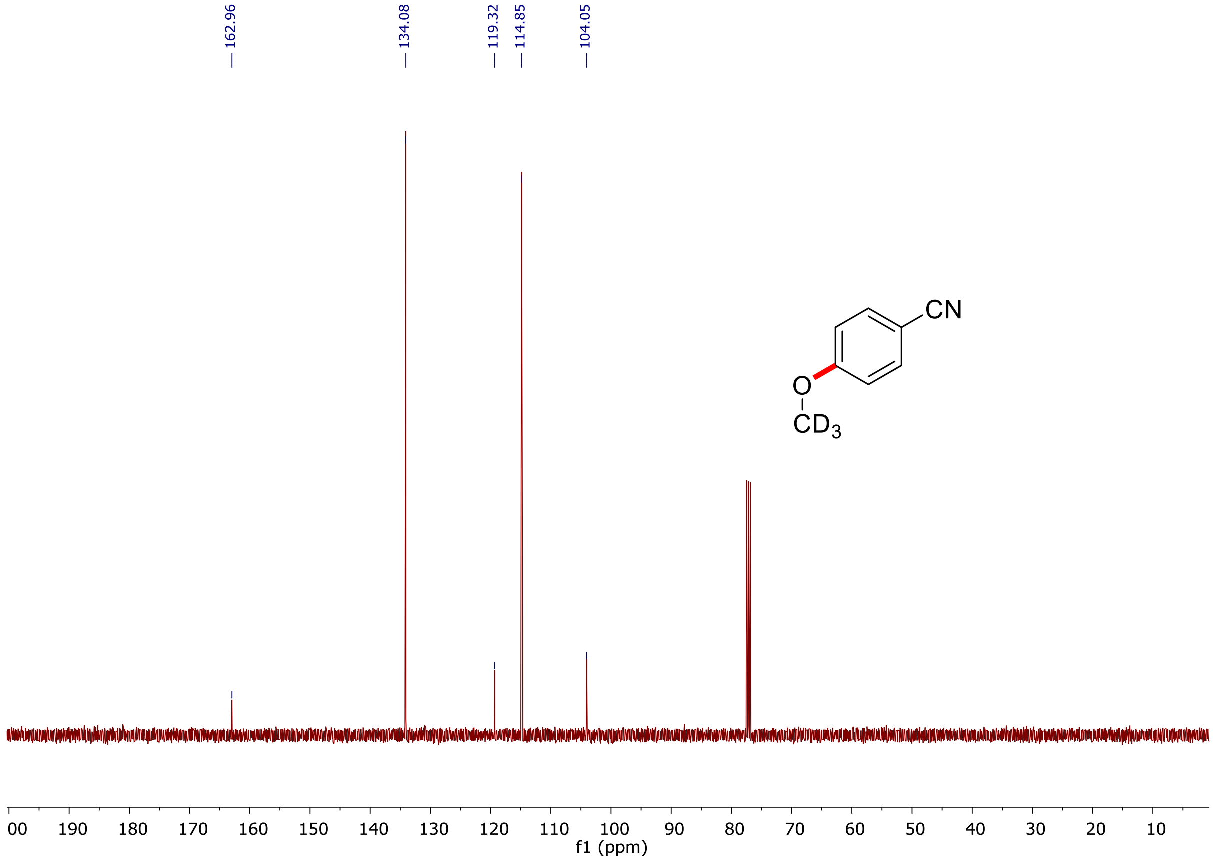
162.96
134.08
119.32
114.85
104.05
CN
O
CD3
00 190 180 170 160 150 140 130 120 110 100 90 80 70 60 50 40 30 20 10
f1 (ppm)

Energy profile and proposed catalytic cycle

Cartesian coordinates

DFT optimized structures

ArBr (substrate 3) (singlet, G = -2956.0141)

6	-1.033112000	1.262289000	0.000133000
6	-1.774705000	0.069287000	-0.000009000
6	0.357367000	1.240903000	0.000191000
6	-3.267424000	0.151470000	-0.000058000
1	0.922282000	2.166991000	0.000323000
6	-1.090692000	-1.156522000	-0.000125000
6	1.009791000	0.006725000	0.000049000
1	-1.634051000	-2.095506000	-0.000258000
6	0.302772000	-1.194358000	-0.000140000
1	0.824498000	-2.145204000	-0.000290000
1	-1.557594000	2.212239000	0.000223000
6	-4.075834000	-1.123422000	0.000275000
8	-3.826619000	1.247826000	-0.000341000
1	-5.139681000	-0.875621000	0.000345000
1	-3.844988000	-1.730955000	0.883635000
35	2.923389000	-0.037757000	-0.000008000
1	-3.845103000	-1.731291000	-0.882859000

ArOCH$_3$ (product 3a) (singlet, G = -499.4346)

6	-0.240529000	1.154668000	0.000056000
6	-1.109953000	0.051277000	-0.000060000
6	1.140321000	0.995456000	0.000081000
6	-2.575505000	0.286587000	-0.000039000
1	1.779861000	1.870231000	0.000162000
6	-0.546652000	-1.240707000	-0.000170000
6	1.683318000	-0.300273000	-0.000040000
1	-1.184533000	-2.118829000	-0.000271000
6	0.828417000	-1.416684000	-0.000199000
1	1.265757000	-2.410717000	-0.000312000
1	-0.662077000	2.155066000	0.000140000
6	-3.514508000	-0.899630000	0.000392000
8	-3.031344000	1.434115000	-0.000313000
1	-4.546559000	-0.541580000	0.000368000
1	-3.348576000	-1.527986000	0.883458000
8	3.013131000	-0.575358000	-0.000098000
1	-3.348728000	-1.528511000	-0.882318000
6	3.938834000	0.518757000	0.000192000
1	4.930861000	0.064061000	0.000136000
1	3.818648000	1.135949000	-0.896849000
1	3.818585000	1.135555000	0.897496000

Ni^{II}-mpg-CN$_x$ (singlet, G = -3677.7813)

1	-7.530275000	4.064849000	0.711427000
7	-6.619653000	4.498292000	0.623341000
6	-5.540553000	3.752805000	0.406007000
7	-5.734060000	2.410979000	0.313641000

6	-4.679642000	1.676510000	0.028789000
7	-3.438992000	2.306304000	-0.182591000
6	-3.292776000	3.687121000	0.038496000
7	-4.354273000	4.411808000	0.317772000
7	-2.490446000	0.262370000	-0.832593000
6	-3.619246000	-0.290052000	-0.387470000
7	-4.736313000	0.332389000	-0.015750000
6	-2.355906000	1.560307000	-0.619105000
7	-1.166223000	2.130963000	-0.762273000
6	-1.069187000	3.391249000	-0.336911000
7	-2.056444000	4.218804000	-0.002038000
7	0.222738000	3.905319000	-0.235675000
6	1.375735000	3.192083000	-0.085898000
7	2.523934000	3.866141000	-0.192329000
6	3.689874000	3.202373000	-0.103693000
7	3.646771000	1.810094000	0.101109000
7	1.285831000	1.859507000	0.201236000
6	2.440567000	1.238718000	0.255548000
6	4.803348000	1.001188000	0.104967000
7	4.865818000	3.771695000	-0.219554000
6	5.955027000	2.954287000	-0.155224000
7	7.140656000	3.533527000	-0.251269000
7	5.961528000	1.589860000	-0.013602000
7	4.661913000	-0.345150000	0.199725000
6	3.443965000	-0.843856000	0.316949000
7	2.339305000	-0.074805000	0.451705000
7	3.246250000	-2.202956000	0.315395000
1	4.076277000	-2.792016000	0.266029000
7	0.911745000	-2.082872000	0.224583000
6	2.028300000	-2.848005000	0.249041000
7	2.013082000	-4.167976000	0.203789000
6	0.819626000	-4.799575000	0.091028000
7	0.711981000	-6.099756000	0.042807000
6	-0.551911000	-6.613499000	-0.079121000
7	-1.717057000	-5.915512000	-0.161896000
7	-0.642109000	-7.933936000	-0.121172000
7	-0.355421000	-4.025069000	0.022391000
6	-0.279975000	-2.677878000	0.106953000
6	-1.631831000	-4.605909000	-0.115381000
7	-2.697818000	-3.793948000	-0.215993000
6	-2.507215000	-2.473818000	-0.149682000
7	-1.308626000	-1.870675000	0.074700000
7	-3.613552000	-1.679780000	-0.288243000
1	-6.534063000	5.502449000	0.718909000
1	0.317015000	4.917980000	-0.210465000
1	7.211813000	4.538000000	-0.365802000
1	7.986388000	2.976254000	-0.214229000
1	-4.514824000	-2.146304000	-0.218761000
1	0.192085000	-8.505800000	-0.059336000
1	-1.547284000	-8.381410000	-0.205761000
28	0.570348000	-0.293129000	0.178776000

Ni^{I}-mpg-CN_x (doublet, G = -3677.9507)

1	-0.876946000	-8.482202000	0.254278000
7	-0.001956000	-7.973660000	0.235907000
6	-0.001591000	-6.642978000	0.201568000
7	-1.215323000	-6.035155000	0.181288000
6	-1.222212000	-4.717729000	0.158746000
7	-0.000972000	-4.020851000	0.165049000
6	1.219937000	-4.718301000	0.159337000
7	1.212419000	-6.035737000	0.182007000
7	-1.142263000	-1.962114000	0.179868000
6	-2.267990000	-2.689326000	0.092447000

7	-2.367071000	-4.021014000	0.106821000
6	-0.000660000	-2.636725000	0.182862000
7	1.141300000	-1.962658000	0.180687000
6	2.266668000	-2.690394000	0.093383000
7	2.365127000	-4.022144000	0.107832000
7	3.475937000	-2.015039000	0.007354000
6	3.620153000	-0.657418000	-0.179013000
7	4.737735000	-0.074964000	0.263502000
6	4.762270000	1.269849000	0.251216000
7	3.577928000	1.959341000	-0.084787000
7	2.583607000	-0.046768000	-0.770042000
6	2.512999000	1.248888000	-0.559091000
6	3.466436000	3.352485000	0.084271000
7	5.821097000	1.967801000	0.599599000
6	5.677329000	3.321520000	0.644550000
7	6.769167000	4.021528000	0.943006000
7	4.537070000	4.032257000	0.443585000
7	2.270551000	3.928340000	-0.101875000
6	1.245607000	3.142891000	-0.447631000
7	1.327319000	1.834024000	-0.762013000
7	0.000914000	3.738160000	-0.447911000
1	0.001222000	4.719673000	-0.181005000
7	-1.326451000	1.834839000	-0.761985000
6	-1.244126000	3.143613000	-0.447537000
7	-2.268689000	3.929591000	-0.101833000
6	-3.464785000	3.354207000	0.084547000
7	-4.535097000	4.034428000	0.443955000
6	-5.675649000	3.324172000	0.645010000
7	-5.820028000	1.970497000	0.599916000
7	-6.767136000	4.024620000	0.943648000
7	-3.576882000	1.961106000	-0.084488000
6	-2.512385000	1.250215000	-0.559181000
6	-4.761537000	1.272147000	0.251324000
7	-4.737644000	-0.072697000	0.263299000
6	-3.620432000	-0.655619000	-0.179480000
7	-2.583658000	-0.045327000	-0.770541000
7	-3.476901000	-2.013351000	0.006555000
1	0.872694000	-8.482762000	0.254795000
1	4.308443000	-2.554767000	0.226878000
1	7.645951000	3.547761000	1.119193000
1	6.724913000	5.030560000	1.007932000
1	-4.309789000	-2.552613000	0.225784000
1	-6.722418000	5.033608000	1.009000000
1	-7.644030000	3.551187000	1.120186000
28	-0.000033000	0.429114000	-0.774280000

Ni^{0}-mpg-CN_x (singlet, G = -3678.0524)

1	-7.186246000	4.654955000	-0.344753000
7	-7.130781000	3.658672000	-0.510681000
6	-5.975807000	3.011885000	-0.339619000
7	-4.915791000	3.763918000	0.052161000
6	-3.780483000	3.123748000	0.267677000
7	-3.735924000	1.729023000	0.097777000
6	-4.857053000	1.023130000	-0.377486000
7	-5.986461000	1.679418000	-0.587191000
7	-1.533987000	1.687752000	0.925895000
6	-1.584569000	3.013761000	0.871717000
7	-2.660721000	3.774000000	0.616884000
6	-2.589447000	1.028208000	0.458454000
7	-2.532922000	-0.279812000	0.295238000
6	-3.566636000	-0.860253000	-0.338561000
7	-4.745762000	-0.286025000	-0.631213000
7	-3.399300000	-2.172485000	-0.690909000

6	-2.143936000	-2.819246000	-0.660256000
7	-2.124936000	-4.034044000	-0.075146000
6	-0.912635000	-4.587580000	0.085000000
7	0.231477000	-3.849584000	-0.220426000
7	-1.146152000	-2.158955000	-1.204745000
6	0.104809000	-2.553425000	-0.812900000
6	1.507552000	-4.323972000	0.110832000
7	-0.782112000	-5.814933000	0.571633000
6	0.486616000	-6.254641000	0.774390000
7	0.606940000	-7.515053000	1.220292000
7	1.629465000	-5.563054000	0.596721000
7	2.559065000	-3.524926000	-0.037384000
6	2.316029000	-2.298974000	-0.595675000
7	1.220383000	-2.024323000	-1.445730000
7	3.491232000	-1.495178000	-0.808601000
1	4.331098000	-1.911402000	-1.200732000
7	2.427017000	0.181063000	0.320149000
6	3.528239000	-0.210437000	-0.391431000
7	4.565990000	0.571644000	-0.692046000
6	4.460482000	1.874063000	-0.397718000
7	5.446634000	2.720314000	-0.618313000
6	5.207204000	4.029857000	-0.347976000
7	4.035415000	4.579291000	0.058698000
7	6.229390000	4.866498000	-0.523129000
7	3.246021000	2.365316000	0.124599000
6	2.243369000	1.491510000	0.492964000
6	3.035871000	3.748938000	0.288362000
7	1.816351000	4.185086000	0.632383000
6	0.890539000	3.250626000	0.893384000
7	1.094161000	1.931303000	0.966023000
7	-0.399598000	3.721559000	1.061454000
1	-7.956335000	3.154459000	-0.806302000
1	-4.233294000	-2.729811000	-0.845262000
1	-0.217912000	-8.066705000	1.411448000
1	1.520589000	-7.897180000	1.421897000
1	-0.494503000	4.730089000	0.979580000
1	7.125606000	4.515946000	-0.836149000
1	6.113363000	5.856518000	-0.348595000
28	1.008611000	-1.118053000	0.150756000

Ni^{III}-ArBr-mpg-CN$_x$ (doublet, G = -6634.0172)

1	8.602233000	1.325789000	2.317325000
7	8.186756000	0.425374000	2.115792000
6	7.002338000	0.354300000	1.510754000
7	6.401135000	1.533632000	1.211369000
6	5.242996000	1.469716000	0.583917000
7	4.710108000	0.214349000	0.239311000
6	5.358211000	-0.966457000	0.644003000
7	6.514020000	-0.889713000	1.274324000
7	2.941087000	1.254765000	-0.909917000
6	3.402213000	2.391365000	-0.384273000
7	4.533123000	2.573910000	0.300671000
6	3.551119000	0.139940000	-0.529100000
7	3.047610000	-1.045013000	-0.848620000
6	3.616442000	-2.106366000	-0.273339000
7	4.759660000	-2.146116000	0.414183000
7	2.954242000	-3.320426000	-0.427596000
6	1.614790000	-3.467067000	-0.699173000
7	1.232527000	-4.611331000	-1.267241000
6	-0.048991000	-4.702625000	-1.652211000
7	-0.885479000	-3.578456000	-1.502340000
7	0.811424000	-2.441061000	-0.348910000
6	-0.413919000	-2.488582000	-0.804640000

6	-2.195717000	-3.586991000	-2.015059000
7	-0.536255000	-5.793978000	-2.204805000
6	-1.830635000	-5.756161000	-2.608424000
7	-2.333275000	-6.873283000	-3.121444000
7	-2.668364000	-4.681739000	-2.558704000
7	-2.905558000	-2.443636000	-1.977517000
6	-2.374138000	-1.412658000	-1.338864000
7	-1.232497000	-1.432594000	-0.620009000
7	-3.047507000	-0.209284000	-1.453843000
1	-3.909605000	-0.265188000	-1.992872000
7	-1.348541000	1.217474000	-0.706304000
6	-2.487035000	1.054537000	-1.411506000
7	-3.114512000	1.998249000	-2.095818000
6	-2.520982000	3.203884000	-2.184284000
7	-3.102763000	4.223653000	-2.765709000
6	-2.383987000	5.379959000	-2.845988000
7	-1.101159000	5.566001000	-2.446936000
7	-3.005746000	6.422578000	-3.384339000
7	-1.218475000	3.349982000	-1.672914000
6	-0.640211000	2.341414000	-0.935378000
6	-0.502862000	4.549265000	-1.861382000
7	0.780383000	4.602842000	-1.475035000
6	1.276344000	3.525188000	-0.865910000
7	0.581102000	2.436399000	-0.475459000
7	2.623164000	3.525680000	-0.592502000
1	8.684820000	-0.420013000	2.363182000
1	3.515650000	-4.166615000	-0.384554000
1	-1.759841000	-7.704785000	-3.193645000
1	-3.291930000	-6.899304000	-3.446308000
1	3.093510000	4.426487000	-0.578349000
1	-3.962047000	6.335608000	-3.705347000
1	-2.527232000	7.309931000	-3.478943000
28	-0.609197000	-0.028134000	0.652242000
6	-3.834811000	-1.235446000	2.960107000
6	-4.475009000	-0.028229000	3.281912000
6	-2.626922000	-1.243700000	2.263698000
6	-5.761779000	-0.079834000	4.043676000
1	-2.131494000	-2.184675000	2.049398000
6	-3.880177000	1.183151000	2.898967000
6	-2.094639000	-0.023136000	1.858748000
1	-4.340491000	2.134027000	3.146886000
6	-2.671515000	1.194801000	2.197789000
1	-2.214191000	2.141659000	1.931901000
1	-4.280417000	-2.174950000	3.271874000
6	-6.433209000	1.210838000	4.445997000
8	-6.261586000	-1.164549000	4.338674000
1	-7.344648000	0.983965000	5.003544000
1	-6.688065000	1.808286000	3.562341000
35	0.660281000	0.030863000	2.528981000
1	-5.766864000	1.819294000	5.069022000

Ni^{III}-$ArOCH_3$-mpg-CN_x (doublet, G = -4177.3950)

1	8.583591000	1.017777000	2.634979000
7	8.143644000	0.134397000	2.411286000
6	6.979860000	0.109944000	1.763643000
7	6.430308000	1.311967000	1.455430000
6	5.290603000	1.294316000	0.791881000
7	4.723419000	0.060574000	0.423977000
6	5.318619000	-1.145929000	0.834688000
7	6.458167000	-1.114430000	1.497812000
7	3.016109000	1.170983000	-0.751925000
6	3.507853000	2.288632000	-0.213144000
7	4.631468000	2.425739000	0.494725000

6	3.579552000	0.033381000	-0.367925000
7	3.039843000	-1.129874000	-0.707731000
6	3.558133000	-2.215976000	-0.130525000
7	4.684849000	-2.302053000	0.580135000
7	2.852819000	-3.400940000	-0.308951000
6	1.517595000	-3.492565000	-0.628463000
7	1.119322000	-4.607665000	-1.240221000
6	-0.149127000	-4.645184000	-1.674490000
7	-0.953887000	-3.498528000	-1.522072000
7	0.735996000	-2.450815000	-0.274641000
6	-0.475282000	-2.441740000	-0.776944000
6	-2.246169000	-3.453629000	-2.078645000
7	-0.648429000	-5.704612000	-2.275763000
6	-1.924975000	-5.614776000	-2.725868000
7	-2.436200000	-6.697784000	-3.300612000
7	-2.731960000	-4.518516000	-2.670135000
7	-2.924027000	-2.292686000	-2.031287000
6	-2.381424000	-1.294210000	-1.349866000
7	-1.266379000	-1.365343000	-0.596095000
7	-3.014050000	-0.068298000	-1.455837000
1	-3.857772000	-0.084220000	-2.025593000
7	-1.284435000	1.284445000	-0.644448000
6	-2.408298000	1.174273000	-1.379587000
7	-2.987371000	2.146549000	-2.069311000
6	-2.344346000	3.326087000	-2.139700000
7	-2.869962000	4.369035000	-2.736306000
6	-2.100850000	5.491565000	-2.806197000
7	-0.822626000	5.625514000	-2.373140000
7	-2.657390000	6.555200000	-3.375427000
7	-1.049196000	3.417293000	-1.597013000
6	-0.529533000	2.382055000	-0.847375000
6	-0.282270000	4.587027000	-1.769936000
7	0.992367000	4.593395000	-1.352825000
6	1.431066000	3.496843000	-0.734456000
7	0.686630000	2.436439000	-0.360947000
7	2.772039000	3.448776000	-0.428502000
1	8.603549000	-0.730178000	2.666135000
1	3.379163000	-4.269263000	-0.264550000
1	-1.883246000	-7.542276000	-3.380702000
1	-3.380888000	-6.684912000	-3.664730000
1	3.272531000	4.332803000	-0.398794000
1	-3.606801000	6.509033000	-3.724028000
1	-2.135726000	7.418496000	-3.464022000
28	-0.629377000	-0.027375000	0.750694000
6	-3.769833000	-1.259993000	3.137443000
6	-4.395875000	-0.056550000	3.500471000
6	-2.632485000	-1.260166000	2.331143000
6	-5.614069000	-0.112352000	4.365041000
1	-2.159705000	-2.201509000	2.068683000
6	-3.857135000	1.156232000	3.044599000
6	-2.132436000	-0.040533000	1.876723000
1	-4.314822000	2.102834000	3.313414000
6	-2.716668000	1.170475000	2.237265000
1	-2.310893000	2.120120000	1.903201000
1	-4.178760000	-2.200315000	3.494194000
6	-6.278364000	1.176292000	4.787364000
8	-6.067229000	-1.197395000	4.728551000
1	-7.150307000	0.946883000	5.403967000
1	-6.594363000	1.757273000	3.912819000
8	0.475530000	0.102735000	2.102864000
6	0.391426000	0.263583000	3.503268000
1	1.416656000	0.320755000	3.895133000
1	-0.131202000	1.188880000	3.779348000
1	-0.112829000	-0.583908000	3.986081000
1	-5.582905000	1.801557000	5.360020000

ONIOM DFT-MM optimised structures

Ni^{II}-mpg-CN$_x$ (singlet, G = -3611.2776)

1	-1.779508000	8.326198000	-2.007235000
7	-2.395870000	7.523374000	-2.028397000
6	-1.910342000	6.300263000	-1.839468000
7	-0.572412000	6.201194000	-1.623566000
6	-0.099055000	5.000271000	-1.366455000
7	-0.985810000	3.909060000	-1.307694000
6	-2.339745000	4.070495000	-1.650799000
7	-2.802667000	5.275911000	-1.901005000
7	0.749922000	2.513384000	-0.573393000
6	1.560910000	3.526296000	-0.879665000
7	1.217801000	4.770190000	-1.208949000
6	-0.521614000	2.668305000	-0.901294000
7	-1.333820000	1.618630000	-0.901403000
6	-2.540712000	1.815431000	-1.433714000
7	-3.113652000	2.973505000	-1.753477000
7	-3.295184000	0.669340000	-1.680310000
6	-2.820888000	-0.596383000	-1.864607000
7	-3.721988000	-1.581347000	-1.906270000
6	-3.304335000	-2.853007000	-2.035002000
7	-1.910474000	-3.087680000	-2.127745000
7	-1.453171000	-0.814191000	-1.968201000
6	-1.059563000	-2.040885000	-2.101183000
6	-1.366663000	-4.388951000	-2.154252000
7	-4.109886000	-3.887819000	-2.060925000
6	-3.529316000	-5.118380000	-2.146826000
7	-4.344548000	-6.159354000	-2.194103000
7	-2.187673000	-5.402789000	-2.177707000
7	-0.006102000	-4.550705000	-2.115430000
6	0.786128000	-3.498983000	-2.074394000
7	0.231858000	-2.240327000	-2.121204000
7	2.132953000	-3.639766000	-1.944407000
1	2.535304000	-4.604844000	-1.916864000
7	2.474408000	-1.277656000	-1.776177000
6	2.970441000	-2.574256000	-1.777997000
7	4.255191000	-2.772112000	-1.585904000
6	5.095502000	-1.722712000	-1.349309000
7	6.379717000	-1.894239000	-1.187592000
6	7.126185000	-0.776672000	-0.922701000
7	6.675695000	0.501739000	-0.802700000
7	8.427046000	-0.968902000	-0.766897000
7	4.571191000	-0.418689000	-1.277256000
6	3.248321000	-0.240430000	-1.494886000
6	5.386851000	0.696739000	-0.961779000
7	4.803395000	1.901357000	-0.841233000
6	3.484712000	1.996834000	-1.029207000
7	2.684816000	0.915568000	-1.383055000
7	2.923265000	3.234722000	-0.866325000
1	-3.382504000	7.656942000	-2.211450000
1	-4.298631000	0.791830000	-1.794864000
1	-5.348686000	-6.024720000	-2.167292000
1	-3.970425000	-7.099359000	-2.254224000
1	3.568174000	4.020308000	-0.824777000
1	8.820101000	-1.899179000	-0.849533000
1	9.039406000	-0.185111000	-0.573495000
28	0.846815000	-0.630376000	-1.876277000
6	-2.159627000	-2.318185000	1.833544000
1	-5.269023000	7.101058000	1.754887000
7	-4.258616000	7.038267000	1.752040000
6	-3.660702000	5.856468000	1.636803000

7	-4.470442000	4.770727000	1.527416000
6	-3.883203000	3.607374000	1.342659000
7	-2.480189000	3.552030000	1.247777000
6	-1.703312000	4.700533000	1.480894000
7	-2.300995000	5.858475000	1.659442000
7	-2.588429000	1.276665000	0.690215000
6	-3.875622000	1.356561000	1.028217000
7	-4.578690000	2.455953000	1.293672000
6	-1.861166000	2.357881000	0.914706000
7	-0.536724000	2.278406000	0.883011000
6	0.122368000	3.354365000	1.314959000
7	-0.365409000	4.569166000	1.555756000
7	1.488660000	3.187289000	1.536178000
6	2.136636000	2.014884000	1.793340000
7	3.471919000	2.047570000	1.794296000
6	4.160085000	0.909463000	1.991776000
7	3.432817000	-0.279285000	2.192333000
7	1.390732000	0.904684000	2.071472000
6	2.092696000	-0.191977000	2.233706000
6	4.049571000	-1.543802000	2.304592000
7	5.469687000	0.836672000	1.985984000
6	6.018688000	-0.400120000	2.152167000
7	7.339908000	-0.467977000	2.167002000
7	5.353266000	-1.591229000	2.294435000
7	3.267775000	-2.650024000	2.386383000
6	1.955585000	-2.495148000	2.389573000
7	1.353519000	-1.284180000	2.420183000
7	1.126423000	-3.589847000	2.370216000
1	1.569164000	-4.507196000	2.398879000
7	-0.842895000	-2.365431000	2.061682000
6	-0.241541000	-3.571440000	2.190699000
7	-0.889167000	-4.721693000	2.144864000
6	-2.225494000	-4.705416000	1.921430000
7	-2.943867000	-5.794011000	1.863997000
6	-4.283873000	-5.641083000	1.624798000
7	-4.955281000	-4.473133000	1.433213000
7	-4.997332000	-6.755517000	1.575148000
7	-2.869691000	-3.465070000	1.742959000
6	-4.251757000	-3.365583000	1.486848000
7	-4.780185000	-2.146388000	1.286718000
6	-3.981336000	-1.079436000	1.369898000
7	-2.663393000	-1.118004000	1.704964000
7	-4.550510000	0.141762000	1.128224000
1	-3.706420000	7.880490000	1.854697000
1	2.058273000	4.029562000	1.569595000
1	7.896811000	0.371882000	2.059133000
1	7.804662000	-1.361117000	2.283148000
1	-5.567238000	0.170241000	1.113443000
1	-4.551711000	-7.654755000	1.714904000
1	-5.995691000	-6.715509000	1.406598000

Ni^{I}-mpg-CN_x (doublet, G = -3610.6496)

1	-1.993222000	8.278269000	-2.014499000
7	-2.588747000	7.459847000	-2.034754000
6	-2.071960000	6.249837000	-1.844387000
7	-0.731941000	6.185434000	-1.628319000
6	-0.227896000	4.997371000	-1.369796000
7	-1.086320000	3.883801000	-1.309804000
6	-2.443932000	4.009990000	-1.653149000
7	-2.937662000	5.202821000	-1.904771000
7	0.684656000	2.534043000	-0.573817000
6	1.469366000	3.567105000	-0.881224000
7	1.094427000	4.801390000	-1.211972000

6	-0.590419000	2.655856000	-0.901947000
7	-1.375382000	1.585656000	-0.900870000
6	-2.586895000	1.750762000	-1.433471000
7	-3.189386000	2.893356000	-1.754584000
7	-3.311678000	0.585356000	-1.678717000
6	-2.804828000	-0.668020000	-1.861593000
7	-3.680516000	-1.675778000	-1.902099000
6	-3.230162000	-2.936445000	-2.029462000
7	-1.825381000	-3.131269000	-2.134551000
7	-1.399019000	-0.852651000	-2.032315000
6	-0.986249000	-2.064427000	-2.161997000
6	-1.253814000	-4.422152000	-2.146786000
7	-4.008942000	-3.991658000	-2.054050000
6	-3.396936000	-5.206987000	-2.138622000
7	-4.185143000	-6.268624000	-2.184663000
7	-2.048397000	-5.456884000	-2.168951000
7	0.108187000	-4.560032000	-2.094865000
6	0.884500000	-3.498917000	-2.101911000
7	0.314631000	-2.259344000	-2.274260000
7	2.231042000	-3.615387000	-1.918573000
1	2.646559000	-4.570584000	-1.825134000
7	2.500227000	-1.220906000	-1.838360000
6	3.044230000	-2.528615000	-1.783322000
7	4.331428000	-2.673815000	-1.568518000
6	5.137642000	-1.589860000	-1.344656000
7	6.425903000	-1.728166000	-1.182694000
6	7.143385000	-0.591468000	-0.919051000
7	6.660186000	0.675088000	-0.800572000
7	8.448748000	-0.750022000	-0.762983000
7	4.572301000	-0.299714000	-1.274359000
6	3.239193000	-0.156659000	-1.507772000
6	5.366735000	0.836695000	-0.959935000
7	4.752558000	2.026093000	-0.840808000
6	3.431808000	2.087388000	-1.028832000
7	2.643828000	0.965821000	-1.405712000
7	2.838790000	3.310669000	-0.867506000
1	-3.578475000	7.567803000	-2.218000000
1	-4.317906000	0.681900000	-1.793475000
1	-5.192411000	-6.159808000	-2.158047000
1	-3.786983000	-7.198773000	-2.243679000
1	3.463266000	4.112606000	-0.826869000
1	8.865585000	-1.669986000	-0.844525000
1	9.040750000	0.049470000	-0.570467000
28	0.856861000	-0.634089000	-1.833873000
6	-2.099933000	-2.367910000	1.838610000
1	-5.450358000	6.968218000	1.748906000
7	-4.438671000	6.931410000	1.746171000
6	-3.810576000	5.765234000	1.632328000
7	-4.592139000	4.658917000	1.524169000
6	-3.975185000	3.510826000	1.340785000
7	-2.571205000	3.491446000	1.246022000
6	-1.824116000	4.659804000	1.477836000
7	-2.451371000	5.802210000	1.655018000
7	-2.620897000	1.213406000	0.691095000
6	-3.909742000	1.260588000	1.028954000
7	-4.640849000	2.341856000	1.293106000
6	-1.921676000	2.313214000	0.914360000
7	-0.595627000	2.267765000	0.882809000
6	0.035567000	3.360807000	1.313534000
7	-0.483284000	4.562949000	1.552903000
7	1.405687000	3.229154000	1.535000000
6	2.083560000	2.074087000	1.793547000
7	3.417562000	2.141079000	1.794516000
6	4.134735000	1.021262000	1.993343000
7	3.438243000	-0.185550000	2.195251000
7	1.366414000	0.945408000	2.072937000

6	2.096318000	-0.132662000	2.236471000
6	4.087282000	-1.433669000	2.309001000
7	5.445776000	0.982143000	1.987687000
6	6.026368000	-0.239938000	2.155326000
7	7.348894000	-0.273802000	2.170291000
7	5.391766000	-1.447589000	2.298949000
7	3.334167000	-2.559521000	2.392045000
6	2.018430000	-2.438414000	2.395004000
7	1.385440000	-1.243284000	2.424186000
7	1.217675000	-3.554082000	2.376885000
1	1.683843000	-4.459716000	2.406629000
7	-0.782437000	-2.381037000	2.066854000
6	-0.150298000	-3.571044000	2.197293000
7	-0.768147000	-4.737612000	2.152767000
6	-2.104435000	-4.755941000	1.929263000
7	-2.794592000	-5.862704000	1.873065000
6	-4.138069000	-5.744540000	1.633637000
7	-4.839257000	-4.594453000	1.440671000
7	-4.822649000	-6.876998000	1.585252000
7	-2.780283000	-3.532768000	1.749328000
6	-4.164431000	-3.469128000	1.493048000
7	-4.724003000	-2.264148000	1.291484000
6	-3.952842000	-1.176923000	1.373457000
7	-2.634367000	-1.181221000	1.708618000
7	-4.553195000	0.028964000	1.130344000
1	-3.908308000	7.787664000	1.847872000
1	1.953464000	4.085825000	1.567462000
1	7.884037000	0.579966000	2.061469000
1	7.836439000	-1.154568000	2.287491000
1	-5.570318000	0.031287000	1.115491000
1	-4.354076000	-7.764324000	1.726068000
1	-5.821695000	-6.862855000	1.416617000

8. References

[1] A. Call, C. Casadevall, F. Acuña-Parés, A. Casitas, J. Lloret-Fillol, *Chem. Sci.* **2017**, *8*, 4739–4749.

[2] K. J. Laidler, M. C. King, *J. Phys. Chem.* **1983**, *87*, 2657–2664.

[3] J. Uppenbrink, *Science.* **1998**, *279*, 1831.

[4] A. Savini, A. Bucci, G. Bellachioma, L. Rocchigiani, C. Zuccaccia, A. Llobet, A. Macchioni, *Eur. J. Inorg. Chem.* **2014**, *2014*, 690–697.

[5] Gaussian 16, Revision C.01, M. J. Frisch, G. W. Trucks, H. B. Schlegel, G. E. Scuseria, M. A. Robb, J. R. Cheeseman, G. Scalmani, V. Barone, G. A. Petersson, H. Nakatsuji, X. Li, M. Caricato, A. V. Marenich, J. Bloino, B. G. Janesko, R. Gomperts, B. Mennucci, H. P. Hratchian, J. V. Ortiz, A. F. Izmaylov, J. L. Sonnenberg, D. Williams-Young, F. Ding, F. Lipparini, F. Egidi, J. Goings, B. Peng, A. Petrone, T. Henderson, D. Ranasinghe, V. G. Zakrzewski, J. Gao, N. Rega, G. Zheng, W. Liang, M. Hada, M. Ehara, K. Toyota, R. Fukuda, J. Hasegawa, M. Ishida, T. Nakajima, Y. Honda, O. Kitao, H. Nakai, T. Vreven, K. Throssell, J. A. Montgomery, Jr., J. E. Peralta, F. Ogliaro, M. J. Bearpark, J. J. Heyd, E. N. Brothers, K. N. Kudin, V. N. Staroverov, T. A. Keith, R. Kobayashi, J. Normand, K. Raghavachari, A. P. Rendell, J. C. Burant, S. S. Iyengar, J. Tomasi, M. Cossi, J. M. Millam, M. Klene, C. Adamo, R. Cammi, J. W. Ochterski, R. L. Martin, K. Morokuma, O. Farkas, J. B. Foresman, and D. J. Fox, Gaussian, Inc., Wallingford CT, 2016.

[6] A. D. Becke, *J. Chem. Phys.* **1993**, *98*, 5648–5652.

[7] C. Lee, W. Yang, R. G. Parr, *Phys. Rev. B* **1988**, *37*, 785–789.

[8] M. Dolg, U. Wedig, H. Stoll, H. Preuss, *J. Chem. Phys.* **1987**, *86*, 866–872.

[9] P. C. Hariharan, J. A. Pople, *Theor. Chim. Acta* **1973**, *28*, 213–222.

[10] W. J. Hehre, R. Ditchfield, J. A. Pople, *J. Chem. Phys.* **1972**, *56*, 2257–2261.

[11] A. V. Marenich, C. J. Cramer, D. G. Truhlar, *J. Phys. Chem. B* **2009**, *113*, 6378–6396.

[12] M. Svensson, S. Humbel, R. D. J. Froese, T. Matsubara, S. Sieber, K. Morokuma, *J. Phys. Chem.* **1996**, *100*, 19357–19363.

[13] A. D. McLean, G. S. Chandler, *J. Chem. Phys.* **1980**, *72*, 5639–5648.

[14] R. Krishnan, J. S. Binkley, R. Seeger, J. A. Pople, *J. Chem. Phys.* **1980**, *72*, 650–654.

[15] M. Claros, F. Ungeheuer, F. Franco, V. Martin-Diaconescu, A. Casitas, J. Lloret-Fillol, *Angew. Chemie* **2019**, *131*, 4923–4928.

[16] P. Winget, C. J. Cramer, D. G. Truhlar, *Theor. Chem. Acc.* **2004**, *112*, 217–227.

[17] S. Dapprich, I. Komáromi, K. S. Byun, K. Morokuma, M. J. Frisch, *J. Mol. Struct. THEOCHEM* **1999**, *461–462*, 1–21.

[18] T. Vreven, K. Morokuma, *Annu. Rep. Comput. Chem.* **2006**, *2*, 35–51.

End of Supporting Information
